# A haplotype-resolved chromosome-level genome assembly of autotetraploid Chinese yam (*Dioscorea polystachya*) elucidates dioscin biosynthesis and regulation

**DOI:** 10.1093/hr/uhaf344

**Published:** 2025-12-11

**Authors:** Nan Shan, Yao Xiao, Tianyao Li, Putao Wang, Asjad Ali, Jingyu Sun, Shenglin Wang, Qianglong Zhu, Tianxu Cao, Sha Luo, Jiali Lin, Zihao Li, Qinghong Zhou, Yingjin Huang

**Affiliations:** Jiangxi Province Key Laboratory of Vegetable Cultivation and Utilization, Jiangxi Agricultural University, Nanchang 330045, China; Jiangxi Province Key Laboratory of Vegetable Cultivation and Utilization, Jiangxi Agricultural University, Nanchang 330045, China; State Key Laboratory of Vegetable Biobreeding, Key Laboratory of Biology and Genetic Improvement of Horticultural Crops of the Ministry of Agriculture and Rural Affairs, Institute of Vegetables and Flowers, Chinese Academy of Agricultural Sciences, Beijing 100081, China; Biomedical Research Institute, Hunan University of Medicine, Huaihua 418000, China; Queensland Department of Agriculture and Fisheries, P.O. Box 1054, Mareeba, QLD 4880, Australia; Jiangxi Province Key Laboratory of Vegetable Cultivation and Utilization, Jiangxi Agricultural University, Nanchang 330045, China; Jiangxi Province Key Laboratory of Vegetable Cultivation and Utilization, Jiangxi Agricultural University, Nanchang 330045, China; Jiangxi Province Key Laboratory of Vegetable Cultivation and Utilization, Jiangxi Agricultural University, Nanchang 330045, China; College of Modern Agriculture and Bioengineering, Yangtze Normal University, Chongqing 408100, China; Jiangxi Province Key Laboratory of Vegetable Cultivation and Utilization, Jiangxi Agricultural University, Nanchang 330045, China; Jiangxi Province Key Laboratory of Vegetable Cultivation and Utilization, Jiangxi Agricultural University, Nanchang 330045, China; Jiangxi Province Key Laboratory of Vegetable Cultivation and Utilization, Jiangxi Agricultural University, Nanchang 330045, China; Jiangxi Province Key Laboratory of Vegetable Cultivation and Utilization, Jiangxi Agricultural University, Nanchang 330045, China; Jiangxi Province Key Laboratory of Vegetable Cultivation and Utilization, Jiangxi Agricultural University, Nanchang 330045, China; Key Laboratory of Crop Physiology, Ecology and Genetic Breeding (Jiangxi Agricultural University), Ministry of Education of China, Nanchang 330045, China

## Abstract

Chinese yam (*Dioscorea polystachya*) is extensively cultivated for nutritional and medicinal applications. However, the lack of a high-quality reference genome has hindered molecular genetic analysis and breeding advancements. Here, we present a haplotype-resolved chromosome-level assembly for this autotetraploid species, featuring a 1.56-Gb genome anchored to 80 chromosomes across four haplotypes and comprising 95 668 protein-coding genes. Following divergence from *Dioscorea alata* about 4.64 million years ago (Mya), *D. polystachya* underwent a specific whole-genome duplication ~1.42 Mya, resulting in an autotetraploid species without subgenomic dominance. Notably, the biosynthetic pathway genes of dioscin, an important steroidal saponin primarily accumulating in tubers, were generally over-retained in *D. polystachya* compared to the diploid species *D. alata*. Of these genes, *7-dehydrocholesterol reductase* (*Dp7-DR*) promoted the accumulation of dioscin, exhibiting tuber-specific expression and strong inducibility by abscisic acid, based on transcriptome and gene function analyses. We determined that the transcription factor DpbZIP12 activates *Dp7-DR* transcription, as supported by yeast one-hybrid, dual-luciferase reporter, and electrophoretic mobility shift assays. Notably, overexpressing *Dp7-DR* or *DpbZIP12* resulted in lower cholesterol levels and elevated dioscin levels, while silencing either gene produced opposite metabolic profiles. These findings delineate promising targets for manipulating dioscin content and expand genetic resources for enhancing yam nutritional quality.

## Introduction

China is a major and ancient origin and domestication center of yam (*Dioscorea* spp.), with rich germplasm resources representing 55 species [1]. Among these, *Dioscorea polystachya* is the most widely distributed and cultivated species, valued for centuries as both a healthy vegetable and a source of traditional Chinese medicine because it is rich in high-quality starch and multiple functional compounds. Dioscin, a saponin from *D. polystachya*, serves as an important industrial precursor for synthesizing steroid hormones such as cortisone, pregnenolone, and progesterone [2], and it exhibits beneficial effects, including anti-inflammatory, hypoglycemic and anti-tumor activities [3, 4]. In plants, the biosynthesis of dioscin starts with isopentenyl diphosphate and dimethylallyl diphosphate produced by the mevalonate pathway and methylerythritol 4-phosphate pathways, respectively [5, 6]. These intermediates are subsequently converted into geranyl diphosphate and farnesyl pyrophosphate, leading to the production of squalene, 2,3-oxidosqualene, and cholesterol. Cholesterol serves as a precursor for steroidal saponins and steroidal alkaloids [7], which is modified by cytochrome P450 monooxygenases (CYP90B, CYP94, CYP72A *et al.*) and UDP-glycosyltransferases [8–11]. Although heterologous biosynthesis from cholesterol to diosgenin has been achieved in *Nicotiana benthamiana* and yeast [12, 13], the absence of an efficient transgenic system in *Dioscorea* species has hindered the functional validation of related genes. Furthermore, due to the dioecious reproductive system and clonal propagation characteristics of *D. polystachya* [14], a high-quality reference genome for this species remains unavailable. These limitations pose significant challenges for elucidating the regulatory mechanisms of the genes encoding key enzymes involved in dioscin biosynthesis in *D. polystachya*.

In recent studies, genome assemblies published for diploid accessions of *Dioscorea rotundata* [15], *Dioscorea alata* [16, 17], *Dioscorea zingiberensis* [6], and the Chinese herb *Dioscorea nipponica* [18] have provided valuable resources for understanding the mechanism of dioscin biosynthesis. However, intraspecific variation in ploidy levels is widespread across *Dioscorea* species. For instance, documented cytotypes for white yam (*D. rotundata*) and *D. alata* encompass diploid (2*n* = 2*x* = 40), triploid (2*n* = 3*x* = 60), and tetraploid (2*n* = 4*x* = 80) forms [15, 16, 19, 20], whereas the Chinese medicinal species *D. zingiberensis* has been reported to exhibit diploid (2*n* = 2*x* = 20), triploid (2*n* = 3*x* = 30), and tetraploid (2*n* = 4*x* = 40) cytotypes [5, 6, 14]. Nevertheless, the mechanisms underlying polyploidization and the evolutionary relationships among *Dioscorea* species with different ploidy levels remain to be fully characterized.

The *D. polystachya* cultivar ‘Ruichang’, originating in the Jiangxi Province more than 500 years ago, is distinguished among Chinese cultivars for its superior flavor. The tubers of this cultivar accumulate substantial carbohydrate reserves and abundant steroidal saponins [1]. Because of the value of these secondary metabolites, there has been great interest in understanding their biosynthetic pathways and the factors that influence their accumulation. In this study, we present a haplotype-resolved chromosome-level genome assembly of the autotetraploid *D. polystachya* cultivar Ruichang. Through integrated comparative genomic, transcriptomic, and gene functional analyses, we explored and elucidated the regulatory mechanisms behind dioscin biosynthesis, substantially advancing our mechanistic understanding of this pathway.

## Results

### Genome assembly and annotation

The stem of the Chinese yam (*D. polystachya* ‘Ruichang’) is slender, green, or purplish, and it exhibits a dextrorse (clockwise) twining growth habit. The leaves are typically opposite, heart shaped to triangular ovate. Bulbils (aerial tubers) commonly form in the leaf axils (Fig. S1A). The tubers are cylindrical, measuring ~60–80 cm in length and 6–8 cm in diameter, with yellowish brown skin and white flesh (Fig. S1B and C). The *D. polystachya* genome size was estimated to be ~1.6 Gb by flow cytometry; in addition, a *k*-mer distribution analysis indicated that the *D. polystachya* genome is tetraploid with a high heterozygosity level of 3.9% (Fig. S2A and B; Tables S1 and S2). We detected 80 chromosomes using the root tip squash method (Fig. S2C), suggesting the presence of 20 chromosomes for each haplotype (2*n* = 4*x* = 80).

**Figure 1:**
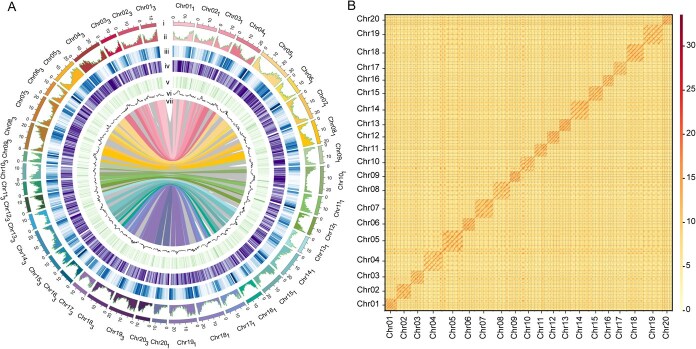
Distribution of genomic features within the *D. polystachya* genome. (A) Genomic landscape of *D. polystachya*. Chromosomes of yam (i), gene density (ii), TE density (iii), expression level (iv), noncoding RNA content of the genome (v), GC content (vi), paralogous genes on different chromosomes (vii) were displayed; 500-kb sliding windows. (B) Genome-wide all-by-all Hi-C interaction heatmap. Four haplotypes (Hap1, Hap2, Hap3, and Hap4) from each chromosome are shown.

To assemble the genome sequence of *D. polystachya*, we generated 41.20 Gb of HiFi long reads and 76.47 Gb of short reads (Table S3) and obtained a draft assembly comprising four haplotypes using hifiasm. This assembly was 1.56 Gb in length, with a contig N50 value of 4.83 Mb after removing redundant contigs. Using 268.12 Gb (~175×) of chromosome conformation capture (Hi-C) data, we independently scaffolded those contigs belonging to each haplotype into four sets of 20 pseudochromosomes (Table 1; Table S4). Briefly, we aligned Hi-C reads to the draft assembly and partitioned them according to their best matching haplotype. Guided by the resulting haplotype-partitioned Hi-C interactions, we anchored and oriented all contigs into 20 clusters per haplotype. We then used a merged Hi-C contact map to refine scaffolding among the four haplotypes, with manual adjustments to correct misassigned genomic regions. Finally, we generated a haplotype-phased genome assembly for *D. polystachya* (Fig. 1). Each of these four haplotypes (hereafter referred to as Hap1, Hap2, Hap3, and Hap4) consists of 20 pseudochromosomes with total sizes of 393.53, 380.31, 325.91, and 323.70 Mb, respectively (Table 1). Comparisons to the diploid genome of *D. alata* showed that they are highly colinear (Fig. S3). Moreover, each chromosome in *D. alata* had four corresponding syntenic pseudochromosomes in the *D. polystachya* genome, each representing one of the four haplotypes (Fig. S3).

**Table 1:** Assembly and annotation statistics.

**Statistics**	**Value**
Genome assembly	
Contig sequence total/count	1562.96 Mb/653
Contig N50 length/count	4.83 Mb/105
Contig N90 length/count	1.32 Mb/351
Scaffolds length total/count	1437.24 Mb/80
Scaffold N50 length/count	19.04 Mb/32
Scaffold N90 length/count	13.53 Mb/68
Hap1 total/count	393.53 Mb/116
Hap2 total/count	380.31 Mb/117
Hap3 total/count	325.91 Mb/109
Hap4 total/count	323.70 Mb/120
Genome annotation	
Total predicted gene/annotation gene	98 699/95 668
Repetitive sequences	887.18 Mb/61.88%
Number of genes with GO annotation	68 615
Number of genes with KOG annotation	82 632
Number of genes with NR annotation	95 647
Number of genes with Swiss annotation	70 412
Number of genes with TrEMBL annotation	93 331

We assessed the completeness of our genome assembly by conducting a Benchmarking Universal Single-Copy Orthologs (BUSCO) analysis. We identified 96.4% of the 1614 BUSCO genes in the *D. polystachya* genome as complete single-copy or duplicated genes (Table S5). For predicting protein-coding genes, we integrated homology-based, *de novo*, and transcriptome-based analyses, yielding 98 699 predicted gene models with an average gene length of 3440 bp, a mean exon length of 252 bp, and 5.25 exons per gene (Table 1). We assigned functional annotations to 95 668 (or 96.9% of total) genes using public databases (Table 1; Fig. S4). Repetitive sequences spanned 887.18 Mb across the four haplotypes, constituting 61.9% of the assembled genome (Table 1).

### Disentangling the differential expression of *D. polystachya* haplotypes

**Figure 2:**
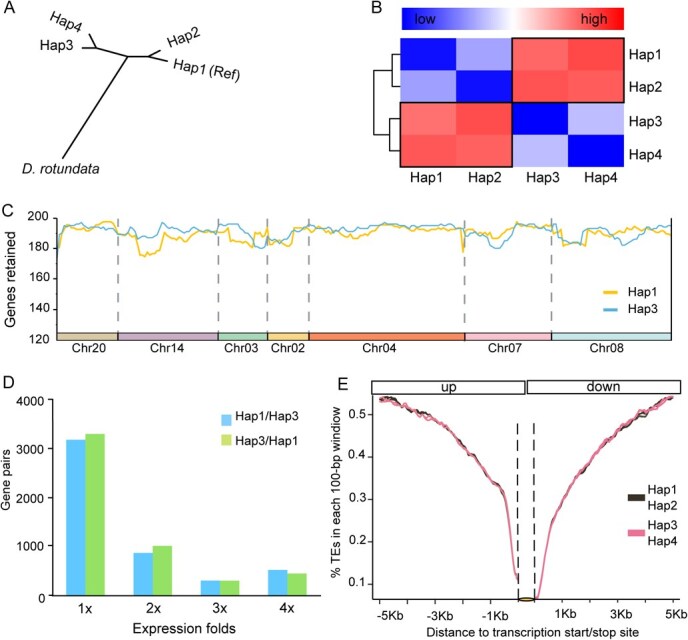
Chromosome phasing and subgenome dominance of *D. polystachya*. (A) Clustering of 4 937 003 chromosome-specific SNP enabled consistent partitioning of the genome into two clusters. *Dioscorea rotundata* was used as outgroup, and Hap1-Hap4 donate the four sets of chromosomes after phasing. Chr01 is displayed as a representative example, with the trends observed across most chromosomes. (B) Heatmaps of variation between subgenomes. Each cell represents the SNP density and InDel relationship between two subgenomes, with the color depth reflecting the degree of variation. (C) Variation in gene density between representative chromosomes in the two subgenomes of *D. polystachya*. Gene density variation along representative chromosomes without substantial segmental fragmentation in the two subgenomes of *D. polystachya* is shown. Gene density was calculated using 200-kb sliding windows. (D) Number of dominantly expressed genes between paralogous gene pairs from the two subgenomes of *D. polystachya*. Blue indicates Hap1 genes with higher expression than their Hap3 paralogs (Hap1/Hap3 fold change >1, 2, 3, or 4); green indicates Hap3 genes with higher expression than their Hap1 paralogs (Hap3/Hap1 fold change >1, 2, 3, or 4). (E) TE distribution in flanking regions of genes from different gene sets. TE density was calculated in 100-bp sliding windows over the 5-kb region upstream and downstream of the transcription start site. The yellow ellipse indicates the gene body.

To investigate the evolutionary relationships among the four haplotypes for each chromosome of the *D. polystachya* genome, we compared the *D. polystachya* genome to the *D. rotundata* genome and identified 4 937 003 single nucleotide polymorphisms (SNPs) in total. We then used these SNPs to reconstruct phylogenetic trees for each set of pseudochromosomes. The Hap1 and Hap2 versions of most (16/20) pseudochromosomes were phylogenetically close to each other, as were those of Hap3 and Hap4 pseudochromosomes (Fig. S5), suggesting two major haplotype lineages, represented by Hap1/Hap2 and Hap3/Hap4 (Fig. 2A). We identified sequence variations (including SNPs and insertions/deletions [InDels]) between all haplotype pairs to validate the genetic divergence of these four haplotypes. The numbers of genomic variants between Hap1 and Hap2 and between Hap3 and Hap4 were significantly smaller than those obtained for all other combinations (Fig. 2B; Tables S6 and S7). These consistent phylogenetic and genetic patterns among the four haplotypes support the reliability of the haplotype phasing.

Given the phylogenetic clustering of haplotypes (Hap1/Hap2 and Hap3/Hap4 as homologous pairs) (Fig. 2A and B), we designated Hap1 and Hap3 as representatives of two ‘diploid’ pseudogenomes in the tetraploid genome of *D. polystachya* and evaluated their divergence levels. Hap1 and Hap3 contain 25 679 and 24 056 genes, respectively. To mitigate effects from structural variation, we analyzed syntenic regions, identifying 17 258 genes in Hap1 and 17 356 in Hap3, indicating that Hap1 did not have a higher gene content (i.e. did not retain more genes) than Hap3 (Fig. 2C). Furthermore, we evaluated whether transcript levels are balanced. Using transcriptome deep sequencing (RNA-seq) data for *D. polystachya* tubers, we identified 20 681 genes expressed in Hap1 and 20 056 genes expressed in Hap3. Importantly, there was no significant bias in gene expression (transcripts per million [TPM] ≥ 2) levels between the syntenic paralogous pairs in Hap1 and Hap3 (Fig. 2D; Table S8). We also determined the density of transposable elements (TEs) in the syntenic regions of Hap1 and Hap3. The TE density in gene-flanking regions was also similar between these two pseudogenomes (Fig. 2E).

**Figure 3:**
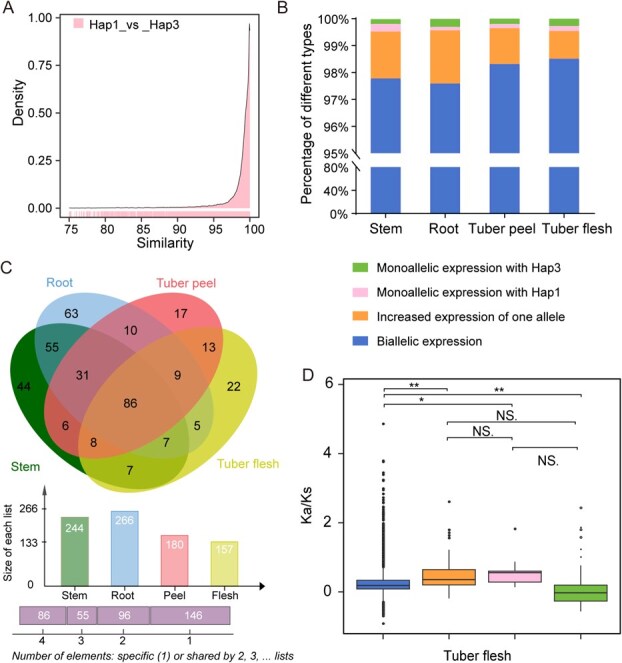
Allele-specific expression characteristics of *D. polystachya*. (A) Comparison of allele coding sequences between two haplotypes (Hap1 and Hap3). (B) Allele-specific expression (ASE) patterns in stem, roots, tuber peels, and tuber flesh. Genes with TPM >2 for both Hap1 and Hap3 alleles and less than 2-fold difference were classified as having ‘biallelic expression’. Genes with TPM >2 for both alleles but a >2-fold difference were defined as showing ‘increased expression of one allele’. When only one allele (either Hap1 or Hap3) had TPM >2 and the other ≤2, the gene was defined as showing ‘Hap1 dominant expression’ or ‘Hap3 dominant expression’, respectively. (C) Venn diagram showing the overlap of ASE genes in the four tissues. (D) Calculation of *Ka/Ks* values for various classes of ASE genes in tuber flesh, with outliers represented by dots and the interquartile range shown by boxes. ^*^P ≤ 0.05; ^**^P ≤ 0.001 (*t*-test).

A comparative analysis of genes from Hap1 and Hap3 revealed very high sequence conservation, with over 95% of all gene pairs across the two haplotypes exhibiting very high (>95%) sequence identity (Fig. 3A). Moreover, when we checked for potential expression bias between the two haplotypes in various tissues (stems, roots, tuber peel, and tuber flesh), we determined that 2.2%, 2.4%, 1.7%, and 1.5% of expressed genes show haplotype-specific expression in the stems, roots, tuber peels, or tuber flesh, respectively (Fig. 3B; Table S9). In addition, 86 expressed genes displayed significant differences in their expression levels in a haplotype-specific manner among the four tissues analyzed (Fig. 3C). Genes with biallelic expression had notably lower *Ka/Ks* values than all other genes (Fig. 3D), suggesting that most of these genes with balanced expression between the two haplotypes Hap1 and Hap3 are more evolutionarily conserved than the other types of genes with imbalanced expression between the two haplotypes. Collectively, these findings suggest that the *D. polystachya* genome shows no clear expression dominance among haplotypes.

### Analysis of whole-genome duplication events and phylogenetic relationships of *D. polystachya*

**Figure 4:**
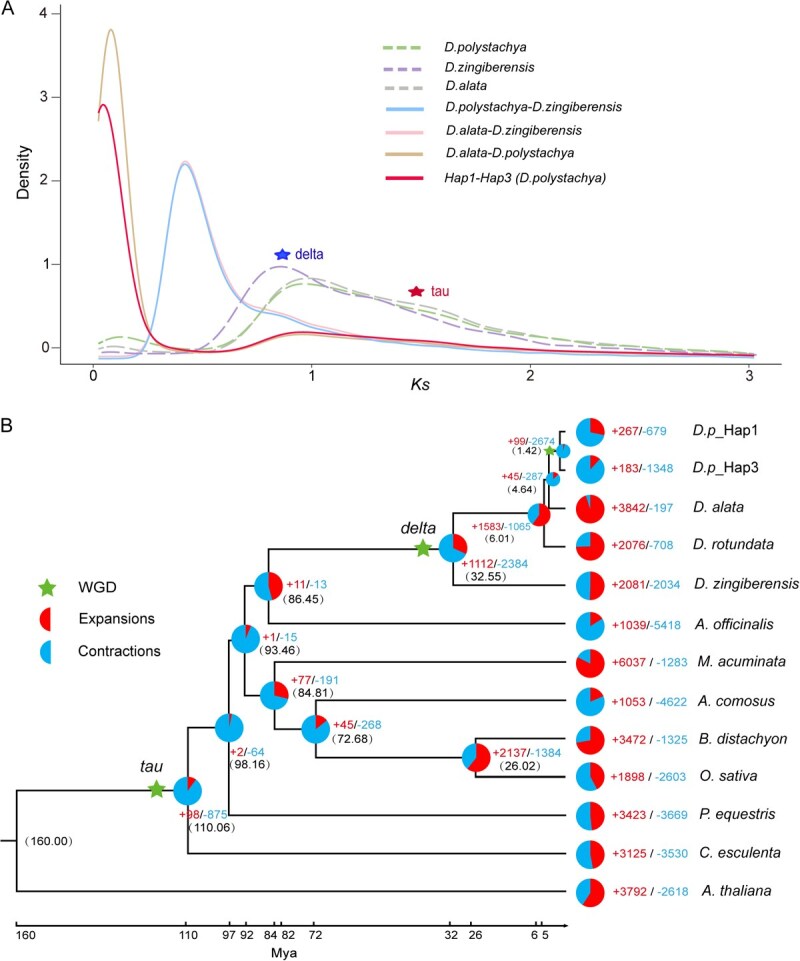
Auto-tetraploidization event in *D. polystachya*. (A) *Ks* values revealed a recent *D. polystachya*-specific WGD event during the evolution. (B) Phylogenetic tree for *D. polystachya* and eleven other plants. Gene family expansions and gene family contractions among the total changes are shown in the pie chart, the estimated divergence time (million years ago, Mya) is indicated at each node, pentagrams represent three WGD events.

To investigate *D. polystachya* evolution, we assessed the distribution of synonymous substitution per gene (*Ks*) between colinear gene pairs to identify signatures of whole-genome duplication (WGD) events. Based on the peaks of *Ks* values, we detected evidence for three WGD events in *D. polystachya*. A recent *D. polystachya*-specific tetraploidization event (*Ks* = 0.020) reflected the divergence time between Hap1/2 and Hap3/4. In addition, two shared WGD events corresponded to the *delta* (*Ks* = 0.82) and *tau* (*Ks* = 1.18) events, which are conserved in Dioscoreaceae and monocotyledonous species, respectively (Fig. 4A). We also identified a divergence between *D. polystachya* and *D. alata* (*Ks* = 0.063) (Fig. 4A). Collectively, the *D. polystachya-*specific WGD event resulted in autotetraploidization, confirming the Chinese yam variety ‘Ruichang yam’ as an autotetraploid.

We reconstructed a time-calibrated phylogeny of 12 plant species, including *D. alata*, *D. rotundata*, and *D. zingiberensis*, using 923 shared single-copy genes. Phylogenomic analysis dated the divergence of Dioscoreaceae from other monocot lineages to ~86.45 million years ago (Mya). *Dioscorea zingiberensis* separated from the *Dioscorea* group at ~32.55 Mya, whereas *D. polystachya* and its closest relative *D. alata* diverged ~4.64 Mya (Fig. 4B). Molecular dating placed the *tau*, *delta*, and *D. polystachya*-specific tetraploidization WGD events at 110, 32, and 1.42 Mya, respectively (Fig. 4B).

Using syntenic gene pairs between the *D. alata* and *D. polystachya* genomes, we estimated a background gene retention rate of 91.3%, calculated as the ratio of *D. polystachya* syntenic genes to (*D. alata* syntenic genes × 4). This high gene retention rate is consistent with the relatively recent divergence time between *D. polystachya* and *D. alata*. Notably, genes involved in the dioscin biosynthetic pathway showed an even higher retention rate of 98.4%, suggesting that nearly all these genes are preserved as four copies (one per haplotype) in the *D. polystachya* genome. Moreover, the copy number of six gene families involved in this pathway was further expanded through tandem duplication events, namely the *GGPP*, *24-Sterol methyltransferase type 1* (*SMT1*), *C-4 Sterol methyl oxidase 1* (*SMO1*), *CYP72A*, *CYP90B*, and *CYP94* gene families (Table S11). In addition, based on RNA-seq data of tuber tissues, we observed frequent divergence in haplotype expression among these genes across the four haplotypes (Table S11). These findings indicate that the tetraploidization that led to the formation of *D. polystachya* might have contributed to enhanced dioscin biosynthesis compared to that in *D. alata* through both higher gene copy number and diversified gene expression patterns.

### Role of *Dp7-DR* in dioscin accumulation in *D. polystachya*

**Figure 5:**
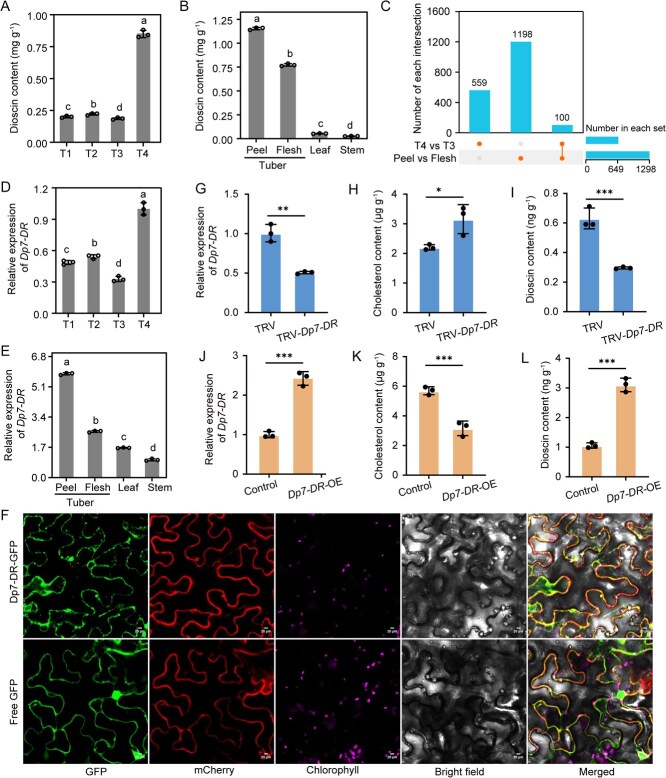
Identification of key genes related to dioscin biosynthesis. (A, B) Dioscin content in *D. polystachya* tubers at different developmental stages (A) and in different tissues (B). T1–T4 indicate four rapid growth stages (150, 170, 190, and 210 days after planting). Values are shown as means ± standard deviation from three biological replicates (*n* = 3). Different lowercase letters indicate significant differences, as determined by one-way analysis of variance (ANOVA), with *P* < 0.05. (C) Number of DEGs for the indicated comparisons. The *x*-axis of the UpSet plot represents the total number of DEGs identified in each group, and the *y*-axis shows the number of DEGs unique to each set across various tissues. (D, E) Relative *Dp7-DR* expression levels in yam tubers at different developmental stages (D) and in diverse tissues (E), as determined by RT-qPCR. The specific primers are listed in Table S2; *ACTIN* was used as a reference. Three biological replicates were used for each sample, and the 2^−ΔΔCt^ method was used to measure transcript levels. (F) Subcellular localization of Dp7-DR in *N. benthamiana* leaves. Confocal microscopy images of leaves expressing Dp7-DR-GFP (top) or GFP alone (bottom) were acquired using an LSM 880 confocal microscope (Zeiss, Germany) with the following settings: GFP (excited at 488 nm), ER marker SPER-mCherry (excited at 561 nm), and chlorophyll autofluorescence (excited at 640 nm). GFP, green fluorescent protein; SPER-mCherry, an ER marker; Chlorophyll, chlorophyll autofluorescence. Merged, image combining GFP fluorescence, ER fluorescence, chlorophyll autofluorescence, and bright field. Scale bars, 20 μm. (G–L) Relative expression levels of *Dp7-DR* (G, J), cholesterol content (H, K), and dioscin content (I, L) in *D. polystachya* bulbils with *Dp7-DR* silencing (G–I) or overexpression (J–L). Values are shown as means ± standard deviation from three replicates (*n* = 3). TRV, co-infiltration of the pTRV1 vector with the empty vector pTRV2. TRV-*Dp7-DR*, co-infiltration of pTRV1 with pTRV2-*Dp7-DR*. ^*^P < 0.05; ^**^P < 0.01; ^***^P < 0.001 (*t*-test).

We conducted a temporal RNA-seq analysis of four tuber developmental stages, at 150 (T1), 170 (T2), 190 (T3), and 210 (T4) days after planting, using Hap1 of the *D. polystachya* genome as a reference (Fig. S6). A total of 8.65 Gb of clean reads was produced on average, reaching an average mapping ratio to the reference genome of 96.0% (Table S12). We identified differentially expressed genes (DEGs) through pairwise comparisons of the four developmental stages (Fig. S6B) and divided them into nine co-expression clusters based on *K*-means clustering analysis (Fig. S6C). Our previous study revealed rapid tuber development from T2 to T4 stages [21]. Notably, the DEGs in Clusters 4 and 6 were highly expressed at the T4 stage and wereenriched in Kyoto Encyclopedia of Genes and Genomes (KEGG) pathways related to ‘steroid biosynthesis’ and ‘plant hormone signal transduction’ (Fig. S6D). Importantly, the levels of dioscin, the primary bioactive steroid saponin in yam tubers, peaked at the T4 stage (Fig. 5A), with tuber peel and flesh accumulating significantly higher concentrations than leaves or stems, and tuber peels having higher levels than tuber flesh (Fig. 5B).

To pinpoint key genes responsible for dioscin accumulation, we compared DEGs in tubers at Stages T3 and T4, as well as in the peel and flesh tissues, identifying 659 and 1298 DEGs, respectively, with 100 shared between the comparisons (Fig. 5C). Among these, *Dp7-DR* (DpolyH1c03G000046), encoding 7-dehydrocholesterol reductase (7-DR), is annotated to be the ‘steroid biosynthesis’ KEGG pathway (ko00100) (Fig. S7A). Expression analysis of dioscin biosynthetic pathway genes (Table S10) across the four haplotypes of *D. polystachya* revealed that all four copies of *Dp7-DR* (*DpolyH1c03G000046*, *DpolyH2c03G000045*, *DpolyH3c03G000049*, and *DpolyH4c03G000003*) exhibited high transcript levels, which correlated strongly with dioscin content (Fig. S7B and C; Table S13). These four haplotype sequences were identical (Fig. S8A). We isolated a 1305-bp *Dp7-DR* open reading frame (Fig. S8B) encoding a 434-amino acid protein that phylogenetically clusters with its orthologs Da7-DR from *D. alata* and Dr7-DR from *D. rotundata* (Fig. S8C; Table S14). *Dp7-DR* expression peaked at the T4 developmental stage, particularly in tuber peels and flesh (Fig. 5D and E), consistent with the elevated dioscin accumulation in these tissues. Furthermore, intracellular localization via Dp7-DR-GFP fusion in *N. benthamiana* leaves showed GFP signal colocalization with an endoplasmic reticulum (ER) marker (Fig. 5F), indicating predominant ER localization. This localization is consistent with its function as a key enzyme in the sterol biosynthesis pathway, thereby facilitating its role in dioscin production.

To investigate the function of *Dp7-DR* in dioscin biosynthesis, we employed virus-induced gene silencing (VIGS) and transient overexpression in *D. polystachya* bulbils (Fig. 5G–L and Fig. S9). Compared to the control, *Dp7-DR*-silenced bulbils exhibited significant reduction in *Dp7-DR* transcripts (Fig. 5G), increased cholesterol content, and decreased dioscin content (Fig. 5H and I). Conversely, overexpressing *Dp7-DR* in bulbils was characterized by reduced cholesterol and enhanced dioscin accumulation (Fig. 5J–L). Collectively, these results indicate that *Dp7-DR* plays a role in dioscin accumulation.

### DpbZIP12 binds to the *Dp7-DR* promoter to promote its transcription

**Figure 6:**
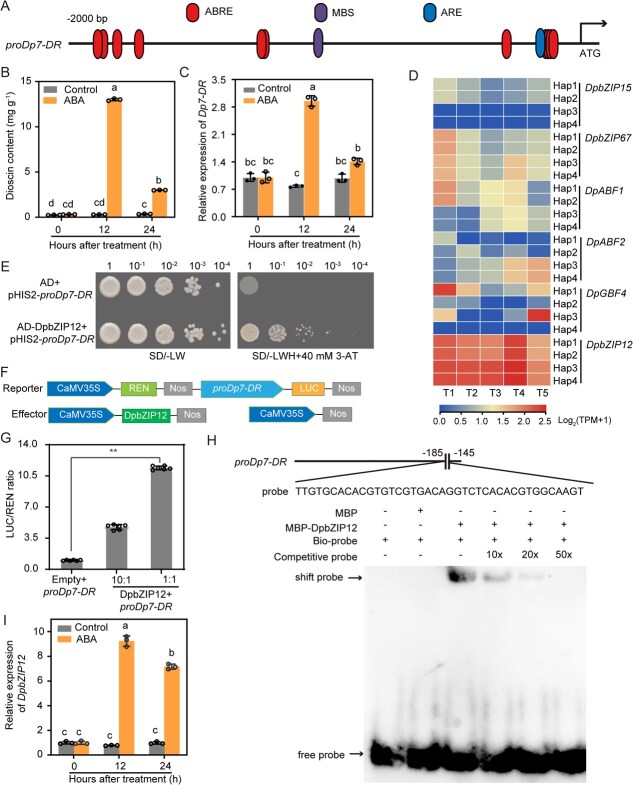
DpbZIP12 binds directly to the *Dp7-DR* promoter to activate its expression. (A) Prediction of *cis*-acting elements in the *Dp7-DR* gene promoter, as determined by the PlantCARE database. (B, C) Dioscin content (B) and relative *Dp7-DR* expression levels (C) in response to treatment with 100 μM ABA. Values are shown as means ± standard deviation from three independent biological replicates. Different lowercase letters indicate significant differences, as determined by one-way ANOVA, with *P* < 0.05. (D) Heatmap representation of expression levels for the genes encoding subgroup A bZIP transcription factors for each of the four haplotypes at four tuber developmental stages (T1–T4). (E) Yeast one-hybrid assay showing that DpbZIP12 binds to the *Dp7-DR* promoter. The plasmids pHIS2-*proDp7-DR* and AD-DpbZIP12 were co-introduced into the yeast strain Y1H Gold. AD, GAL4 activation domain. SD/−LW, synthetic defined (SD) medium lacking leucine and tryptophan. SD/−LWH, SD medium lacking leucine, tryptophan, and histidine. 3-AT, 3-amino-1,2,4-triazole. (F) Diagrams of the plasmids used for the dual-luciferase reporter assays. The *Dp7-DR* promoter was cloned upstream of the firefly luciferase (*LUC*) reporter gene in pGreenII-0800-LUC, and *DpbZIP12* was cloned in pGreenII-62-SK as effector plasmid. (G) Dual-luciferase reporter assay system showing that DpbZIP12 activates transcription of the *Dp7-DR* promoter. The empty vector pGreenII-62-SK was used as negative control. *Agrobacterium* cell suspensions containing either pGreenII-62-SK-*DpbZIP12* or pGreenII-0800-LUC-pro*Dp7-DR* were mixed in 10:1 and 1:1 ratios (v/v). Values are shown as means ± standard deviation (*n* = 6). Statistical significance was determined by *t*-test for the comparison between the control and the 1:1 mixture of pGreenII-62-SK-*DpbZIP12* and pGreenII-0800-LUC-*proDp7-DR* (^**^P < 0.01). (H) EMSA showing the binding of recombinant purified MBP-DpbZIP12 to the *Dp7-DR* promoter. Biotin-labeled probes were mixed with MBP-DpbZIP12. Unlabeled probes served as competitor probes. MBP, maltose binding protein; *proDp7-DR*, *Dp7-DR* promoter. (I) Relative *DpbZIP12* expression levels in response to treatment with 100 μM ABA. Values are shown as means ± standard deviation from three independent replicates. ^*^P < 0.05; ^**^P < 0.01; ^***^P < 0.001 (*t*-test).

The promoter region of *Dp7-DR* contains 11 abscisic acid (ABA)-responsive elements (ABREs), as predicted by the PlantCARE database (Fig. 6A; Table S15). ABREs are involved in the response to ABA and can be recognized by basic leucine zipper (bZIP) A subfamily transcription factors. Treatment with ABA for 12 h resulted in a rise in dioscin content and higher expression levels for *Dp7-DR* (Fig. 6B and C). *DpbZIP12*, encoding a bZIP A subfamily member, was highly expressed in *D. polystachya* tubers based on RNA-seq data; moreover, its expression levels were strongly and positively correlated with dioscin content (Fig. 6D and Fig. S11A). To better understand the putative regulatory role of DpbZIP12 in dioscin accumulation, we investigated whether DpbZIP12 directly bound to the *Dp7-DR* promoter. Yeast one-hybrid (Y1H) assays confirmed that DpbZIP12 binds to the *Dp7-DR* promoter in yeast cells (Fig. 6E). We then assessed the effect of DpbZIP12 on *Dp7-DR* promoter activity using luciferase reporter assays (Fig. 6F). *Nicotiana benthamiana* leaves co-infiltrated with a *DpbZIP12* effector construct and a reporter construct consisting of the *Dp7-DR* promoter driving the firefly luciferase (*LUC*) reporter gene exhibited notably elevated luminescence over those measured in the controls (Fig. 6G). We also performed an electrophoretic mobility shift assay (EMSA) with a probe containing the bZIP-binding site in the *Dp7-DR* promoter. Recombinant purified maltose binding protein (MBP)-DpbZIP12 bound to the biotin-labeled probe, and the intensity of the shifted band diminished when an unlabeled competitor probe was added (Fig. 6H). These findings suggest that DpbZIP12 binds to the *Dp7-DR* promoter to induce its transcription.

**Figure 7:**
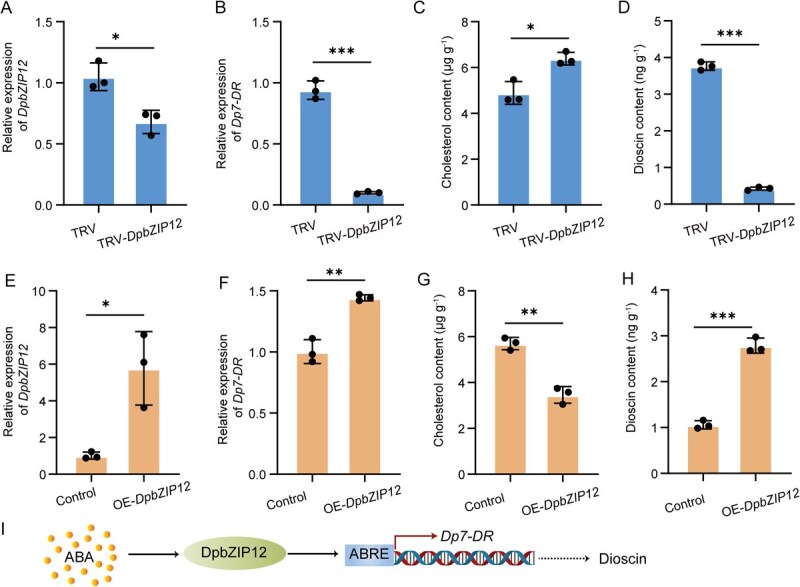
DpbZIP12 promotes the accumulation of dioscin in bulbils. (A–H) Relative expression levels of *DpbZIP12* (A, E) and *Dp7-DR* (B, F), cholesterol (C, G), and dioscin contents (D, H) of silenced (A–D) or overexpressing *DpbZIP12* (E–H). RT-qPCR was performed using the primers listed in Table S2; *ACTIN* was used as a reference. Three biological replicates were used for each sample, and the 2^−ΔΔCt^ method was used to measure transcript levels. Values are shown as means ± standard deviation from three replicates (*n* = 3). ^*^P < 0.05; ^**^P < 0.01; ^***^P < 0.001 (*t*-test). TRV, co-infiltration of pTRV1 with pTRV2 as negative control; TRV-*DpbZIP12*, co-infiltration of pTRV1 with pTRV2-*DpbZIP12*. (I) Proposed model of ABA-mediated regulation of dioscin biosynthesis, with DpbZIP12 activating *Dp7-DR* transcription.

ABA treatment elevated *DpbZIP12* expression levels within 12 h (Fig. 6I). Silencing *DpbZIP12* in bulbils resulted in significantly lower *Dp7-DR* transcript levels (Fig. 7A and B), leading to greater accumulation of cholesterol concomitant with lower dioscin levels (Fig. 7C and D). By contrast, overexpressing *DpbZIP12* led to lower cholesterol content, along with elevated dioscin content and higher *Dp7-DR* expression (Fig. 7E–H). These results demonstrate that DpbZIP12 activates transcription from the *Dp7-DR* promoter, thereby enhancing its expression and promoting dioscin biosynthesis.

## Discussion

### A recent WGD resulted in the formation of autotetraploid *D. polystachya* and contributed to dioscin biosynthesis

Polyploidization is closely linked to the diversification of plant species and plays an important role in plant genome evolution [22]. In addition to the *tau* and *delta* WGD events shared by monocotyledonous and *Dioscorea* species, respectively, *D. polystachya* experienced its own WGD event about 1.42 Mya after its divergence from *D. alata* (Fig. 4), the closest species in evolutionary terms among the yams whose genomes have been sequenced [23]. This recent WGD event resulted in the autotetraploidy of *D. polystachya*. Polyploidy confers several agronomically significant advantages in plant breeding, most notably larger organs, buffering against deleterious mutations, and greater heterozygosity. Harnessing these attributes is important for the development of cultivars with greater yields, enhanced product quality, and heightened tolerance to biotic and abiotic stressors [24–26]. For instance, tetraploid potato (*Solanum tuberosum*) and alfalfa (*Medicago sativa*) are more productive than their diploid counterparts [27, 28]. Polyploidy substantially augments carotenoid accumulation in maize (*Zea mays*), with the seeds of artificial tetraploid maize lines demonstrating concentrations up to 220% higher than diploid seeds [29]. A naturally occurring tetraploid *Arabidopsis* (*Arabidopsis thaliana*) accession exhibits enhanced salinity tolerance through polyploidy-mediated regulation of leaf potassium homeostasis [30]. Significant divergence in dioscin content exists between *D. polystachya* and its diploid ancestor *D. alata* [18], denoting polyploidy contributes to dioscin accumulation in the autotetraploidy of *D. polystachya* in this study.

One possible explanation to dioscin accumulation after WGD in *D. polystachya* is higher gene copy number and gene-dosage effects. Genes associated with the dioscin biosynthetic pathway were more prone to retention and are present in more copies in *D. polystachya* than in *D. alata* (Table S11). In hexaploid camelina (*Camelina sativa*), combinatorial sequence variation across its three *FATTY ACID DESATURASE 2* (*FAD2*) homoeologs induced by gene editing generated substantial phenotypic diversity, yielding oil profiles with oleic acid concentrations ranging from 10% to 62% among different edited lines, illustrating how oil content is correlated with allelic dosage in an additive manner [31]. Similarly, genome-wide association studies in sweet potato (*Ipomoea batatas*) revealed genomic regions associated with 23 agronomic characteristics and exhibiting dosage effects. Specifically, expression of the expansin gene *IbEXPA4* was negatively correlated with the dosage of its favorable allele, which suppressed *IbEXPA4* transcription and enhanced storage root weight. In the same study, tuber flesh coloration exhibited a positive dosage effect with *Orange* (*IbOr*) haplotypes derived from the ‘Nancy Hall’ lineage with orange flesh [32]. Besides, in *D. nipponica* and *D. zingiberensis*, an expansion of the *CYP90*, *CYP94*, and *UGT73* gene families promoted dioscin biosynthesis and enhanced their dioscin content [5, 18].

*Dioscorea alata* is domesticated in Southeast Asia [14], and it is distributed in the tropical and subtropical regions, whereas *D. polystachya* thrives in temperate regions and covers the largest cultivation area for yam in China [33] with a broader ecological niche than *D. alata*. Previous studies have indicated that ≤33% of *Dioscorea* species display varying levels of ploidy, even within the same species [14]. One possible explanation is that asexual reproduction promotes polyploidy as a by-product of adaptive selection during prolonged periods of asexual reproduction, a strategy used by plants to protect themselves from extreme environmental damage [34]. Polyploidization results in expansion of gene families, and subsequent gene fractionation promotes gene sub-/neo-functionalization, ultimately enhancing the adaptation and genome plasticity of plant species.

### Dioscin levels in *D. polystachya* are regulated by ABA and DpbZIP12

The biosynthesis of plant saponins is modulated by phytohormones through the regulation of key enzymes or gene expression. ABA is a key regulator of secondary metabolism [35], enhancing flavonoid biosynthesis in tomato [36], promoting carotenoid accumulation in citrus (*Citrus sinensis*) fruits [37, 38], stimulating terpenoid biosynthesis in hemp (*Cannabis sativa*) [39], and regulating artemisinin biosynthesis in sweet wormwood (*Artemisia annua*) [40]. At present, few in-depth studies have explored the regulation of dioscin biosynthesis in plants. We showed that treating yams with ABA resulted in a significant rise in dioscin levels at 12 h post-treatment, followed by a drop at 24 h post-treatment (Fig. 6). ABA initially promotes the accumulation of secondary metabolites by upregulating the expression of genes encoding the respective biosynthetic enzymes, but prolonged ABA treatment may trigger feedback mechanisms. Long-term ABA treatment may induce the expression of negative regulators of ABA signaling (e.g. *Protein phosphatase 2C* [*PP2C*], *Sucrose nonfermenting 1-related protein kinase 2* [*SnRK2*]), accelerating the degradation of secondary metabolites to mitigate phytotoxicity from their excessive accumulation [41]. Concurrently, plants experiencing metabolite-induced stress may redirect energy resources toward essential maintenance such as membrane repair or osmotic homeostasis, potentially diverting substrates from secondary metabolism [42]. We thus suspect that a single ABA treatment elicits significant dioscin accumulation, whereas prolonged application may trigger a feedback mechanism, resulting in lower levels of secondary metabolites.

bZIP transcription factors are essential components of ABA signaling [43, 44]. In citrus, CsbZIP44 acts as a central regulator of ABA-induced carotenoid biosynthesis by binding to the promoters of carotenoid metabolism-related genes and activating their expression [37, 38]. Similarly, overexpression of *AabZIP1* in *A. annua* elevated artemisinin production via transcriptional activation of *Amorpha-4,11-diene synthase* (*ADS*) and *CYP71AV1* [45]. SmbZIP7 and SmbZIP20 in the perennial herb Danshen (*Salvia miltiorrhiza*) regulate tanshinone and artemisinin production [46]. OsbZIP79 in rice (*Oryza sativa*) regulates production of the diterpenoid antitoxin [47]. Two *Asparagus racemosus* bZIP transcription factors bind to the promoter region of the steroidal saponin biosynthesis gene *Squalene epoxidase* (*ArSQE*) [48]. Although bZIPs influence the accumulation of diverse secondary metabolites, their role in dioscin biosynthesis is uninvestigated, particularly within *Dioscorea* species. In this study, we identified an ABA-responsive bZIP transcription factor, DpbZIP12, that can bind to the *Dp7-DR* promoter to modulate dioscin biosynthesis (Figs 6 and 7, and Fig. S10). Among the DEGs in bulbils silenced for or overexpressing *DpbZIP12*, 18 DEGs assigned to the KEGG pathway ‘plant hormone signal transduction’ were specifically involved in ABA signal transduction, including genes encoding ABA receptors (PYL), protein phosphatase 2C (PP2C), kinases (SnRKs), ABA-responsive element binding factors (ABFs), and abscisic acid-insensitive 5 (ABI5) (Fig. S11C). Additional research is required to elucidate the regulatory mechanisms and interactions between ABA signal-related proteins and related transcription factors in the control of dioscin biosynthesis.

Of the ~600 *Dioscorea* species globally, only a limited number of cultivars are commercially utilized, representing untapped genetic potential and necessitating the exploration of accelerated biotechnological resources. Based on the released genomic data combined with the identification of dioscin-related genes, the key genes closely related to dioscin biosynthesis, transport, and accumulation can be further explored. For example, SNPs in the promoter of *CYP94D144* (identified in *D. zingiberensis*) are associated with variation in diosgenin content, suggesting its utility as a molecular breeding target [49]. Upregulating *CYP94D144* expression, which could be achieved via clustered regularly interspaced short palindromic repeats (CRISPR)/CRISPR-associated nuclease 9 (Cas9)-mediated gene editing, should enhance dioscin production and expedite breeding progress. In this study, we demonstrated that the DpbZIP12–*Dp7-DR* module regulates dioscin content, providing an excellent target for the study of dioscin biosynthesis and the genetic engineering of varieties with high dioscin content.

In conclusion, we assembled a haplotype-resolved chromosome-level genome from autotetraploid *D. polystachya*. In addition to the *tau* WGD event shared by monocotyledonous species and the *delta* event WGD associated with *Dioscorea*, *D. polystachya* experienced its own WGD event about 1.42 Mya, giving rise to its autotetraploid genome. Notably, dioscin biosynthetic pathway genes were generally over-retained compared to the diploid species *D. alata* (98.4% vs. 91.3%). We also demonstrated that *Dp7-DR* is involved in dioscin biosynthesis and identified a previously unknown regulator of dioscin biosynthesis, DpbZIP12, which stimulates dioscin production in yam tubers (Fig. 7I).

## Materials and methods

### Plant materials and treatment

Chinese yam (*D. polystachya* var. ‘Ruichang yam’) plants were grown at the experimental base of Jiangxi Agricultural University in Nanchang (28°45′53′′N, 115°49′41′′E), Jiangxi Province, China. Young leaves were collected for whole-genome sequencing, and roots, young stems, and tubers were sampled for RNA-seq analysis. The leaves of *D. polystachya* plants were sprayed with 100 μM abscisic acid (ABA), and samples were randomly collected at 0, 12, and 24 h after treatment, immediately flash frozen in liquid nitrogen and stored at −80°C until use.

### Genome size estimation

Illumina short reads were preprocessed using SOAPnuke (v1.6.5) for quality control, followed by genome size estimation via GenomeScope across multiple *k*-mer values. Separately, fresh leaves of *D. polystachya* and soybean (*Glycine max*, internal control) plants were collected and processed, and their purified nuclei were stained with propidium iodide. Nuclear DNA content was quantified using a FACSCalibur flow cytometer [50].

### Genome assembly, scaffolding, and phasing separation of subgenomes

The tetraploid genome of *D. polystachya* was assembled using hifiasm (v0.19.3-r572) [12] with PacBio HiFi long reads and Hi-C data, where the ‘--n-hap 4’ parameter was specified to enable phasing into four haplotypes. This step yielded preliminary haplotype-resolved assemblies, each representing one of the four homologous haplotypes.

To refine these assemblies, Purge_Dups (v1.2.5) [51] was employed to remove heterozygous and redundant sequences in a haplotype-specific manner. Briefly, HiFi and short reads were mapped to the entire preliminary assembly using minimap2 (v.2.28). Subsequently, the ‘split_paf.py’ script was used to partition the alignment results into four haplotype-specific subsets based on the phasing outcome from hifiasm. The coverage information of the mapped reads was then utilized to identify and purge redundant regions corresponding to each haplotype. This refinement step produced four haplotypes with sizes consistent with the estimates from flow cytometry, and the retained sequences were further verified by alignment with the genome of a closely related species (*D. alata*) to ensure no valid coding regions were erroneously removed.

For scaffolding, Hi-C reads were aligned to the refined assembly using BWA-mem (v.0.7.17). The aligned reads were then partitioned into four haplotype-specific subsets based on their best alignment scores, with multimapped reads retained and assigned to the corresponding haplotypes using a probability model implemented in HiC-Pro (v3.1.0). The Allhic (v0.9.8) pipeline was then run to scaffold each of the four subgenomes separately. Specifically, each haplotype-specific Hi-C subset was used to construct 20 pseudochromosomes per haplotype via the combined workflow of Juicer (v1.5) [52], 3D-DNA (180922), and Juicebox (v1.11.08) [53].

The resulting Juicebox ‘assembly’ files for each haplotype were merged and grouped according to homologous relationships identified via Nucmer (v3.23) [54]. Manual curation was subsequently performed based on the integrated Hi-C contact map of all four haplotypes. Contigs erroneously assigned between haplotypes were corrected with Juicebox based on the principle that contigs within the same haplotype should exhibit stronger Hi-C interactions than those between haplotypes. In addition, interchromosomal contacts were examined to reorder homologous pseudochromosomes across haplotypes, ensuring that chromosomes within the same haplotype displayed stronger global interaction signals compared to those across haplotypes. Through this strategy, a final assembly was obtained consisting of 80 pseudochromosomes, with 20 chromosomes per haplotype.

### Genome annotation

Protein-coding genes were predicted through an integrative approach combining *ab initio* predictions from Genemark (v4.61_lic) [55] and AUGUSTUS (v3.3.3) [56] with homology-based evidence, including plant protein sequences from the nonredundant (Nr) [57], EuKaryotic Orthologous Groups (KOG) [58], Gene Ontology (GO) [59], Swiss-Prot [60], and TrEMBL [61] databases, as well as a *de novo* transcriptome assembly constructed from *D. polystachya* RNA-seq data using Trinity (v2.11.0) [62].

Repeat sequences in the *D. polystachya* genome were identified *de novo* using RepeatModeler [63] and annotated with RepeatMasker [64] utilizing the repeat library.

### Genome-wide synteny analysis among yam species

Adjacent syntenic gene pairs were employed to delineate large-scale colinear genomic blocks between subgenomes. Considering local structural variations or potential assembly errors, syntenic gene pairs occasionally lacked immediate adjacency to neighboring syntenic pairs in one or both subgenomes. Syntenic fragments were consolidated into a single colinear block if separated by <50 genes or <300 kb in either genome [65].

Syntenic orthologs were identified in the genomes of *D. polystachya* and other Dioscoreaceae species (*D. zingiberensis* and *D. alata*) using mSynOrths with default parameters [66]. Syntenic genes identified between the *D. alata* and *D. polystachya* genomes served as the foundation for determining the baseline gene retention rate, defined as the proportion of syntenic genes retained in both genomes relative to the total number of syntenic gene pairs, and the same analytical framework was applied to quantify gene retention within the dioscin biosynthetic pathway.

### Comparison of expression levels between paralogs

Expression levels were compared between paralogs using RNA-seq data from leaves and tubers of *D. polystachya* plants. Paralogous gene pairs of the two haplotypes Hap1 and Hap3 were analyzed. The TPM values for genes from the two sets of chromosomes were counted using featureCounts [67]. Paralogous pairs with TPM values <2 were excluded to focus on expressed genes. Subsequent transcript quantification in *D. polystachya* was performed via Salmon (v0.13.1) with default parameters, enabling comprehensive statistical evaluation of gene expression patterns across all subgenomes.

### Variation in TE distribution around genes

To assess TE density in the regions flanking genes in *D. polystachya*, a 100-bp sliding window (10-bp step size) was applied. Specifically, the nucleotides derived from TEs were counted across 5-kb regions upstream (5′) and downstream (3′) of each gene. The ratio of nucleotides from TEs to those not derived from a TE was calculated per 100-bp sliding window. Subsequently, the average TE ratio was determined for the *D. polystachya* genes and plotted to estimate the TE density in their flanking regions.

### *Ks* analysis

The coding sequences of paralogous or orthologous gene pairs were aligned using Synorths [66]. The synonymous nucleotide substitution rate per synonymous site (*Ks*) was then calculated for each pair through the Nei and Gojobori method using KaKs_calculator [68]. *Ks* values were computed for intraspecific comparisons (*D. polystachya*, *D. zingiberensis*, *D. alata*) and interspecific comparisons (*D. polystachya* vs. *D. zingiberensis*, *D. polystachya* vs. *D. alata*, *D. alata* vs. *D. zingiberensis*), as well between Hap1 and Hap3 in *D. polystachya*, employing the equation *Ks* = 2μ*T*, where μ denotes per year substitution rate and *T* displays divergence time [16].

### Analysis of genome evolution

To dissect the phylogenetic relationship between *D. polystachya* and related species, genomic data from 12 species (*D. alata*, *D. polystachya*, *D. rotundata*, *D. zingiberensis*, *Brachypodium distachyon*, *O. sativa*, *Ananas comosus*, *Musa acuminata*, *Phalaenopsis equestris*, *Asparagus officinalis*, *Colocasia esculenta*, and *A. thaliana*) were subjected to gene family clustering analysis. Orthologous groups conserved across these genomes were identified through Orthofinder (v2.5.4) [69]. An ultrametric phylogenetic tree was reconstructed with r8s (v.1.81) [70]. Divergence times for each node were estimated via the MCMCtree program within the PAML package [71]. Gene family expansion and contraction dynamics were assessed using Cafe5 [72] to evaluate evolutionary trajectories across lineages.

### Comparative analysis among homologous haplotypes and RNA-SEQ analysis

SNP and InDel variants were detected using Nucmer, with variant density quantified across genomic regions using a 300-kb sliding window. Heatmaps illustrating SNP and InDel distribution were generated to elucidate evolutionary relationships among six haplotype pairs (H1–H2, H1–H3, H1–H4, H2–H3, H2–H4, H3–H4) at chromosomal resolution. To validate the accuracy of phasing assignments, phylogenetic trees were reconstructed using SNPs between *D. polystachya* and the closely related species *D. rotundata*. IQ-TREE (v2.2.6) [73] was used to generate maximum-likelihood trees for each set of four homologous chromosomes.

RNA-seq analysis was performed on young stems, roots, tuber peels, and tuber flesh. Raw RNA-seq reads were trimmed and aligned with HISAT2 (v.2.2.1) [74] and analyzed for allele-specific expression using DESeq2, applying a false discovery rate (FDR) <0.05 and |log_2_(fold-change)| > 1 [75].

Expression patterns from each haplotype were categorized as either biallelic (both alleles not significant) or imbalanced. The latter group was divided into three categories according to previous methods [76]: monoallelic expression of Hap1, monoallelic expression of Hap3, and higher expression of one allele. A criterion was established to determine the classification: haplotype-specific expression patterns in stems, roots, tuber peels, and tuber flesh. Specifically, genes with TPM > 2 for both Hap1 and Hap3 alleles and less than a 2-fold difference between the two haplotypes were classified as having ‘biallelic expression’. Genes with TPM > 2 for both alleles but at least a 2-fold difference in expression levels between the two haplotypes were defined as showing ‘higher expression of one allele’. When only one allele (either Hap1 or Hap3) had TPM > 2 and the other ≤2, the gene was defined as showing ‘Hap1 dominant expression’ or ‘Hap3 dominant expression’, respectively. Calculation of fragments per kilobase of exon model per TPM was performed using StringTie v2.1.4 [77].

### Transcriptome datasets and clustering of gene expression profile analysis

Data filtering of transcriptome datasets of four tuber developmental stages at 150 (T1), 170 (T2), 190 (T3), and 210 (T4) days after planting was performed using SOAPnuke (v1.6.5), and clean reads were aligned using HISAT2 [74], assembled, and quantified using Bowtie2 (v2.4.5) [78]. Gene expression levels were normalized to TPM [79], and DEGs were identified using the DESeq2 package [75] with the criteria of a fold change >1.5 and an FDR-adjusted *P* value <.05. Gene ontology (GO) term and KEGG pathway enrichment of DEGs and heatmap generation were performed using the R package clusterProfiler [80].

### Gene isolation, subcellular localization, real-time quantitative polymerase chain reaction, and end-point RT-PCR

The full-length coding sequence of *Dp7-DR* was cloned from *D. polystachya* tubers using a pClone007 versatile simple vector kit (Tsingke Biotechnology, Beijing, China). The sequences of proteins related to 7-DR from other species were identified by BLASTP search using NCBI and are listed in Table S14. A phylogenetic tree was reconstructed using MEGA7, employing a maximum likelihood model [81]. The promoter sequence and important *cis*-acting elements of *Dp7-DR* were analyzed using PlantCARE (https://bioinformatics.psb.ugent.be/webtools/plantcare/html/).

For subcellular localization analysis, the coding sequence of full-length *Dp7-DR* (excluding the stop codon) was amplified and cloned into the pBWA(V)HS-GLosgfp vector (Table S16). The resulting recombinant plasmid and the empty vector were separately introduced into *Agrobacterium tumefaciens* strain GV3101. Positive colonies were cultured in LB medium and then resuspended in infiltration buffer (10 mM MES, 120 μM AS, 10 mM MgCl₂, pH 5.6) to an OD_600_ of 0.6. Bacterial suspensions carrying the recombinant or control vectors were mixed in a 1:1 (v/v) ratio with SPER-mCherry (an ER localization marker) [82]. GFP fluorescence signals were observed 3 days after infiltration using an LSM 880 confocal laser microscope (Carl Zeiss, Germany).

Total RNA from yam roots, stems, leaves, and tubers was extracted for qPCR analysis using a TaKaRa MiniBEST Plant RNA Extraction Kit (Beijing, China), followed by reverse transcription into first-strand cDNA with a PrimeScript RT Kit. Three biological replicates were used for each sample, and the 2^−ΔΔCt^ method was employed for analysis using *ACTIN* (DpolyH1c19G001005) as the reference gene [4]. Specific primers used for each gene are listed in Table S16.

### Virus-induced gene silencing and transient overexpression in yam bulbils

The Sol Genomics Network (SGN) VIGS Tool (https://vigs.solgenomics.net/) [83] was used to predict the specific sequences for gene silencing. Following amplification, sequences were inserted into a pTRV2 vector carrying tobacco rattle virus RNA 2. The resulting construct was introduced into *Agrobacterium* strain GV3101. VIGS was conducted in yam bulbils following a protocol similar to that used for gladiolus [82]. A mixture of two *Agrobacterium* cell suspensions each containing pTRV1 or pTRV2 (1:1, v/v) was infiltrated into yam bulbils. Negative controls included an *Agrobacterium* cell suspension carrying pTRV1 and the empty pTRV2 vector (TRV). Yam bulbils subjected to VIGS were collected for real-time quantitative polymerase chain reaction (RT-qPCR) analysis to evaluate the effectiveness of gene silencing.

The full-length coding sequence of candidate genes was amplified and cloned into the pSuper1300 vector to create overexpression constructs (Table S16). Bulbils of *D. polystachya* was infiltrated with an *Agrobacterium* suspension harboring the target plasmid or the empty vector as control in infiltration buffer, adjusted to a final OD_600_ of 0.8. Tissue samples were collected from the infiltration sites for subsequent metabolite quantification and gene expression analysis.

### Extraction of dioscin and cholesterol and content measurements

The contents of dioscin and cholesterol were measured using an HPLC system (Shimadzu Nexera LC-30A) coupled with a tandem mass spectrometer (MS/MS) (AB Sciex QTrap 4500). Briefly, 200 mg of samples were homogenized with 1.2 ml of isopropanol, followed by ultrasonic extraction for 10 min at 4°C and centrifugation for 10 min. The resulting supernatant was collected and filtered using a 0.22-μm microporous filter membrane for cholesterol measurement. For dioscin measurements, the supernatant was concentrated to dryness at 4°C, resuspended in 70% (v/v) methanol, and filtered. Samples were separated using a Waters Acquity UPLC BEH C_18_ column (100 mm × 2.1 mm × 1.8 μm) at 45°C. Data were collected in dynamic-multiple reaction monitoring mode, with specific transitions controlled at specific times based on the retention time of the analytes. Concentrations were determined using an external calibration curve of the corresponding standard compounds (Fig. S10).

### Yeast one-hybrid assay, dual-luciferase reporter assay, and electrophoretic mobility shift assay

In the Y1H assay, the full-length *DpbZIP12* coding sequence, obtained from cDNA derived from total RNA isolated from *D. polystachya* tubers, was cloned into the pGADT7 vector (Table S16). A promoter fragment of *Dp7-DR* (475 bp) was amplified from genomic DNA extracted from young *D. polystachya* leaves and cloned into the pHIS2 vector (Table S16). After cotransforming the yeast strain Y187 with the pHIS2-pro-*Dp7-DR* reporter plasmid and the pGADT7-*DpbZIP12* effector plasmid [84], positive yeast colonies were selected and plated onto synthetic defined (SD) medium lacking leucine and tryptophan (SD/−Leu/−Trp) for 3 days. The positive colonies were resuspended and spotted onto the SD/−Leu/−Trp medium and SD/−Leu/−Trp/−His medium containing 40 mM 3-amino-1,2,4-triazole.

For the dual-luciferase reporter assay, the full-length *DpbZIP12* coding sequence was cloned into the pGreenII 62-SK vector to generate the effector plasmid (Table S16), which was then introduced into *A. tumefaciens* strain GV3101. A *Dp7-DR* promoter fragment (475 bp) was cloned into the pGreenII 0800-LUC vector to create a reporter plasmid (Table S16), which was introduced into *Agrobacterium* strain GV3101. The empty vector served as the negative control. *Agrobacterium* cell suspensions, each harboring the effector or reporter construct, were mixed and co-infiltrated into the leaves of *N. benthamiana* plants. A dual-luciferase reporter assay was performed as described previously [84]. The activities of Firefly luciferase (LUC) and *Renilla* luciferase (REN) were quantified using a commercial assay system (Promega) and detected with a Synergy™ H1 hybrid multimode microplate reader (BioTek). Experiments were performed independently at least three times (*n* = 3). Promoter activity was evaluated using relative LUC/REN ratios.

For the EMSA, the full-length *DpbZIP12* coding sequence was cloned into the pDONR207 entry vector (Invitrogen) using the Gateway system, and then recombined into the pHMGWA expression vector. The *MBP-DpbZIP12* plasmid was introduced into *Escherichia coli* BL21 cells, and protein production was induced with 0.5 mM IPTG. EMSA was then conducted using a LightShift Chemiluminescent EMSA Kit (Pierce Biotechnology, Rockford, IL, USA) with biotin-labeled DNA probes (5′-TTGTGCACACGTGTCGTGACAGGTCTCACACGTGGCAAGT-3′). To test the specificity of binding, unlabeled competitor probes (10×, 20× and 50× excess) were added to some samples. The reaction mixtures were separated on a polyacrylamide gel and visualized using a ChemiDoc XRS system (Bio-Rad Laboratories, Hercules, CA, USA).

### Statistical analysis

Statistical analyses were carried out using a paired two-tailed Student’s *t*-test in SPSS version 19.0, with significance levels indicated as ^*^P < 0.05, ^**^P < 0.01, and ^***^P < 0.001 for comparisons between two groups. Figures were generated using Origin 8.0. Data are presented as mean values ± standard deviation.

## Supplementary Material

Web_Material_uhaf344

## Data Availability

The assembled genome sequences are available under accession code PRJCA034592 at the Genome Warehouse (GWH) of the China National Center for Bioinformation (CNCB-NGDC, https://ngdc.cncb.ac.cn/gwh/), and all transcriptome raw sequence read data were uploaded to the China National GeneBank DataBase (CNGBdb, https://db.cngb.org/) website with the accession number CNP0008466. For comparative analysis, the genome of *D. alata* was obtained from YamBase (https://yambase.org/), and the gene, protein, and annotation files for *D. rotundata* were sourced from http://genome-e.ibrc.or.jp/home/bioinformatics-team/yam.

## References

[1] Shan N, Wang PT, Zhu QL. et al. Comprehensive characterization of yam tuber nutrition and medicinal quality of *Dioscorea opposita* and *D. alata* from different geographic groups in China. J Integr Agric. 2020;19:2839–48

[2] Chen H, Hu Z, Liu D. et al. Composition and physicochemical properties of three Chinese yam (*Dioscorea opposita* Thunb.) starches: a comparison study. Molecules. 2019;24:2973 31426303 10.3390/molecules24162973 PMC6719080

[3] Liu Y, Li H, Fan Y. et al. Antioxidant and antitumor activities of the extracts from Chinese yam (*Dioscorea opposita* Thunb.) flesh and peel and the effective compounds. J Food Sci. 2016;81:H1553–64 27122252 10.1111/1750-3841.13322

[4] Wang P, Shan N, Ali A. et al. Comprehensive evaluation of functional components, biological activities, and minerals of yam species (*Dioscorea polystachya* and *D. alata*) from China. LWT-Food Sci Technol. 2022;168:113964

[5] Cheng J, Chen J, Liu X. et al. The origin and evolution of the diosgenin biosynthetic pathway in yam. Plant Commun. 2021;2:100197 10.1016/j.xplc.2020.100079 PMC7816074 33511341

[6] Li Y, Tan C, Li Z. et al. The genome of *Dioscorea zingiberensis* sheds light on the biosynthesis, origin and evolution of the medicinally important diosgenin saponins. Hortic Res. 2022;9:uhac165 36204203 10.1093/hr/uhac165 PMC9531337

[7] Sonawane , Pollier J, Panda S. et al. Plant cholesterol biosynthetic pathway overlaps with phytosterol metabolism. Nat Plants. 2016;3:16205 28005066 10.1038/nplants.2016.205

[8] Christ B, Xu C, Xu M. et al. Repeated evolution of cytochrome P450-mediated spiroketal steroid biosynthesis in plants. Nat Commun. 2019;10:3206 31324795 10.1038/s41467-019-11286-7 PMC6642093

[9] Nakayasu M, Kawasaki T, Lee HJ. et al. Identification of furostanol glycoside 26-*O*-β-glucosidase involved in steroidal saponin biosynthesis from *Dioscorea esculenta*. Plant Biotechnol. 2015;32:299–308

[10] Ye T, Song W, Zhang JJ. et al. Identification and functional characterization of DzS3GT, a cytoplasmic glycosyltransferase catalyzing biosynthesis of diosgenin 3-*O*-glucoside in *Dioscorea zingiberensis*. Plant Cell Tissue Org. 2017;129:399–410

[11] Zhou C, Yang Y, Tian J. et al. 22R- but not 22S-hydroxycholesterol is recruited for diosgenin biosynthesis. Plant J. 2022;109:940–51 34816537 10.1111/tpj.15604

[12] Cheng H, Concepcion GT, Feng X. et al. Haplotype-resolved *de novo* assembly using phased assembly graphs with hifiasm. Nat Methods. 2021;18:170–5 33526886 10.1038/s41592-020-01056-5 PMC7961889

[13] Yin X, Liu J, Kou C. et al. Deciphering the network of cholesterol biosynthesis in *Paris polyphylla* laid a base for efficient diosgenin production in plant chassis. Metab Eng. 2023;76:232–46 36849090 10.1016/j.ymben.2023.02.009

[14] Sugihara Y, Kudoh A, Oli MT. et al. Population genomics of yams: evolution and domestication of *Dioscorea* species. In: Rajora OP, ed. Population Genomics: Crop Plants. Springer International Publishing: Cham, 2021,837–64

[15] Sugihara Y, Darkwa K, Yaegashi H. et al. Genome analyses reveal the hybrid origin of the staple crop white Guinea yam (*Dioscorea rotundata*). Proc Natl Acad Sci U S A. 2020;117:31987–92 33268496 10.1073/pnas.2015830117 PMC7749330

[16] Bredeson JV, Lyons JB, Oniyinde IO. et al. Chromosome evolution and the genetic basis of agronomically important traits in greater yam. Nat Commun. 2022;13:2001 35422045 10.1038/s41467-022-29114-w PMC9010478

[17] Zhang Y, Wei Z, Yang C. et al. A telomere-to-telomere genome assembly for greater yam (*Dioscorea alata*). Plant Commun. 2025;6:101326 40181549 10.1016/j.xplc.2025.101326 PMC12281257

[18] Hu K, Feng Y, Li P. et al. Haplotype-resolved genome and population genomics provide insights into dioscin biosynthesis and evolutionary history of the medicinal species *Dioscorea nipponica*. Plant J. 2025;121:e17237 39935194 10.1111/tpj.17237

[19] Arnau G, Némorin A, Maledon E. et al. Revision of ploidy status of *Dioscorea alata* L. (Dioscoreaceae) by cytogenetic and microsatellite segregation analysis. Theor Appl Genet. 2009;118:1239–49 19253018 10.1007/s00122-009-0977-6

[20] Tamiru M, Natsume S, Takagi H. et al. Genome sequencing of the staple food crop white Guinea yam enables the development of a molecular marker for sex determination. BMC Biol. 2017;15:1–20 28927400 10.1186/s12915-017-0419-x PMC5604175

[21] Cao T, Wang S, Ali A. et al. Transcriptome and metabolome analysis reveals the potential mechanism of tuber dynamic development in yam (*Dioscorea polystachya* Turcz.). LWT-Food Sci Technol. 2023;181:114764

[22] Soltis PS, Soltis DE. The role of hybridization in plant speciation. Annu Rev Plant Biol. 2009;60:561–88 19575590 10.1146/annurev.arplant.043008.092039

[23] Hu N, Gong J, Zhang B. Characterization of the complete chloroplast genome of *Dioscorea polystachya* Turcz. Mitochondrial DNA Part B. 2021;6:1652–3 34104726 10.1080/23802359.2021.1927222 PMC8143605

[24] Osborn TC, Chris Pires J, Birchler JA. et al. Understanding mechanisms of novel gene expression in polyploids. Trends Genet. 2003;19:141–7 12615008 10.1016/s0168-9525(03)00015-5

[25] Sattler MC, Carvalho CR, Clarindo WR. The polyploidy and its key role in plant breeding. Planta. 2016;243:281–96 26715561 10.1007/s00425-015-2450-x

[26] Van de Peer Y, Mizrachi E, Marchal K. The evolutionary significance of polyploidy. Nat Rev Genet. 2017;18:411–24 28502977 10.1038/nrg.2017.26

[27] Jansky SH, Charkowski AO, Douches DS. et al. Reinventing potato as a diploid inbred line–based crop. Crop Sci. 2016;56:1412–22

[28] Li Z, Chen ZJ. Nonadditive gene expression and epigenetic changes in polyploid plants and crops. Adv Agron. 2022; **176**:179–208

[29] Batiru G, Lübberstedt T. Polyploidy in maize: from evolution to breeding. Theor Appl Genet. 2024;137:182 39001883 10.1007/s00122-024-04688-9

[30] Chao DY, Dilkes B, Luo H. et al. Polyploids exhibit higher potassium uptake and salinity tolerance in *Arabidopsis*. Science. 2013;341:658–9 23887874 10.1126/science.1240561 PMC4018534

[31] Morineau C, Bellec Y, Tellier F. et al. Selective gene dosage by CRISPR-Cas9 genome editing in hexaploid *Camelina sativa*. Plant Biotechnol J. 2017;15:729–39 27885771 10.1111/pbi.12671 PMC5425392

[32] Zhang X, Tang C, Jiang B. et al. Refining polyploid breeding in sweet potato through allele dosage enhancement. Nat Plants. 2025;11:36–48 39668213 10.1038/s41477-024-01873-y

[33] Fan D, Zhong H, Hu B. et al. Agro-ecological suitability assessment of Chinese medicinal yam under future climate change. Environ Geochem Health. 2020;42:987–1000 31617038 10.1007/s10653-019-00437-w PMC7188720

[34] Freeling M . Picking up the ball at the K/Pg boundary: the distribution of ancient polyploidies in the plant phylogenetic tree as a spandrel of asexuality with occasional sex. Plant Cell. 2017;29:202–6 28213362 10.1105/tpc.16.00836 PMC5354197

[35] Li D, Xu G, Ren G. et al. The application of ultra-high-performance liquid chromatography coupled with a LTQ-Orbitrap mass technique to reveal the dynamic accumulation of secondary metabolites in licorice under ABA stress. Molecules. 2017;22:2115 29053618 10.3390/molecules22101742 PMC6151399

[36] Wu Q, Bai J, Tao X. et al. Synergistic effect of abscisic acid and ethylene on color development in tomato (*Solanum lycopersicum* L.) fruit. Sci Hortic. 2018;235:169–80

[37] Sun Q, He Z, Feng D. et al. The abscisic acid-responsive transcriptional regulatory module CsERF110-CsERF53 orchestrates citrus fruit coloration. Plant Commun. 2024;5:101065 39164970 10.1016/j.xplc.2024.101065 PMC11589302

[38] Sun Q, He Z, Wei R. et al. The transcriptional regulatory module CsHB5-CsbZIP44 positively regulates abscisic acid-mediated carotenoid biosynthesis in citrus (*Citrus* spp.). Plant Biotechnol J. 2024;22:722–37 37915111 10.1111/pbi.14219 PMC10893943

[39] Mansouri H, Asrar Z. Effects of abscisic acid on content and biosynthesis of terpenoids in *Cannabis sativa* at vegetative stage. Biol Plant. 2012;56:153–6

[40] Yuan M, Shu G, Zhou J. et al. AabHLH113 integrates jasmonic acid and abscisic acid signaling to positively regulate artemisinin biosynthesis in *Artemisia annua*. New Phytol. 2023;237:885–99 36271612 10.1111/nph.18567

[41] Jia J, Luo Y, Wu Z. et al. OsJMJ718, a histone demethylase gene, positively regulates seed germination in rice. Plant J. 2024;118:191–202 38116956 10.1111/tpj.16600

[42] Zhang H, Zhao Y, Zhu JK. Thriving under stress: how plants balance growth and the stress response. Dev Cell. 2020;55:529–43 33290694 10.1016/j.devcel.2020.10.012

[43] Yamaguchi-Shinozaki K, Shinozaki K. Transcriptional regulatory networks in cellular responses and tolerance to dehydration and cold stresses. Annu Rev Plant Biol. 2006;57:781–803 16669782 10.1146/annurev.arplant.57.032905.105444

[44] Yoshida T, Mogami J, Yamaguchi-Shinozaki K. ABA-dependent and ABA-independent signaling in response to osmotic stress in plants. Curr Opin Plant Biol. 2014;21:133–9 25104049 10.1016/j.pbi.2014.07.009

[45] Zhang F, Fu X, Lv Z. et al. A basic leucine zipper transcription factor, AabZIP1, connects abscisic acid signaling with artemisinin biosynthesis in *Artemisia annua*. Mol Plant. 2015;8:163–75 25578280 10.1016/j.molp.2014.12.004

[46] Zhang Y, Xu Z, Ji A. et al. Genomic survey of bZIP transcription factor genes related to tanshinone biosynthesis in *Salvia miltiorrhiza*. Acta Pharm Sin B. 2018;8:295–305 29719790 10.1016/j.apsb.2017.09.002 PMC5925414

[47] Miyamoto K, Nishizawa Y, Minami E. et al. Overexpression of the bZIP transcription factor OsbZIP79 suppresses the production of diterpenoid phytoalexin in rice cells. J Plant Physiol. 2015;173:19–27 25462074 10.1016/j.jplph.2014.09.001

[48] Upadhyay S, Jeena GS, Kumar S. et al. *Asparagus racemosus* bZIP transcription factor-regulated squalene epoxidase (ArSQE) promotes germination and abiotic stress tolerance in transgenic tobacco. Plant Sci. 2020;290:110291 31779892 10.1016/j.plantsci.2019.110291

[49] Sun S, Li Y, Jia L. et al. Identification of genetic variants controlling diosgenin content in *Dioscorea zingiberensis* tuber by genome-wide association study. BMC Plant Biol. 2024;24:540 38872080 10.1186/s12870-024-05133-1 PMC11177481

[50] Chen H, Zeng Y, Yang Y. et al. Allele-aware chromosome-level genome assembly and efficient transgene-free genome editing for the autotetraploid cultivated alfalfa. Nat Commun. 2020;11:2494 32427850 10.1038/s41467-020-16338-x PMC7237683

[51] Guan D, McCarthy SA, Wood J. et al. Identifying and removing haplotypic duplication in primary genome assemblies. Bioinformatics. 2020;36:2896–8 31971576 10.1093/bioinformatics/btaa025 PMC7203741

[52] Durand NC, Shamim MS, Machol I. et al. Juicer provides a one-click system for analyzing loop-resolution Hi-C experiments. Cell Syst. 2016;3:95–8 27467249 10.1016/j.cels.2016.07.002 PMC5846465

[53] Durand NC, Robinson JT, Shamim MS. et al. Juicebox provides a visualization system for Hi-C contact maps with unlimited zoom. Cell Syst. 2016;3:99–101 27467250 10.1016/j.cels.2015.07.012 PMC5596920

[54] Kurtz S, Phillippy A, Delcher AL. et al. Versatile and open software for comparing large genomes. Genome Biol. 2004;5:R12 14759262 10.1186/gb-2004-5-2-r12 PMC395750

[55] Lomsadze A, Ter-Hovhannisyan V, Chernoff Y. Gene identification in novel eukaryotic genomes by self-training algorithm. Nucleic Acids Res. 2005;33:6494–506 16314312 10.1093/nar/gki937 PMC1298918

[56] Hoff KJ, Stanke M. Predicting genes in single genomes with AUGUSTUS. Curr Protoc Bioinformatics. 2019;65:e57 30466165 10.1002/cpbi.57

[57] Marchler-Bauer A, Lu S, Anderson JB. et al. CDD: a conserved domain database for the functional annotation of proteins. Nucleic Acids Res. 2010;39:D225–9 21109532 10.1093/nar/gkq1189 PMC3013737

[58] Koonin EV, Fedorova ND, Jackson JD. et al. A comprehensive evolutionary classification of proteins encoded in complete eukaryotic genomes. Genome Biol. 2004;5:1–28 10.1186/gb-2004-5-2-r7 PMC395751 14759257

[59] Dimmer EC, Huntley RP, Alam-Faruque Y. et al. The UniProt-GO annotation database in 2011. Nucleic Acids Res. 2012;40:D565–70 22123736 10.1093/nar/gkr1048 PMC3245010

[60] Bairoch A, Apweiler R. The SWISS-PROT protein sequence database and its supplement TrEMBL in 2000. Nucleic Acids Res. 2000;28:45–8 10592178 10.1093/nar/28.1.45 PMC102476

[61] Boeckmann B, Bairoch A, Apweiler R. et al. The SWISS-PROT protein knowledgebase and its supplement TrEMBL in 2003. Nucleic Acids Res. 2003;31:365–70 12520024 10.1093/nar/gkg095 PMC165542

[62] Haas BJ, Papanicolaou A, Yassour M. et al. *De novo* transcript sequence reconstruction from RNA-seq using the trinity platform for reference generation and analysis. Nat Protoc. 2013;8:1494–512 23845962 10.1038/nprot.2013.084 PMC3875132

[63] Flynn JM, Hubley R, Goubert C. et al. RepeatModeler2 for automated genomic discovery of transposable element families. Proc Natl Acad Sci U S A. 2020;117:9451–7 32300014 10.1073/pnas.1921046117 PMC7196820

[64] Tarailo-Graovac M, Chen N. Using RepeatMasker to identify repetitive elements in genomic sequences. Curr Protoc Bioinformatics. 2009;4:4.10.11–14.10.14 10.1002/0471250953.bi0410s25 19274634

[65] Zhang K, Yang Y, Zhang X. et al. The genome of *Orychophragmus violaceus* provides genomic insights into the evolution of Brassicaceae polyploidization and its distinct traits. Plant Commun. 2023;4:100431 36071668 10.1016/j.xplc.2022.100431 PMC10030322

[66] Cheng F, Wu J, Fang L. et al. Syntenic gene analysis between *Brassica rapa* and other Brassicaceae species. Front Plant Sci. 2012;3:30895 10.3389/fpls.2012.00198 PMC3430884 22969786

[67] Liao Y, Smyth GK, Shi W. featureCounts: an efficient general purpose program for assigning sequence reads to genomic features. Bioinformatics. 2014;30:923–30 24227677 10.1093/bioinformatics/btt656

[68] Zhang Z, Li J, Zhao XQ. et al. KaKs_Calculator: calculating Ka and Ks through model selection and model averaging. Genom Proteom Bioinform. 2006;4:259–63 10.1016/S1672-0229(07)60007-2 PMC5054075 17531802

[69] Emms DM, Kelly S. OrthoFinder: phylogenetic orthology inference for comparative genomics. Genome Biol. 2019;20:238 31727128 10.1186/s13059-019-1832-y PMC6857279

[70] Sanderson MJ . r8s: inferring absolute rates of molecular evolution and divergence times in the absence of a molecular clock. Bioinformatics. 2003;19:301–2 12538260 10.1093/bioinformatics/19.2.301

[71] Yang Z . PAML: a program package for phylogenetic analysis by maximum likelihood. Comput Appl Biosci. 1997;13:555–6 9367129 10.1093/bioinformatics/13.5.555

[72] Mendes FK, Vanderpool D, Fulton B. et al. CAFE 5 models variation in evolutionary rates among gene families. Bioinformatics. 2021;36:5516–8 33325502 10.1093/bioinformatics/btaa1022

[73] Chernomor O, von Haeseler A, Minh BQ. Terrace aware data structure for phylogenomic inference from supermatrices. Syst Biol. 2016;65:997–1008 27121966 10.1093/sysbio/syw037 PMC5066062

[74] Kim D, Langmead B, Salzberg SL. HISAT: a fast spliced aligner with low memory requirements. Nat Methods. 2015;12:357–60 25751142 10.1038/nmeth.3317 PMC4655817

[75] Anders S, Huber W. Differential expression analysis for sequence count data. Genome Biol. 2010;11:R106 20979621 10.1186/gb-2010-11-10-r106 PMC3218662

[76] Zhang S, Yu Z, Sun L. et al. T2T reference genome assembly and genome-wide association study reveal the genetic basis of Chinese bayberry fruit quality. Hortic Res. 2024;11:uhae033 38495030 10.1093/hr/uhae033 PMC10940123

[77] Kovaka S, Zimin AV, Pertea GM. et al. Transcriptome assembly from long-read RNA-seq alignments with StringTie2. Genome Biol. 2019;20:1–13 31842956 10.1186/s13059-019-1910-1 PMC6912988

[78] Langmead B, Salzberg S. Fast gapped-read alignment with Bowtie 2. Nat Methods. 2012;9:357–9 22388286 10.1038/nmeth.1923 PMC3322381

[79] Mortazavi A, Williams BA, McCue K. et al. Mapping and quantifying mammalian transcriptomes by RNA-seq. Nat Methods. 2008;5:621–8 18516045 10.1038/nmeth.1226 PMC13303166

[80] Tarazona S, García-Alcalde F, Dopazo J. et al. Differential expression in RNA-seq: a matter of depth. Genome Res. 2011;21:2213–23 21903743 10.1101/gr.124321.111 PMC3227109

[81] Kumar S, Stecher G, Tamura K. MEGA7: molecular evolutionary genetics analysis version 7.0 for bigger datasets. Mol Biol Evol. 2016;33:1870–4 27004904 10.1093/molbev/msw054 PMC8210823

[82] Yuan Y, Ren S, Liu X. et al. SlWRKY35 positively regulates carotenoid biosynthesis by activating the MEP pathway in tomato fruit. New Phytol. 2022;234:164–78 35048386 10.1111/nph.17977

[83] Fernandez-Pozo N, Rosli HG, Martin GB. et al. The SGN VIGS tool: user-friendly software to design virus-induced gene silencing (VIGS) constructs for functional genomics. Mol Plant. 2015;8:486–8 25667001 10.1016/j.molp.2014.11.024

[84] An JP, Wang XF, Zhang XW. et al. Apple B-box protein BBX37 regulates jasmonic acid mediated cold tolerance through the JAZ-BBX37-ICE1-CBF pathway and undergoes MIEL1-mediated ubiquitination and degradation. New Phytol. 2021;229:2707–29 33119890 10.1111/nph.17050

