# Supplementary material for: A haplotype-resolved chromosome-level genome assembly of autotetraploid Chinese yam (Dioscorea polystachya) elucidates dioscin biosynthesis and regulation
Source: Hortic Res. 2025 Dec 11;13(3):uhaf344. doi: 10.1093/hr/uhaf344 (PMC12977164; doi:10.1093/hr/uhaf344)
Supplement: Web_Material_uhaf344 [file web_material_uhaf344.zip › 2025.11.16 Supplemental Figures+Tables(1).pdf]

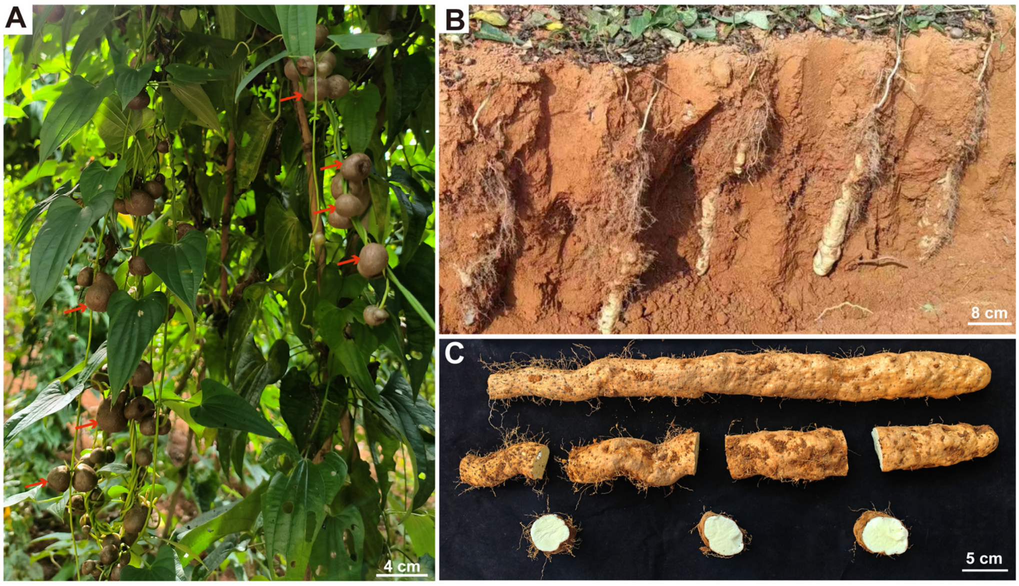

Supplemental Figure 1. Phenotypic characterization of the Chinese yam (*Dioscorea polystachya*) variety 'Ruichang yam'. **(A)** Field cultivation of Ruichang yam in Jiangxi Province. The red arrows indicated yam bulbils, which are modified stems that grow in the axils of leaves. **(B)** Tubers of Ruichang yam underground. **(C)** Harvested Ruichang yam tubers and characteristic cross-section.

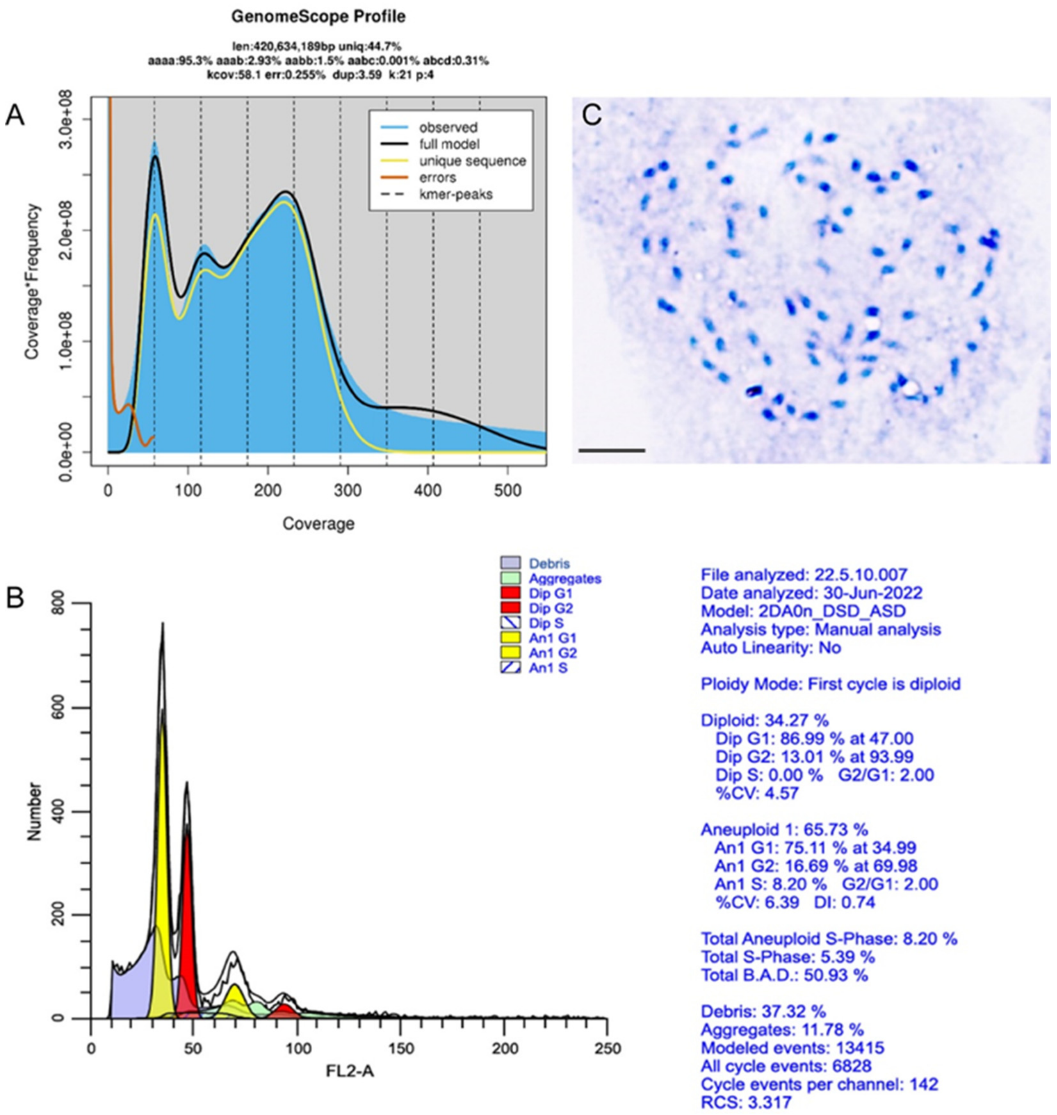

Supplemental Figure 2. Estimating the genome size of *D. polystachya*. (A) Genome size estimation via 21-mer frequency distribution, indicating a tetraploid structure with a haplotype genome size of approximately 420.63 Mb; heterozygosity rate 3.94%; repeat content 55.34%, revealing a highly heterozygous and repetitive genome. **(B)** *D. polystachya* genome size estimated by flow cytometry. Yellow peak denotes *D. polystachya*, while red peak represented *Glycine max* as internal reference. **(C)** Chromosome counts of *D. polystachya*. Mitotic metaphase chromosome spread of *D. polystachya*. In total, 15 mitotic metaphase or prometaphase chromosome spread images were prepared for counting. Eight of these spreads contained well-separated chromosomes, all of which supported a tetraploid karyotype (2n = 4x = 80). The s bar corresponds to 5 µm.

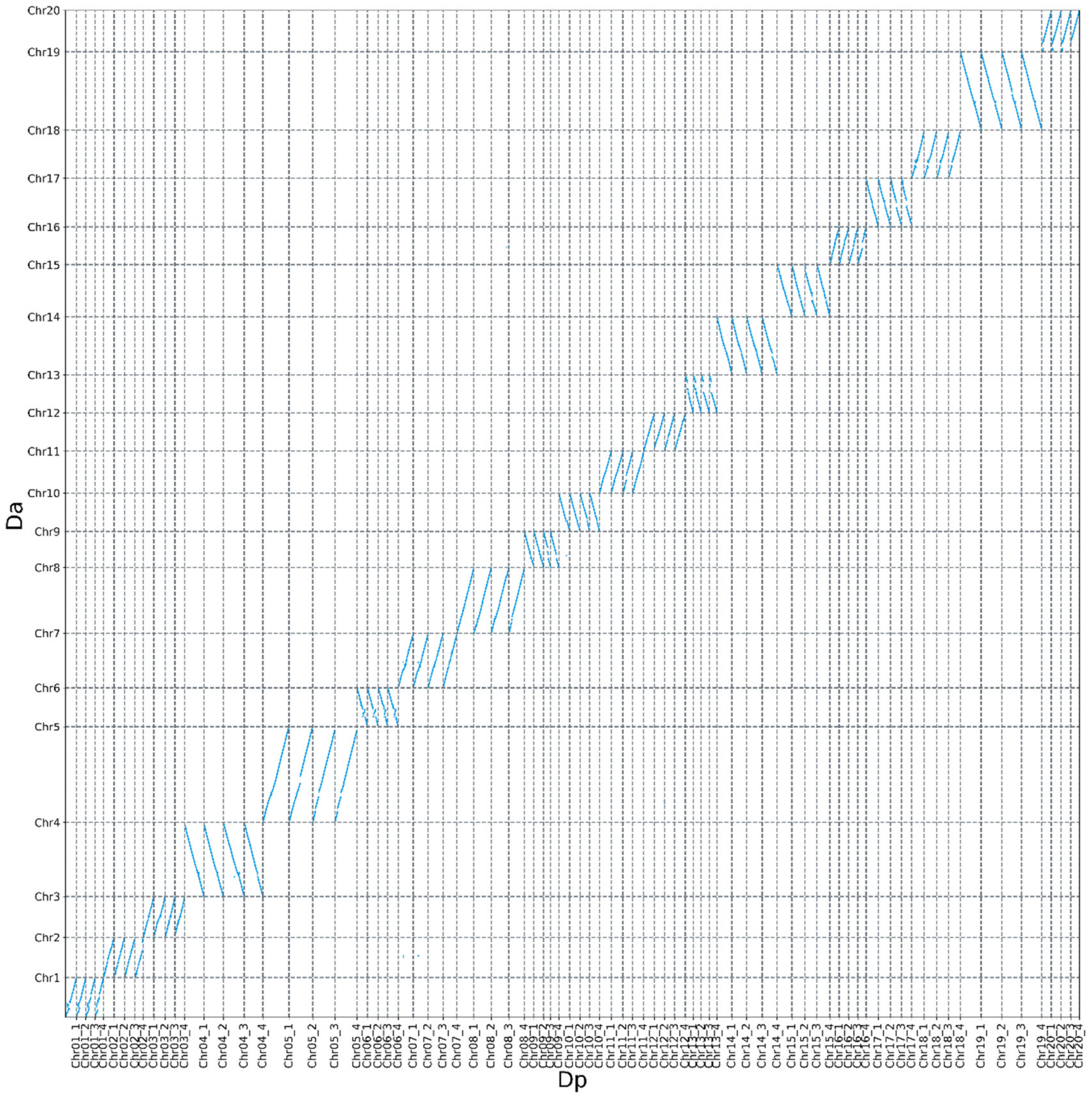

Supplemental Figure 3. Syntenic alignment of tetraploid *D. polystachya* homologous chromosomes to the corresponding single *D. alata* chromosome.

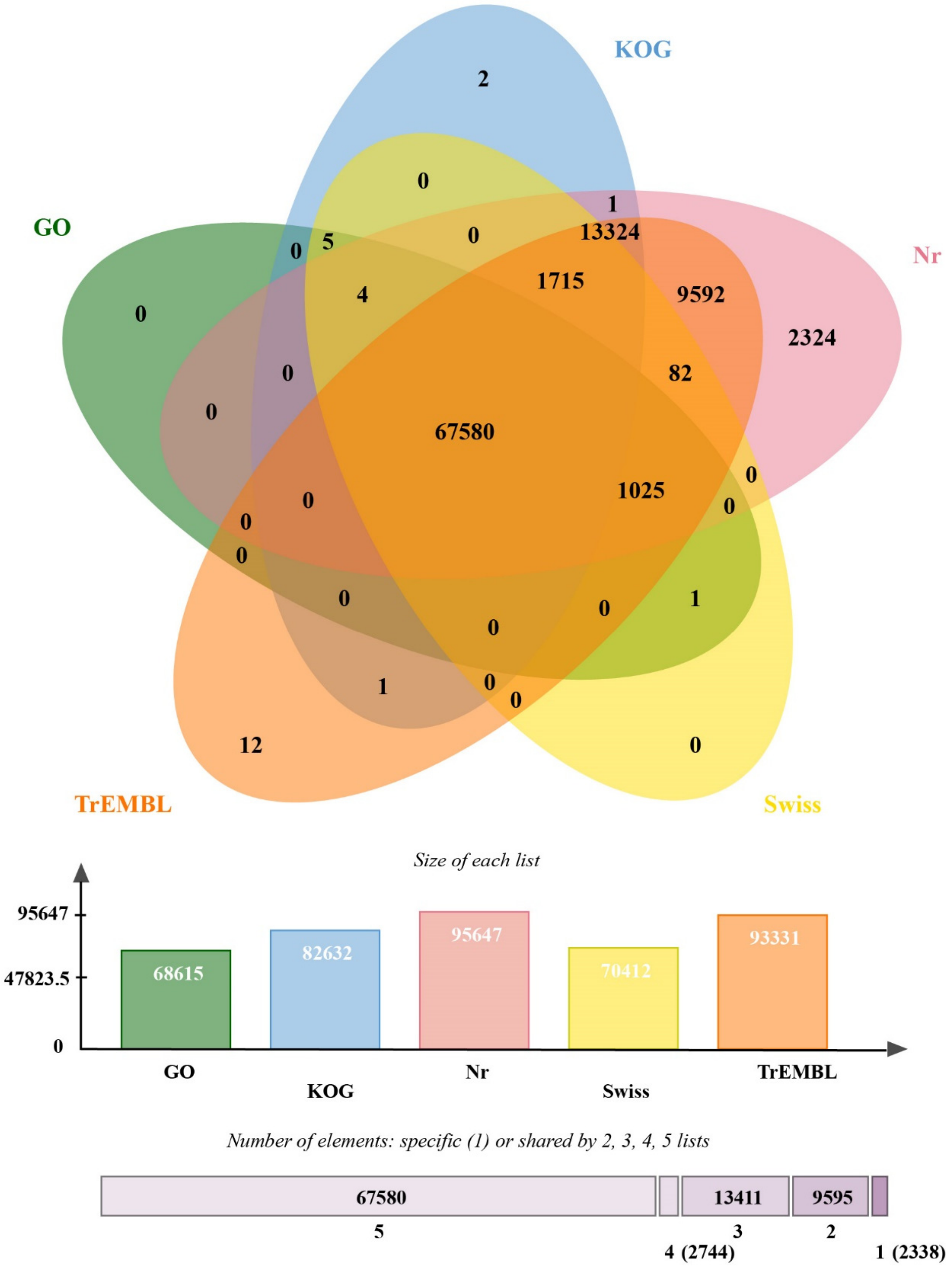

Supplemental Figure 4. Functional gene annotation of predicted *D. polystachya* genes by different databases. Swiss-Prot, http://www.uniprot.org/; KOG, Clusters of orthologous groups for eukaryotic complete genomes, https://www.ncbi.nlm.nih.gov/COG/; TrEMBL, http://www.uniprot.org; NR, Non-Redundant Protein Sequence Database, https://www.ncbi.nlm.nih.gov/protein; GO, Gene Ontology Resource.

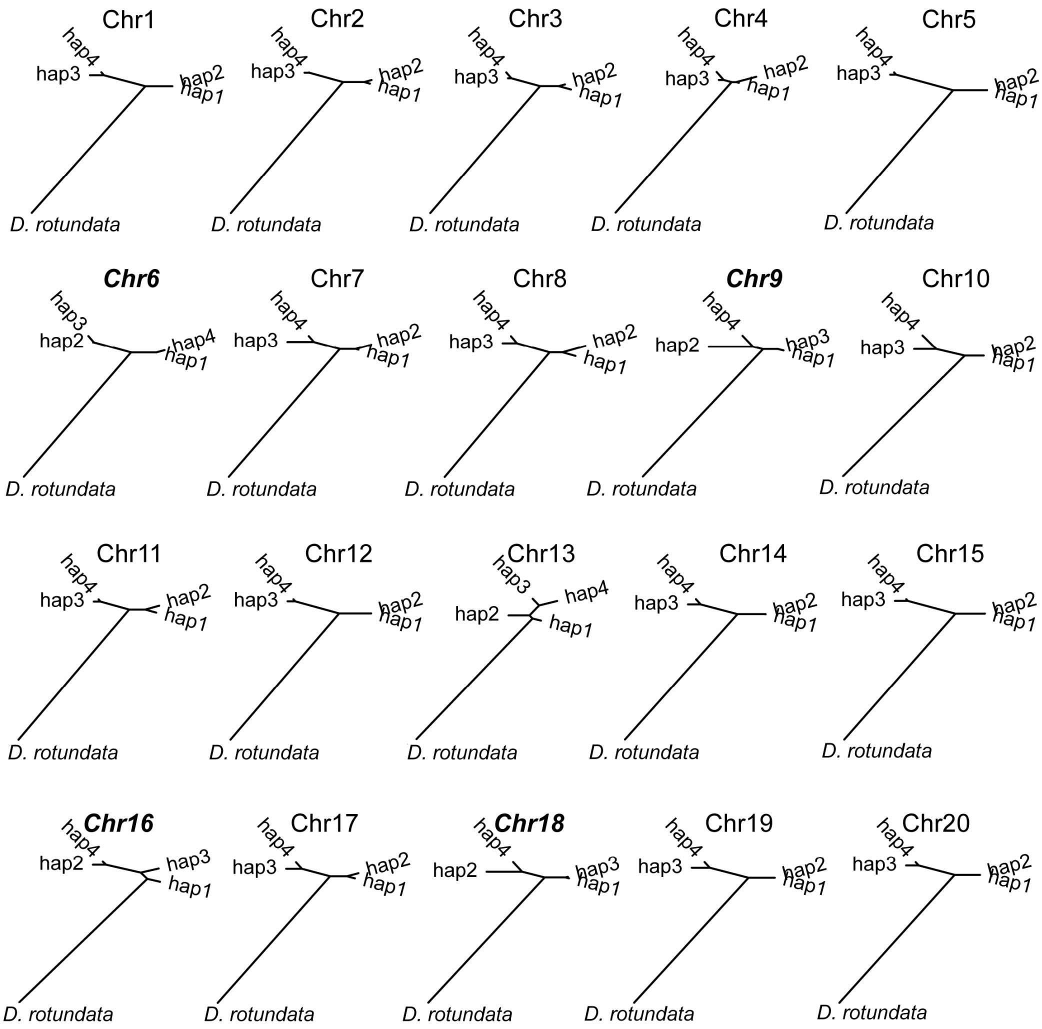

Supplemental Figure 5. Evolutionary relationships among the four *D. polystachya* haplotypes and *D. rotundata*.. Hap1 and Hap2 were phylogenetically close to each other on the majority of the 20 chromosomes (16/20), except for Chr6, Chr9, Chr16, Chr18, as were Hap3 and Hap4.

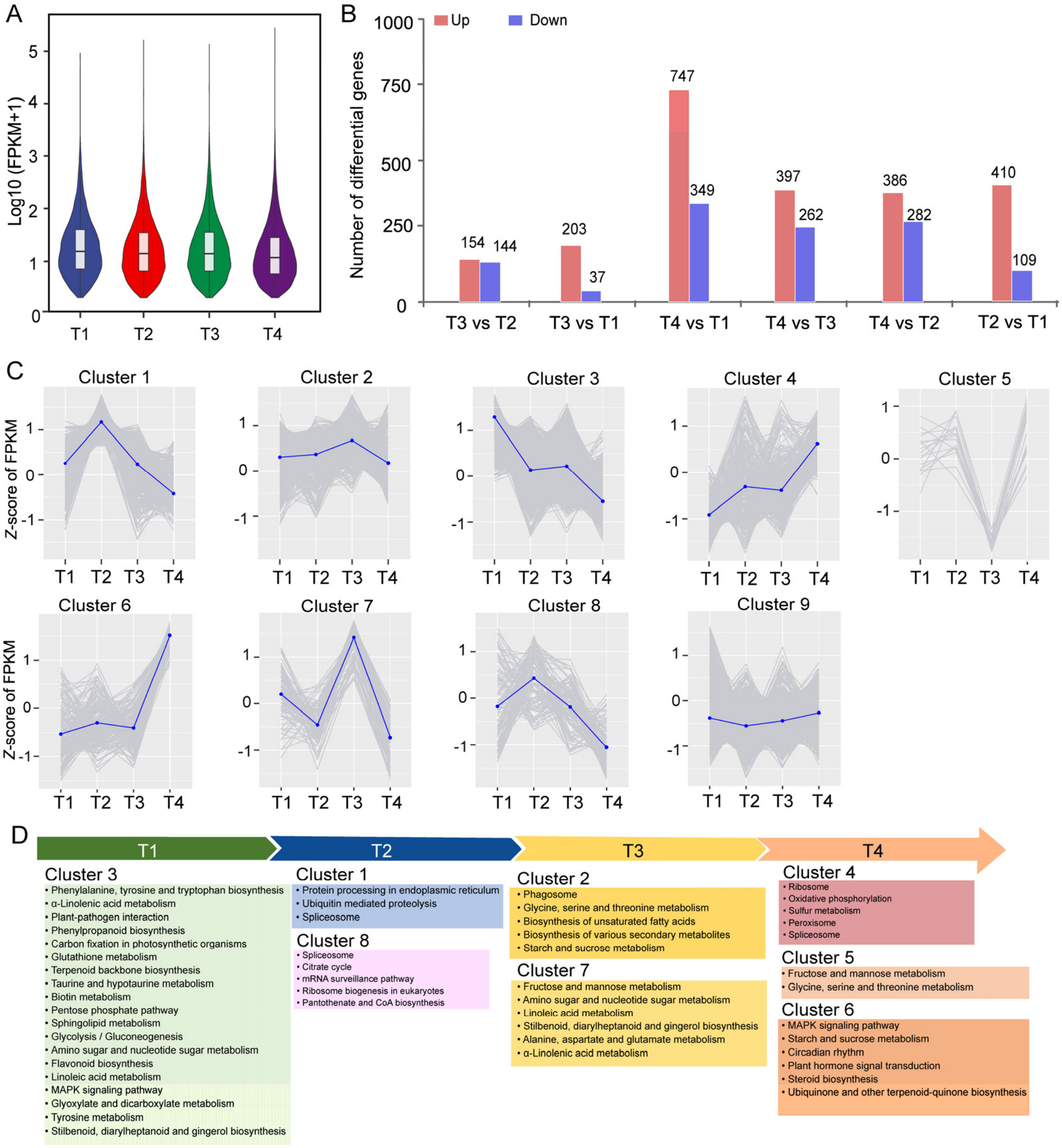

Supplemental Figure 6. Transcriptome analysis of *D. polystachya* tubers at different developmental stages. **(A)** The distribution of gene expression in *D. polystachya* tubers varies at the four stages. **(B)** The number of genes expressed differently in each comparison group. **(C)** Dynamics of gene expression during the *D. polystachya* tuber growth. *K*-means clustering grouped the expression profiles of the transcriptome into nine clusters. The x axis depicts four key developmental stages of yam tuber, and the y axis depicts the *Z*-score standardized gene expression levels per gene. **(D)** Kyoto Encyclopedia of Genes and Genomes (KEGG) terms associated with genes within the 9 clusters.

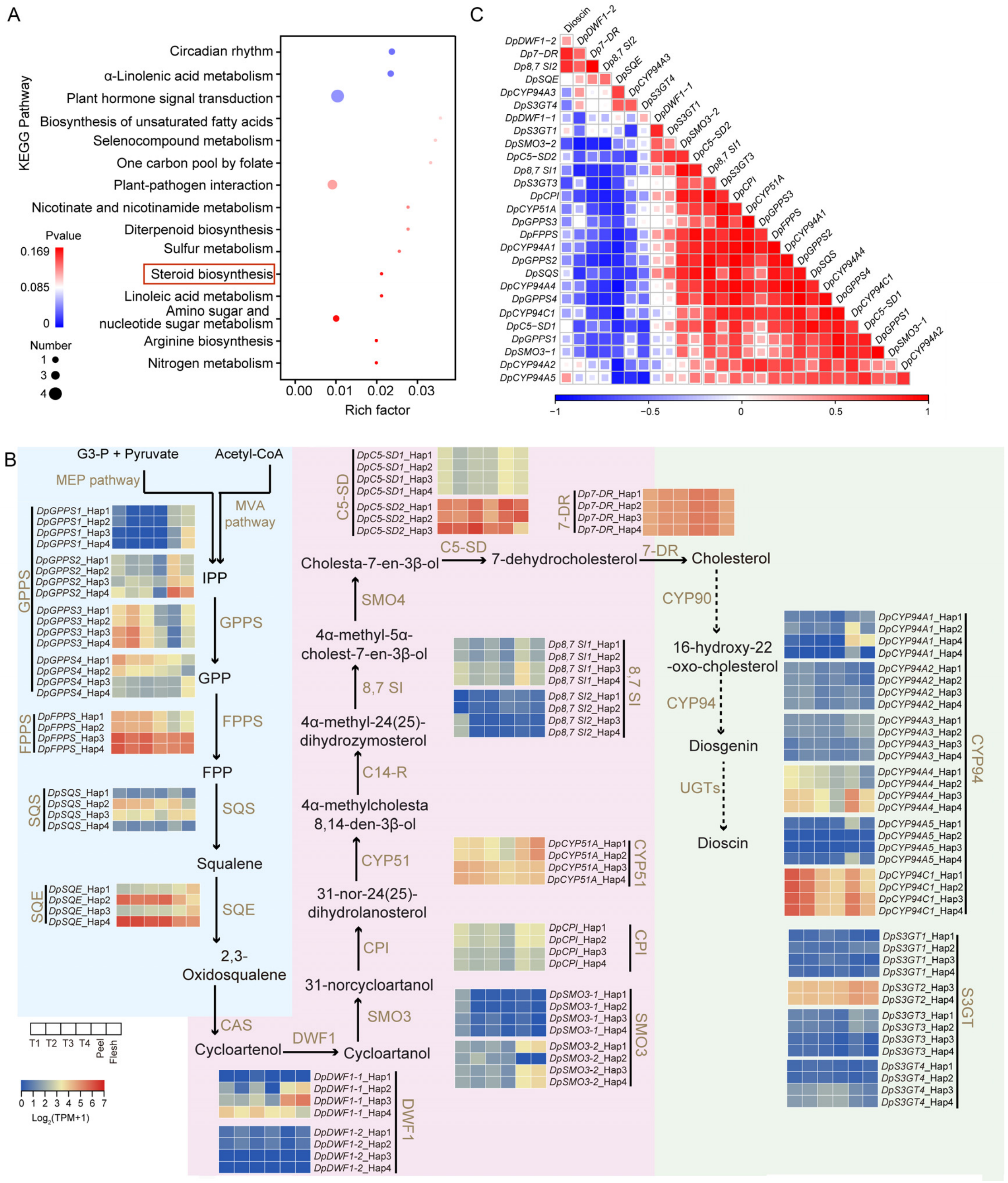

Supplemental Figure 7. KEGG annotation, heatmap, and correlation analysis of differentially expressed genes between selected groups. (A) KEGG annotation analysis on 100 differentially expressed genes that are shared between the T3 andT4 comparison groups, as well as between peel and flesh. The red box line represents *Dp7-DR* enrichment in this pathway. **(B)** The proposed biosynthesis pathway of dioscin in *D. polystachya*, with the expression levels of related genes shown in the accompanying heatmap. Expression values were scaled using $\log_2$(TKM + 1). Gene ID and gene names were indicated in Supplemental Table 13. Dashed arrows indicate multiple steps in the pathway. **(C)** Correlation analysis between expression of dioscin synthesis pathway genes in hap 1 and the dioscin content. MVA, mevalonate; MEP, methylerythritol 4-phosphate; CAS, cycloartenol synthase; SMO, C-4 sterol methyl oxidase; C14-R, sterol C-14 reductase; UGT, UDP-glycosyltransferases; DWF, δ-(24)-sterol reductase; CYP94, sterol 26-α-hydroxylase; S3GT, sterol

3-β-glucosyltransferase; CPI, cyclopropyl sterol isomerase; GPPS, geranyl pyrophosphate synthase; FPPS, farnesyl pyrophosphate synthase; SQS, squalene synthase; SQE, squalene epoxidase; CYP51, sterol C-14 demethylase; 8,7 SI, sterol 8;7 isomerase; C5-SD, sterol 5(6) desaturase; 7-DR, 7-dehydrocholesterol reductase.

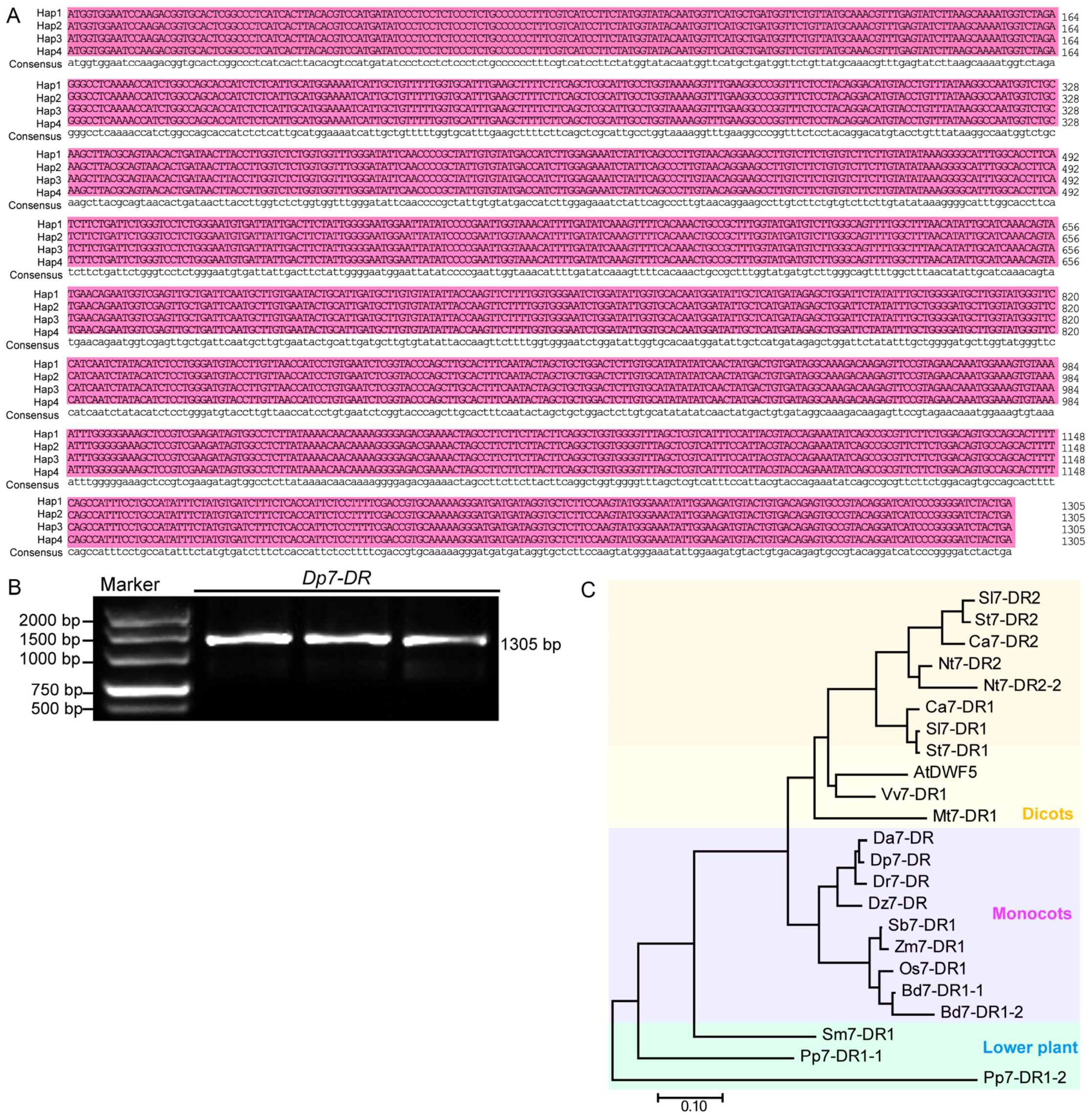

Supplemental Figure 8. Cloning and phylogenetic analysis of *Dp7-DR*. (A) Homologous alignment of four haplotypes of *Dp7-DR* gene sequences. The red box indicates 100% homology. **(B)** PCR amplification product of *Dp7-DR* with 1305 bp. **(C)** Phylogenetic tree of 7-DR. 7-DR sequences from dicots, including *Solanum lycopersicum* (*Sl*), *Solanum tuberosum* (*St*), *Capsicum annuum* (*Ca*), *Nicotiana tabacum* (*Nt*), *Arabidopsis thaliana* (*At*), *Vitis vinifera* (*Vv*), and *Medicago truncatula* (*Mt*), monocots, including *D. alata* (*Da*), *D. polystachya* (*Dp*), *D. rotundata* (*Dr*), *D. zingiberensis* (*Dz*), *Sorghum bicolor* (*Sb*), *Zea mays* (*Zm*), *Oryza sativa* (*Os*), *Brachypodium distachyon* (*Bd*), and lower plants, including *Selaginella moellendorffii* (*Sm*) and *Physcomitrella patens* (*Pp*) were employed for constructing phylogenetic tree.

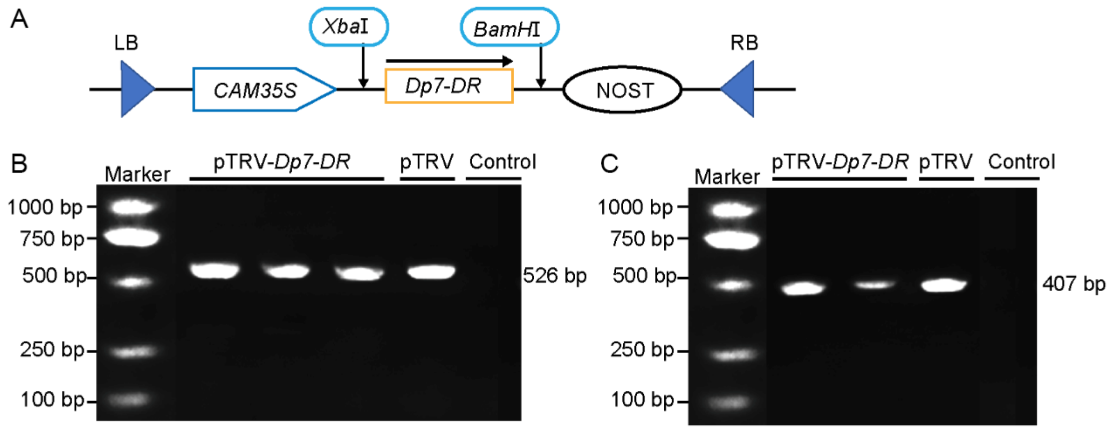

Supplemental Figure 9. Confirmation of the presence of the pTRV2-based VIGS vector in yam bulbils. (A) Schematic diagrams of vector plasmids used in gene silencing assays. **(B)-(C)** The accumulations of virus in yam bulbils of pTRV-*Dp7-DR* plants measured by using specific primes in TRV1 **(B)** and TRV2 **(C)**. TRV, Co-transformation of vector TRV1 with the empty vector TRV2. TRV-*Dp7-DR*, Co-transformation of vector TRV1 with vector TRV2- *Dp7-DR*.

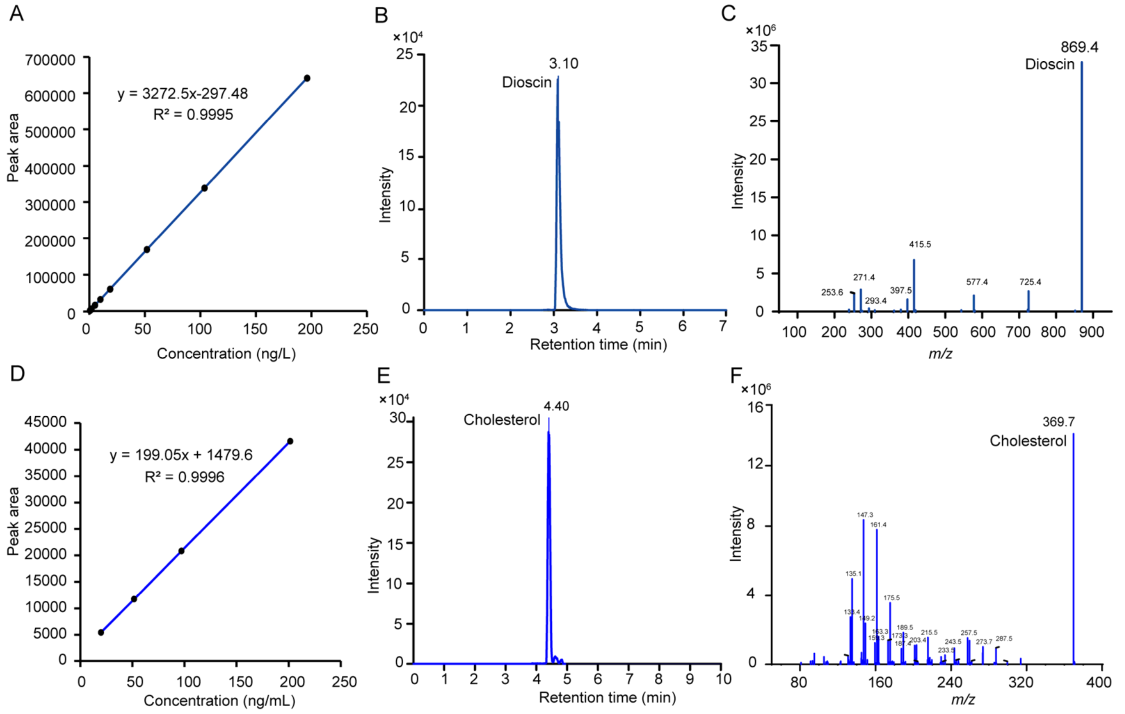

Supplemental Figure 10. HPLC–tandem mass spectrometry analysis of dioscin and cholesterol. The standard curve **(A, D)**, chromatogram **(B, E)** and mass spectrum **(C, F)** of dioscin **(A-C)** and cholesterol **(D-F)** were presented.

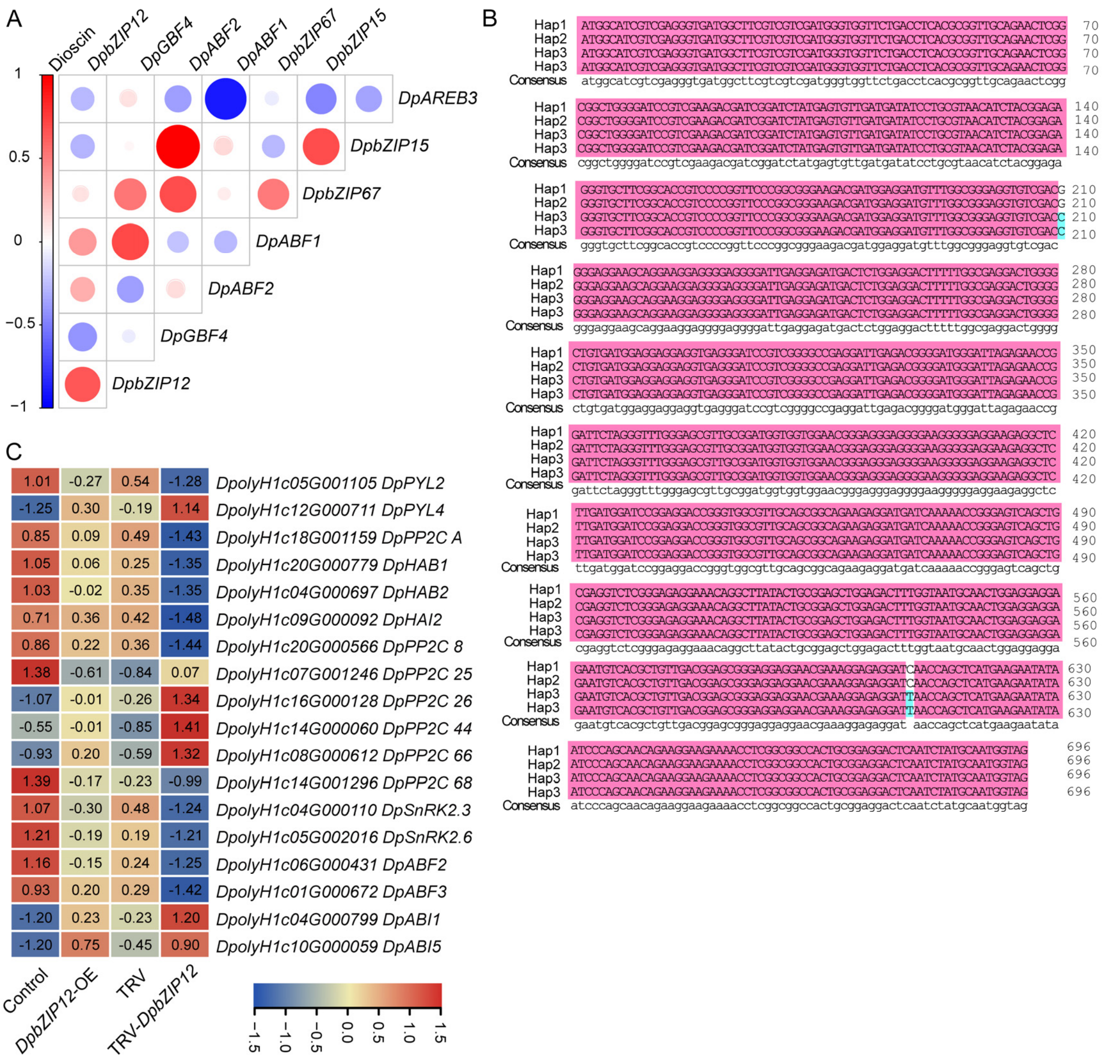

Supplemental Figure 11. Correlation and heatmap analysis of genes related to dioscin biosynthesis and ABA signaling. (A) Correlation between the TPM of bZIP transcription factor A subfamily genes and dioscin content. **(B)** Homologous alignment of four haplotypes of *DpbZIP12* gene sequences. The red box indicates 100% homology, and the bule represents base differences of four haplotypes of *DpbZIP* gene sequences. **(C)** Heatmap showing the DEGs related to ABA signal transduction pathway. The average TPM value for each DEG was resized to row *Z*-Score scale (from −1.5, the lowest expression to +1.5, the highest expression). PYL, ABA receptors; PP2C protein phosphatase 2C; HAB, hypersensitive to ABA; HAI, highly ABA-induced PP2C; SnRK, serine/threonine-protein kinase; ABF, ABA responsive element binding factors; ABI, abscisic acid-insensitive.

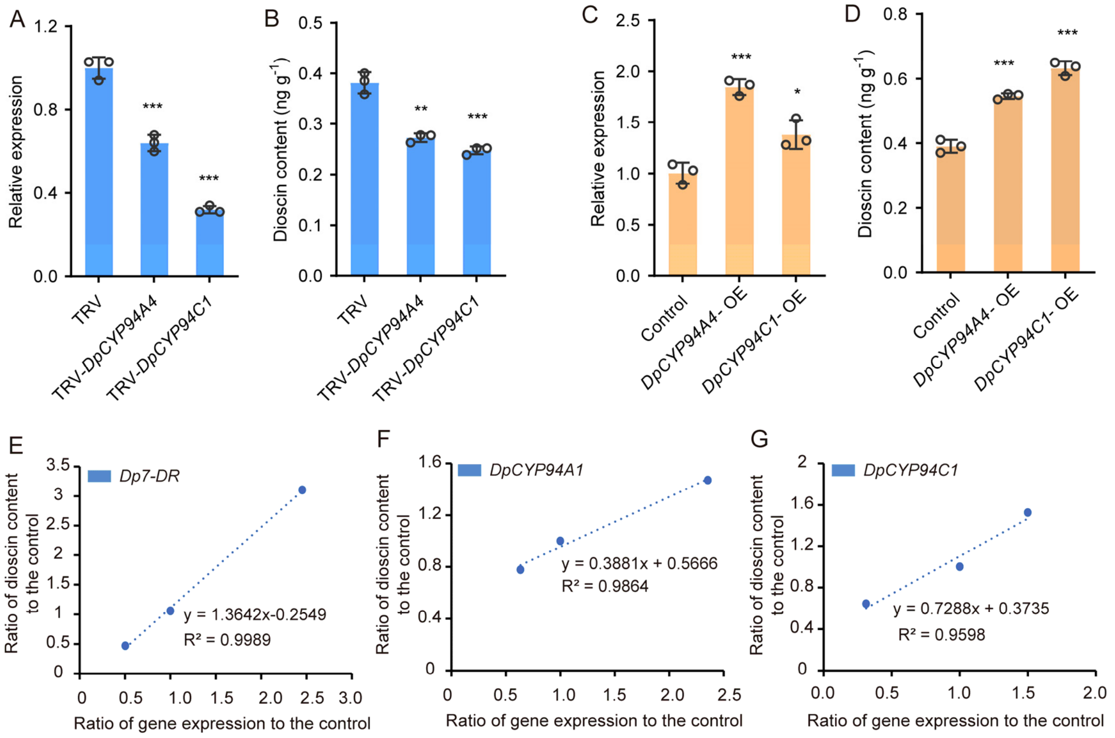

Supplemental Figure 12. Correlation between expression levels and dioscin content. (A)-(D) Expression levels **(A, C)** and dioscin contents **(B, D)** in silenced **(A-B)** or overexpressing **(C-D)** individual *DpCYP*s or *DpUGT*. Real-time quantitative PCR (RT-qPCR) was performed using gene-specific primers (Supplemental Table 15) on cDNA from specific tissues. *ACTIN* was used as a reference. Three biological replicates were used for each sample, and the $2^{-\Delta\Delta Ct}$ method was used to measure transcript levels. Values are shown as means ± standard deviation from three replicates (n = 3). TRV, co-infiltration of the pTRV1 vector with the empty vector pTRV2. *, $P < 0.05$; **, $P < 0.01$; ***, $P < 0.001$ (*t*-test). **(E)-(G)** Correlation between dioscin and gene expression levels in *Dp7-DR* **(E)**, *DpCYP94A1* **(F)**, and *CYP94C1* **(G)** TRV and overexpression bulbils. The gene expression levels and dioscin contents of the silenced and overexpressed materials were compared to the corresponding control ratios.

Supplemental Table 1. Results of *k*-mer frequency analysis to estimate *D. polystachya* genome size.

Kmer	nkmer	Used Base	Genome Size	Heter_rate	Repeat_rate	Err_rate
17	106,273,914,672	252,165,832,186	407,905,451	4.07245%	63.39%	0.207594%
19	104,889,610,760	252,065,749,098	417,037,063	4.15649%	57.57%	0.247201%
21	103,453,782,873	252,046,059,462	420,634,189	3.93962%	55.34%	0.254993%
23	101,975,588,424	252,044,381,598	422,762,868	3.76884%	53.88%	0.255657%
25	100,465,847,931	252,048,970,455	424,188,552	3.62547%	52.64%	0.253841%
27	98,936,036,931	252,057,723,651	425,582,464	3.48098%	51.52%	0.250377%
29	97,389,268,928	252,067,419,382	426,532,181	3.36552%	50.51%	0.24654%
31	95,827,503,513	252,080,602,240	427,845,081	3.24136%	49.59%	0.241323%

Supplemental Table 2. Estimation of *D. polystachya* genome size by flow cytometry.

Fluorescence intensity		Ratio	Size (G)	Average (G)	SD
Dioscorea polystachya	***Glycine max***				
35.17	47.59	0.74	1.63	1.64	0.11
35.4	50.64	0.7	1.54		
32.63	41.1	0.79	1.75		

Supplemental Table 3. Summary statistics of *D. polystachya* genome sequencing.

Type	Clean read	Clean data (Gb)	Reads length (bp)	GC rate (%)
DNBseq	509,822,524	76.47	150	39.54
Hi-C	1,787,480,822	268.12	150	38.94
PacBio HiFi	2,074,327	41.2	19,882	-

Supplemental Table 4. Summary statistics of final chromosome-level scaffolds.

Chromosome name	Length (bp)	Contig count	Count length	N count	N base
Chr01_1	18,988,425	5	18,986,425	4	2,000
Chr02_1	17,976,097	9	17,972,097	8	4,000
Chr03_1	15,124,225	4	15,122,725	3	1,500
Chr04_1	23,809,147	4	23,807,647	3	1,500
Chr05_1	30,257,148	7	30,254,148	6	3,000
Chr06_1	15,814,705	15	15,807,705	14	7,000
Chr07_1	23,524,521	5	23,522,521	4	2,000
Chr08_1	21,275,222	8	21,271,722	7	3,500
Chr09_1	16,010,387	5	16,008,387	4	2,000
Chr10_1	24,500,961	7	24,497,961	6	3,000
Chr11_1	17,028,664	3	17,027,664	2	1,000
Chr12_1	16,691,408	3	16,690,408	2	1,000
Chr13_1	15,516,838	3	15,515,838	2	1,000
Chr14_1	22,633,319	6	22,630,819	5	2,500
Chr15_1	20,595,367	3	20,594,367	2	1,000
Chr16_1	15,309,457	5	15,307,457	4	2,000
Chr17_1	18,263,258	5	18,261,258	4	2,000
Chr18_1	22,861,414	5	22,859,414	4	2,000
Chr19_1	24,569,244	11	24,564,244	10	5,000
Chr20_1	12,828,222	3	12,827,222	2	1,000
Chr01_2	13,813,569	6	13,811,069	5	2,500
Chr02_2	18,022,531	11	18,017,531	10	5,000
Chr03_2	20,040,189	6	20,037,689	5	2,500
Chr04_2	23,108,334	4	23,106,834	3	1,500
Chr05_2	26,541,869	6	26,539,369	5	2,500
Chr06_2	16,919,116	16	16,911,616	15	7,500
Chr07_2	22,963,447	4	22,961,947	3	1,500
Chr08_2	22,128,590	7	22,125,590	6	3,000
Chr09_2	15,642,673	4	15,641,173	3	1,500
Chr10_2	17,784,715	5	17,782,715	4	2,000
Chr11_2	17,193,083	3	17,192,083	2	1,000
Chr12_2	16,582,935	5	16,580,935	4	2,000
Chr13_2	15,634,913	2	15,634,413	1	500
Chr14_2	21,553,284	5	21,551,284	4	2,000
Chr15_2	18,256,837	7	18,253,837	6	3,000
Chr16_2	14,914,694	4	14,913,194	3	1,500
Chr17_2	19,054,772	6	19,052,272	5	2,500
Chr18_2	22,903,620	5	22,901,620	4	2,000
Chr19_2	23,869,671	6	23,867,171	5	2,500
Chr20_2	13,431,055	5	13,429,055	4	2,000
Chr01_3	13,598,136	5	13,596,136	4	2,000
Chr02_3	19,035,952	11	19,030,952	10	5,000

Chr03_3	14,842,749	7	14,839,749	6	3,000
Chr04_3	26,521,725	7	26,518,725	6	3,000
Chr05_3	21,864,948	5	21,862,948	4	2,000
Chr06_3	13,530,167	9	13,526,167	8	4,000
Chr07_3	23,476,302	4	23,474,802	3	1,500
Chr08_3	23,841,963	7	23,838,963	6	3,000
Chr09_3	8,764,667	3	8,763,667	2	1,000
Chr10_3	14,004,466	6	14,001,966	5	2,500
Chr11_3	11,764,013	3	11,763,013	2	1,000
Chr12_3	13,422,053	4	13,420,553	3	1,500
Chr13_3	13,800,806	2	13,800,306	1	500
Chr14_3	23,130,810	3	23,129,810	2	1,000
Chr15_3	14,513,447	4	14,511,947	3	1,500
Chr16_3	13,725,583	8	13,722,083	7	3,500
Chr17_3	14,361,863	5	14,359,863	4	2,000
Chr18_3	19,742,452	3	19,741,452	2	1,000
Chr19_3	22,594,168	11	22,589,168	10	5,000
Chr20_3	13,012,217	2	13,011,717	1	500
Chr01_4	11,674,714	9	11,670,714	8	4,000
Chr02_4	16,696,744	8	16,693,244	7	3,500
Chr03_4	14,967,221	6	14,964,721	5	2,500
Chr04_4	19,921,075	8	19,917,575	7	3,500
Chr05_4	22,317,973	4	22,316,473	3	1,500
Chr06_4	15,225,806	11	15,220,806	10	5,000
Chr07_4	21,158,453	2	21,157,953	1	500
Chr08_4	17,125,801	6	17,123,301	5	2,500
Chr09_4	11,067,917	4	11,066,417	3	1,500
Chr10_4	15,086,085	5	15,084,085	4	2,000
Chr11_4	16,631,065	5	16,629,065	4	2,000
Chr12_4	14,197,644	4	14,196,144	3	1,500
Chr13_4	12,819,661	2	12,819,161	1	500
Chr14_4	21,938,367	7	21,935,367	6	3,000
Chr15_4	16,752,468	6	16,749,968	5	2,500
Chr16_4	10,228,066	8	10,224,566	7	3,500
Chr17_4	13,396,708	6	13,394,208	5	2,500
Chr18_4	19,889,120	5	19,887,120	4	2,000
Chr19_4	23,098,143	13	23,092,143	12	6,000
Chr20_4	9,560,266	1	9,560,266	0	0
Total	1,451,206,205	462	1,437,048,710	382	191,000

Supplementary Table 5. Completeness analysis of genome assembly and annotation by Benchmarking Universal Single-Copy Orthologs (BUSCO)

Genomic features			Hap1	Hap2	Hap3	Hap4	*D.polystachya*.v1
Average of mRNA length	3439.71						
Average of exon number	5.25	Predicted gene/ annotation gene	25679	25369	24056	23595	
Average of exon length	252.24						
Assembly-BUSCO		Complete (%)	96.30	94.70	91.60	90.60	96.40
		Complete and single-copy (%)	91.40	92.20	89.50	89.10	90.90
		Complete and duplicated (%)	2.20	2.50	2.10	1.50	5.50
		Fragmented (%)	1.40	1.50	1.50	1.20	0.60
		Missing BUSCOs (%)	2.30	3.80	6.90	8.20	3.00
Annotation-BUSCO		Complete (%)	95.30	94.70	92.00	91.90	98.90
		Complete and single-copy (%)	87.70	84.80	0.84	82.60	97.70
		Complete and duplicated (%)	7.60	9.90	7.70	9.30	1.20
		Fragmented (%)	1.20	0.90	1.10	0.70	0.10
		Missing BUSCOs (%)	3.50	4.40	6.90	7.40	1.00

Supplemental Table 6. Variation information between haplotypes.

Comparison	SNP	Deletion	Insertion	Sum
Hap1-Hap2	1,439,482	150,733	151,429	1,741,644
Hap1-Hap3	3,842,353	364,434	360,260	4,567,047
Hap1-Hap4	4,125,062	390,498	386,223	4,901,783
Hap2-Hap3	4,155,552	393,205	388,837	4,937,594
Hap2-Hap4	4,019,936	383,290	379,552	4,782,778
Hap3-Hap4	1,868,333	179,962	178,065	2,226,360

Supplemental Table 7. Variation information (SNPs and Indels) between haplotypes in chromosome-wise comparison.

		Hap1	Hap2	Hap3	Hap4
Chr01	Hap1		65067	760	56343
	Hap2	65067		94280	81783
	Hap3	760	94280		91569
	Hap4	56343	81783	91569	
Chr02	Hap1		0	115705	110097
	Hap2	0		108608	108990
	Hap3	115705	108608		340
	Hap4	110097	108990	340	
Chr03	Hap1		38368	90405	114619
	Hap2	38368		94909	92329
	Hap3	90405	94909		60431
	Hap4	114619	92329	60431	
Chr04	Hap1		27179	139166	153978
	Hap2	27179		162486	127684
	Hap3	139166	162486		92392
	Hap4	153978	127684	92392	
Chr05	Hap1		114168	97558	97193
	Hap2	114168		90206	89736
	Hap3	97558	90206		37571
	Hap4	97193	89736	37571	
Chr06	Hap1		22055	131199	138515
	Hap2	22055		131361	133257
	Hap3	131199	131361		74635
	Hap4	138515	133257	74635	
Chr07	Hap1		750	108350	104234
	Hap2	750		108915	100082
	Hap3	108350	108915		16902
	Hap4	104234	100082	16902	
Chr08	Hap1		40873	92550	53530
	Hap2	40873		69473	54453
	Hap3	92550	69473		25373
	Hap4	53530	54453	25373	
Chr09	Hap1		19977	72809	102820
	Hap2	19977		72860	101434
	Hap3	72809	72860		6791
	Hap4	102820	101434	6791	
Chr10	Hap1		28	116396	125366
	Hap2	28		116115	125040
	Hap3	116396	116115		51910

	Hap4	125366	125040	51910	
Chr11	Hap1		8	67572	108885
	Hap2	8		68170	109410
	Hap3	67572	68170		63202
	Hap4	108885	109410	63202	
Chr12	Hap1		31000	79259	61443
	Hap2	31000		41773	85041
	Hap3	79259	41773		114269
	Hap4	61443	85041	114269	
Chr13	Hap1		1	71911	72282
	Hap2	1		72164	73447
	Hap3	71911	72164		40565
	Hap4	72282	73447	40565	
Chr14	Hap1		0	144944	145945
	Hap2	0		145223	146095
	Hap3	144944	145223		13
	Hap4	145945	146095	13	
Chr15	Hap1		135057	123033	136787
	Hap2	135057		90737	33957
	Hap3	123033	90737		84985
	Hap4	136787	33957	84985	
Chr16	Hap1		50	99148	77230
	Hap2	50		102848	80569
	Hap3	99148	102848		19744
	Hap4	77230	80569	19744	
Chr17	Hap1		98625	95451	58571
	Hap2	98625		105518	54301
	Hap3	95451	105518		82351
	Hap4	58571	54301	82351	
Chr18	Hap1		0	89186	79673
	Hap2	0		88593	78582
	Hap3	89186	88593		28684
	Hap4	79673	78582	28684	
Chr19	Hap1		66565	138601	144889
	Hap2	66565		143431	149934
	Hap3	138601	143431		70872
	Hap4	144889	149934	70872	
Chr20	Hap1		106189	2010	85906
	Hap2	106189		107153	76965
	Hap3	2010	107153		79366
	Hap4	85906	76965	79366	

Supplemental Table 8. Number of dominantly expressed genes between paralogous gene pairs from Hap1 and Hap3 in *D. polystachya*.

	hap1>hap3					hap1<hap3				
	(1，2]	(2，3]	(3，4]	(4，+]	Total	(1，2]	(2，3]	(3，4]	(4，+]	Total
Chr01	139	4	3	6	152	28	2	2	2	34
Chr02	82	13	4	4	103	102	25	5	3	135
Chr03	116	22	9	8	155	71	13	8	4	96
Chr04	274	37	13	22	346	278	66	23	17	384
Chr05	339	95	38	30	502	205	66	21	16	308
Chr06	97	21	4	5	127	126	35	13	14	188
Chr07	167	24	6	12	209	179	38	15	19	251
Chr08	187	51	21	16	275	170	46	19	17	252
Chr09	108	20	9	7	144	85	29	2	7	123
Chr10	106	26	6	7	145	123	29	5	12	169
Chr11	128	36	17	7	188	116	18	11	7	152
Chr12	97	19	0	5	121	90	24	8	5	127
Chr13	66	10	5	5	86	73	29	7	7	116
Chr14	153	35	12	7	207	159	77	25	21	282
Chr15	134	18	3	5	160	96	22	2	8	128
Chr16	91	16	3	4	114	117	25	11	7	160
Chr17	120	24	2	4	150	147	25	10	6	188
Chr18	146	32	13	15	206	128	26	10	5	169
Chr19	228	29	12	10	279	174	48	17	26	265
Chr20	116	14	2	2	134	36	6	1	3	46

Supplemental Table 9. Summary of allele-specific expression in four tissues of *D. polystachya*.

Classification of alleles		Stem		Root		Tuber peel		Tuber flesh	
		Number	Percentage (of total expressed alleles)	Number	Percentage (of total expressed alleles)	Number	Percentage (of total expressed alleles)	Number	Percentage (of total expressed alleles)
Biallelic expression		10,771	97.78%	10,826	97.60%	10,533	98.32%	10,449	98.52%
ASE		244	2.22%	266	2.40%	180	1.68%	157	1.48%
	Increased expression of one allele	193	1.75%	219	1.97%	142	1.33%	109	1.03%
	Monoallelic expression with Hap1	31	0.28%	14	0.13%	17	0.16%	19	0.18%
	Monoallelic expression with Hap3	20	0.18%	33	0.30%	21	0.20%	29	0.27%
Total expressed alleles		11,015	100.00%	11,092	100.00%	10,713	100.00%	10,606	100.00%

Supplemental Table 10. Colinearity analysis of orthologous genes involved in dioscin biosynthesis.

Gene description	Gene name	*D. polystachya* gene ID	*D. alata* gene ID	*D. zingiberensis* gene ID
Acetyl-CoA acetyltransferase	*ACAT*	DpolyH1c05G002401; DpolyH2c05G002161; DpolyH3c05G002097; DpolyH4c05G002102	Dioal.05G226200.1.v2.1	*D.zingiberensis*_LG01_G04492.t1
	ACAT	DpolyH1c16G000809; DpolyH2c16G000824; DpolyH3c16G000805; DpolyH4c16G000717	Dioal.16G084400.1.v2.1	*D.zingiberensis*_LG01_G05967.t1
3-Hydroxy-3-methylglutaryl-CoA Synthase	*HMGS*	DpolyH1c01G000747; DpolyH2c01G000606; DpolyH3c01G000587; DpolyH4c01G000502	Dioal.01G066300.1.v2.1	*D.zingiberensis*_LG09_G01630.t1
3-Hydroxy-3-methylglutaryl-CoA Reductase	*HMGR*	DpolyH1c05G002533; DpolyH2c05G002290; DpolyH3c16G000002; DpolyH4c16G000808	Dioal.05G238300.1.v2.1	*D.zingiberensis*_LG01_G04385.t1
	HMGR	DpolyH1c03G000920; DpolyH2c03G001012; DpolyH3c03G000875; DpolyH4c03G000860	Dioal.03G093200.1.v2.1	*D.zingiberensis*_LG01_G03402.t1
	HMGR	DpolyH1c16G000897; DpolyH2c16G000912; DpolyH3c16G000002; DpolyH4c16G000808	Dioal.16G094000.1.v2.1	*D.zingiberensis*_LG01_G06063.t1
Phosphate mevalonate kinase	*PMK*	DpolyH1c04G001224; DpolyH2c04G001204; DpolyH3c04G001323; DpolyH4c04G001222	Dioal.04G063700.1.v2.1	*D.zingiberensis*_LG05_G01258.t1
Mevalonate diphosphate decarboxylase	*MVD*	DpolyH1c10G000058; DpolyH2c10G000056; DpolyH3c10G000053; DpolyH4c10G000054	Dioal.10G090500.1.v2.1	*D.zingiberensis*_LG05_G02898.t1
1-Deoxy-D-xylulose 5-phosphate synthase	*DXS*	DpolyH1c07G000442; DpolyH2c07G000445; DpolyH3c07G000423; DpolyH4c07G000419	Dioal.07G042900.1.v2.1	*D.zingiberensis*_LG03_G03191.t1
	DXS	DpolyH2c15G000313; DpolyH3c15G000215; DpolyH4c15G000330	Dioal.15G097100.1.v2.1	*D.zingiberensis*_LG02_G02771.t1; *D.zingiberensis*_LG02_G02754.t1
	DXS	DpolyH1c08G001381; DpolyH2c08G001404; DpolyH3c08G001413; DpolyH4c08G001251	Dioal.08G137000.1.v2.1	*D.zingiberensis*_LG08_G01787.t1
	DXS	DpolyH1c05G000916; DpolyH2c05G000847; DpolyH3c05G000625; DpolyH4c05G000650	Dioal.05G078400.1.v2.1	*D.zingiberensis*_LG10_G00563.t1; *D.zingiberensis*_LG10_G00549.t1
1-Deoxy-D-xylulose 5-phosphate reductoisomerase	*DXR*	DpolyH1c17G000110; DpolyH2c17G000117; DpolyH3c17G000106; DpolyH4c17G000108	Dioal.17G108700.1.v2.1	*D.zingiberensis*_LG09_G00360.t1
2-C-methyl-D-erythritol 4-phosphate cytidyltransferase	*MCT*	DpolyH1c14G000584; DpolyH2c14G000597; DpolyH3c14G000655; DpolyH4c14G000637	Dioal.14G081600.1.v2.1	*D.zingiberensis*_LG06_G00704.t1
4-Diphosphocytidyl-2-C-methyl-D-erythritol kinase	*CMK*	DpolyH1c07G000783; DpolyH2c07G000769; DpolyH3c07G000725; DpolyH4c07G000666	Dioal.07G067200.1.v2.1	*D.zingiberensis*_LG03_G02425.t1
2-C-Methyl-D-erythritol 2,4-cyclodiphosphate synthase	*MDS*	DpolyH1c01G000707; DpolyH2c01G000564; DpolyH3c01G000546; DpolyH4c01G000460	Dioal.01G062300.1.v2.1	*D.zingiberensis*_LG09_G01580.t1
1-Hydroxy-2-methyl-2-butenyl-4-diphosphate synthase	*HDS*	DpolyH1c01G000011; DpolyH2c01G000010; DpolyH3c01G000011; DpolyH4c01G000011	Dioal.01G001200.1.v2.1	*D.zingiberensis*_LG09_G01283.t1
1-Hydroxy-2-methyl-2-butenyl-4-diphosphate reductase	*HDR*	DpolyH1c13G000759; DpolyH2c13G000751; DpolyH3c13G000768; DpolyH4c13G000727	Dioal.13G002200.1.v2.1	*D.zingiberensis*_LG02_G01732.t1
Isopentenyl diphosphate isomerase	*IDI*	DpolyH1c10G000605; DpolyH2c10G000615; DpolyH3c10G000637; DpolyH4c10G000607	Dioal.10G035500.1.v2.1	*D.zingiberensis*_LG05_G01408.t1
Geranyl pyrophosphate synthase	*GPPS*	DpolyH1c19G000867; DpolyH2c19G000872; DpolyH3c19G000859; DpolyH4c19G000854	Dioal.19G109100.2.v2.1	*D.zingiberensis*_LG04_G00072.t1
	GPPS	DpolyH1c13G000434; DpolyH2c13G000419; DpolyH3c13G000412; DpolyH4c13G000384	Dioal.13G034300.2.v2.1	*D.zingiberensis*_LG02_G02093.t1
	GPPS	DpolyH1c02G000261; DpolyH2c02G000288; DpolyH3c02G000259; DpolyH4c02G000279	Dioal.02G025500.1.v2.1	*D.zingiberensis*_LG07_G01438.t1

	GPPS	DpolyH1c16G000373; DpolyH2c16G000392; DpolyH3c16G000446; DpolyH4c16G000448	Dioal.16G041300.1.v2.1	*D.zingiberensis*_LG10_G00415.t1
Farnesyl pyrophosphate synthase	***FPPS***	DpolyH1c05G001621; DpolyH2c05G001382; DpolyH3c05G001308; DpolyH4c05G001323	Dioal.05G145800.1.v2.1	*D.zingiberensis*_LG01_G05312.t1
Squalene synthase	***SQS***	DpolyH1c08G001354; DpolyH2c08G001374; DpolyH3c08G001384; DpolyH4c08G001224	Dioal.08G134000.1.v2.1	*D.zingiberensis*_LG08_G01814.t1
Squalene epoxidase	***SQE***	DpolyH1c15G001096; DpolyH2c15G000968; DpolyH3c15G000833; DpolyH4c15G000963	Dioal.15G031400.1.v2.1	*D.zingiberensis*_LG02_G00279.t1
2,3-Oxidosqualene cyclase	***OSC***	DpolyH1c06G000362; DpolyH2c06G000362; DpolyH3c06G000366; DpolyH4c06G000359	Dioal.06G062600.1.v2.1	*D.zingiberensis*_LG05_G01110.t1
	OSC	DpolyH1c20G000187; DpolyH2c20G000226; DpolyH3c20G000209; DpolyH4c20G000068	Dioal.20G031500.1.v2.1	*D.zingiberensis*_LG01_G02098.t1
	OSC	DpolyH1c12G000940; DpolyH2c12G000894; DpolyH3c12G000852; DpolyH4c12G000963	Dioal.12G087100.1.v2.1	*D.zingiberensis*_LG10_G00656.t1; *D.zingiberensis*_LG10_G00624.t1
	OSC	DpolyH1c06G000804; DpolyH2c06G000826; DpolyH3c06G000723; DpolyH4c06G000788	Dioal.06G023500.1.v2.1	*D.zingiberensis*_LG02_G00664.t1
Sterol C-24 methyltransferase 1	***SMT1***	DpolyH1c19G000918; DpolyH2c19G000924; DpolyH3c19G000906; DpolyH4c19G000901	Dioal.19G104300.1.v2.1	*D.zingiberensis*_LG04_G00012.t1
	SMT1	DpolyH1c13G000351; DpolyH2c13G000334; DpolyH3c13G000304; DpolyH4c13G000270	Dioal.13G044900.1.v2.1	*D.zingiberensis*_LG02_G02226.t1
Sterol C-24 methyltransferase 2	***SMT2***	DpolyH1c14G001040; DpolyH2c14G001023; DpolyH3c14G001071; DpolyH4c14G000970	Dioal.14G045300.3.v2.1	*D.zingiberensis*_LG06_G01162.t1
C-4 Sterol methyl oxidase 1	***SMO1***	DpolyH1c01G000803; DpolyH2c01G000659; DpolyH3c01G000641; DpolyH4c01G000553	Dioal.01G072300.1.v2.1	*D.zingiberensis*_LG09_G01666.t1
	SMO1	DpolyH1c14G001311; DpolyH2c14G001307; DpolyH3c14G001367; DpolyH4c14G001302	Dioal.14G016600.1.v2.1	*D.zingiberensis*_LG06_G01631.t1
C-4 Sterol methyl oxidase 1	***SMO2***	DpolyH1c07G001419; DpolyH2c07G001407; DpolyH3c07G001402; DpolyH4c07G001317	Dioal.07G131500.1.v2.1	*D.zingiberensis*_LG03_G03040.t1
	SMO2	DpolyH1c09G000091; DpolyH2c09G000100; DpolyH3c09G000092; DpolyH4c09G000090	Dioal.09G080100.1.v2.1	*D.zingiberensis*_LG01_G00300.t1
Cyclopropylsterol isomerase	***CPI1***	DpolyH1c10G000330; DpolyH2c10G000326; DpolyH3c10G000324; DpolyH4c10G000338	Dioal.10G062900.1.v2.1	*D.zingiberensis*_LG05_G02544.t1
Sterol C-14 demethylase	***CYP51***	DpolyH1c10G001000; DpolyH2c10G000939; DpolyH3c10G000868; DpolyH4c10G000890	Dioal.10G006300.1.v2.1	*D.zingiberensis*_LG05_G03024.t1
Sterol 8;7 isomerase	***8,7 SI***	DpolyH1c05G001939; DpolyH2c05G001704; DpolyH3c05G001634; DpolyH4c05G001637	Dioal.05G178600.1.v2.1	*D.zingiberensis*_LG01_G04983.t1
	8,7 SI	DpolyH1c10G001031; DpolyH2c10G000971; DpolyH3c10G000899; DpolyH4c10G000921	Dioal.10G003100.1.v2.1	*D.zingiberensis*_LG05_G02990.t1
Sterol 5(6) Desaturase	***C5-SD***	DpolyH1c05G001490; DpolyH2c05G001246; DpolyH3c05G001164; DpolyH4c05G001186	Dioal.05G131100.1.v2.1	*D.zingiberensis*_LG01_G05456.t1
	C5-SD	DpolyH1c17G000292; DpolyH2c17G000301; DpolyH3c17G000291	Dioal.17G088000.1.v2.1	*D.zingiberensis*_LG09_G00195.t1
7-Dehydrocholesterol reductase	***7-DR***	DpolyH1c03G000046; DpolyH2c03G000045; DpolyH3c03G000049; DpolyH4c03G000003	Dioal.03G005000.1.v2.1	*D.zingiberensis*_LG01_G01434.t1
δ-(24)-Sterol reductase	***DWF1***	DpolyH1c01G000548; DpolyH2c01G000428; DpolyH3c01G000467; DpolyH4c01G000370	Dioal.01G047100.1.v2.1	*D.zingiberensis*_LG09_G01426.t1
	DWF1	DpolyH1c14G000255; DpolyH2c14G000257; DpolyH3c14G000277; DpolyH4c14G000277	Dioal.14G116900.1.v2.1	*D.zingiberensis*_LG06_G00299.t1; *D.zingiberensis*_LG06_G00290.t1
	DWF1	DpolyH1c11G000730; DpolyH2c11G000733; DpolyH3c11G000558; DpolyH4c11G000720	Dioal.11G065700.1.v2.1	*D.zingiberensis*_LG07_G00356.t1
Sterol 22-α-hydroxylase	***CYP90B***	DpolyH1c13G000658; DpolyH2c13G000652; DpolyH2c13G000661; DpolyH3c13G000669; DpolyH4c13G000633	Dioal.13G011000.1.v2.1	*D.zingiberensis*_LG02_G01820.t1; *D.zingiberensis*_LG02_G01826.t1
	CYP90B	DpolyH1c02G000111; DpolyH2c02G000118; DpolyH3c02G000111; DpolyH4c02G000120	Dioal.02G011100.1.v2.1	*D.zingiberensis*_LG07_G01292.t1

Sterol 26-α-hydroxylase	***CYP72A***	DpolyH1c20G000762; DpolyH2c20G000808; DpolyH3c20G000769; DpolyH4c20G000682	Dioal.20G088200.1.v2.1<br>Dioal.20G088300.1.v2.1	*D.zingiberensis*_LG01_G02198.t1;<br>*D.zingiberensis*_LG01_G02207.t1;<br>*D.zingiberensis*_LG01_G02214.t1
Sterol 26-α-hydroxylase	***CYP94***	DpolyH1c06G000585; DpolyH2c06G000596; DpolyH4c06G000648; DpolyH4c06G000654	Dioal.06G031300.1.v2.1	*D.zingiberensis*_LG02_G00783.t1;<br>*D.zingiberensis*_LG02_G00813.t1
	CYP94	DpolyH1c13G000664; DpolyH2c13G000656; DpolyH2c13G000662;<br>DpolyH3c13G000674; DpolyH4c13G000639	Dioal.13G010900.1.v2.1	*D.zingiberensis*_LG02_G01821.t1
	CYP94	DpolyH1c12G000519; DpolyH2c12G000479; DpolyH3c12G000453; DpolyH4c12G000535	Dioal.12G048000.1.v2.1	*D.zingiberensis*_LG03_G01478.t1
	CYP94	DpolyH1c07G000715; DpolyH2c07G000688; DpolyH3c07G000651; DpolyH4c07G000583	Dioal.07G060000.1.v2.1	*D.zingiberensis*_LG03_G02336.t1
	CYP94	DpolyH1c07G000801; DpolyH2c07G000793; DpolyH3c07G000767; DpolyH4c07G000704	Dioal.07G068900.1.v2.1	*D.zingiberensis*_LG03_G02433.t1
	CYP94	DpolyH1c10G000047; DpolyH2c10G000045; DpolyH3c10G000043; DpolyH4c10G000045	Dioal.10G091600.1.v2.1	*D.zingiberensis*_LG05_G02913.t1
	CYP94	DpolyH1c02G000112; DpolyH2c02G000119; DpolyH3c02G000112; DpolyH4c02G000121	Dioal.02G011200.1.v2.1	*D.zingiberensis*_LG07_G01291.t1
	CYP94	DpolyH1c08G000725; DpolyH2c08G000761; DpolyH3c08G000780; DpolyH4c08G000606	Dioal.08G073600.1.v2.1<br>Dioal.08G070000.1.v2.1	*D.zingiberensis*_LG08_G00905.t1
Sterol 3-β-glucosyltransferase	***S3GT***	DpolyH1c01G000154; DpolyH2c01G000152; DpolyH3c01G000155; DpolyH4c01G000145	Dioal.01G025300.1.v2.1	*D.zingiberensis*_LG09_G01145.t1
	S3GT	DpolyH3c02G000607; DpolyH4c02G000636	Dioal.02G060200.1.v2.1	*D.zingiberensis*_LG07_G00747.t1
	S3GT	DpolyH1c17G001003; DpolyH2c17G001037; DpolyH3c17G000912; DpolyH4c17G000831	Dioal.17G018600.3.v2.1	*D.zingiberensis*_LG01_G04323.t1
	S3GT	DpolyH1c14G000769; DpolyH2c14G000797; DpolyH3c14G000842; DpolyH4c14G000811	Dioal.14G066800.1.v2.1	*D.zingiberensis*_LG06_G00901.t1;<br>*D.zingiberensis*_LG06_G00935.t1;<br>*D.zingiberensis*_LG06_G00926.t1;<br>*D.zingiberensis*_LG06_G00918.t1

Supplemental Table 11. **Copy number of genes in the dioscin biosynthetic pathway and their expression levels in tubers of *D. polystachya*.**

Gene name	Gene ID	Copy Number Variation	TPM	Copy Gene	Copy Gene TPM
ACAT	DpolyH1c05G002401	Single Copy	3.9145	\	\
	DpolyH2c05G002161	Single Copy	0.0000	\	\
	DpolyH3c05G002097	Single Copy	0.0000	\	\
	DpolyH4c05G002102	Single Copy	8.4899	\	\
ACAT	DpolyH1c16G000809	Single Copy	8.3981	\	\
	DpolyH2c16G000824	Single Copy	0.4575	\	\
	DpolyH3c16G000805	Single Copy	12.1809	\	\
	DpolyH4c16G000717	Single Copy	12.7826	\	\
HMGS	DpolyH1c01G000747	Single Copy	1.2229	\	\
	DpolyH2c01G000606	Single Copy	1.6925	\	\
	DpolyH3c01G000587	Single Copy	0.0000	\	\
	DpolyH4c01G000502	Single Copy	1.1761	\	\
HMGR	DpolyH1c05G002533	Single Copy	38.6953	\	\
	DpolyH2c05G002290	Single Copy	38.7128	\	\
	DpolyH3c16G000002	Single Copy	2.9164	\	\
	DpolyH4c16G000808	Single Copy	0.6270	\	\
HMGR	DpolyH1c03G000920	Single Copy	2.0853	\	\
	DpolyH2c03G001012	Single Copy	1.1402	\	\
	DpolyH3c03G000875	Single Copy	4.8168	\	\
	DpolyH4c03G000860	Single Copy	0.0000	\	\
HMGR	DpolyH1c16G000897	Single Copy	2.8593	\	\
	DpolyH2c16G000912	Single Copy	0.0000	\	\
	DpolyH3c16G000002	Single Copy	2.9164	\	\
	DpolyH4c16G000808	Single Copy	0.6270	\	\
PMK	DpolyH1c04G001224	Single Copy	4.0561	\	\
	DpolyH2c04G001204	Single Copy	0.0000	\	\
	DpolyH3c04G001323	Single Copy	10.2348	\	\
	DpolyH4c04G001222	Single Copy	1.8834	\	\
MVD	DpolyH1c10G000058	Single Copy	2.1849	\	\
	DpolyH2c10G000056	Single Copy	0.0000	\	\
	DpolyH3c10G000053	Single Copy	4.0794	\	\
	DpolyH4c10G000054	Single Copy	0.0000	\	\
DXS	DpolyH1c07G000442	Single Copy	4.6834	\	\
	DpolyH2c07G000445	Single Copy	4.5431	\	\
	DpolyH3c07G000423	Single Copy	15.5617	\	\
	DpolyH4c07G000419	Single Copy	15.5545	\	\
DXS	DpolyH2c15G000313	Single Copy	0.4499	\	\
	DpolyH3c15G000215	Single Copy	0.3607	\	\
	DpolyH4c15G000330	Single Copy	0.0179	\	\

DXS	DpolyH1c08G001381	Single Copy	0.2533	\	\
	DpolyH2c08G001404	Single Copy	0.0621	\	\
	DpolyH3c08G001413	Single Copy	0.4764	\	\
	DpolyH4c08G001251	Single Copy	0.1208	\	\
DXS	DpolyH1c05G000916	Single Copy	2.7731	\	\
	DpolyH2c05G000847	Single Copy	4.9787	\	\
	DpolyH3c05G000625	Single Copy	0.0734	\	\
	DpolyH4c05G000650	Single Copy	3.7149	\	\
DXR	DpolyH1c17G000110	Single Copy	7.7653	\	\
	DpolyH2c17G000117	Single Copy	8.1303	\	\
	DpolyH3c17G000106	Single Copy	7.3240	\	\
	DpolyH4c17G000108	Single Copy	7.2870	\	\
MCT	DpolyH1c14G000584	Single Copy	5.5353	\	\
	DpolyH2c14G000597	Single Copy	0.0000	\	\
	DpolyH3c14G000655	Single Copy	13.4324	\	\
	DpolyH4c14G000637	Single Copy	1.0112	\	\
CMK	DpolyH1c07G000783	Single Copy	7.1193	\	\
	DpolyH2c07G000769	Single Copy	0.0000	\	\
	DpolyH3c07G000725	Single Copy	4.3012	\	\
	DpolyH4c07G000666	Single Copy	0.0000	\	\
MDS	DpolyH1c01G000707	Single Copy	8.8118	\	\
	DpolyH2c01G000564	Single Copy	11.6106	\	\
	DpolyH3c01G000546	Single Copy	16.9230	\	\
	DpolyH4c01G000460	Single Copy	3.3190	\	\
HDS	DpolyH1c01G000011	Single Copy	8.2017	\	\
	DpolyH2c01G000010	Single Copy	24.4065	\	\
	DpolyH3c01G000011	Single Copy	8.2068	\	\
	DpolyH4c01G000011	Single Copy	19.2473	\	\
HDR	DpolyH1c13G000759	Single Copy	12.9679	\	\
	DpolyH2c13G000751	Single Copy	12.9440	\	\
	DpolyH3c13G000768	Single Copy	61.0421	\	\
	DpolyH4c13G000727	Single Copy	26.9645	\	\
IDI	DpolyH1c10G000605	Single Copy	11.5107	\	\
	DpolyH2c10G000615	Single Copy	9.9180	\	\
	DpolyH3c10G000637	Single Copy	23.4815	\	\
	DpolyH4c10G000607	Single Copy	13.7581	\	\
GPPS	DpolyH1c19G000867	Single Copy	9.6409	\	\
	DpolyH2c19G000872	Single Copy	6.1246	\	\
	DpolyH3c19G000859	Single Copy	10.4846	\	\
	DpolyH4c19G000854	Single Copy	0.0000	\	\
GPPS	DpolyH1c13G000434	Single Copy	3.2417	\	\
	DpolyH2c13G000419	Single Copy	0.0000	\	\
	DpolyH3c13G000412	Single Copy	6.2132	\	\

	DpolyH4c13G000384	Single Copy	5.0743	\	\
GPPS	DpolyH1c02G000261	2	0.0468	DpolyH1c02G000260	9.3846
	DpolyH2c02G000288	2	0.0468	DpolyH2c02G000287	3.1799
	DpolyH3c02G000259	2	0.0102	DpolyH3c02G000258	7.2539
	DpolyH4c02G000279	2	0.0102	DpolyH4c02G000278	0.0000
GPPS	DpolyH1c16G000373	Single Copy	0.1971	\	\
	DpolyH2c16G000392	Single Copy	0.1971	\	\
	DpolyH3c16G000446	Single Copy	0.2930	\	\
	DpolyH4c16G000448	Single Copy	0.0499	\	\
FPPS	DpolyH1c05G001621	Single Copy	6.4111	\	\
	DpolyH2c05G001382	Single Copy	12.2850	\	\
	DpolyH3c05G001308	Single Copy	7.1150	\	\
	DpolyH4c05G001323	Single Copy	14.2996	\	\
SQS	DpolyH1c08G001354	Single Copy	5.3041	\	\
	DpolyH2c08G001374	Single Copy	4.5370	\	\
	DpolyH3c08G001384	Single Copy	5.1343	\	\
	DpolyH4c08G001224	Single Copy	0.0000	\	\
SQE	DpolyH1c15G001096	Single Copy	6.3923	\	\
	DpolyH2c15G000968	Single Copy	7.3223	\	\
	DpolyH3c15G000833	Single Copy	6.6719	\	\
	DpolyH4c15G000963	Single Copy	26.1828	\	\
OSC	DpolyH1c06G000362	Single Copy	6.0742	\	\
	DpolyH2c06G000362	Single Copy	7.0954	\	\
	DpolyH3c06G000366	Single Copy	1.8898	\	\
	DpolyH4c06G000359	Single Copy	6.9683	\	\
OSC	DpolyH1c20G000187	Single Copy	8.1566	\	\
	DpolyH2c20G000226	Single Copy	6.8630	\	\
	DpolyH3c20G000209	Single Copy	0.2596	\	\
	DpolyH4c20G000068	Single Copy	14.6733	\	\
OSC	DpolyH1c12G000940	Single Copy	11.0661	\	\
	DpolyH2c12G000894	Single Copy	4.4672	\	\
	DpolyH3c12G000852	Single Copy	0.0000	\	\
	DpolyH4c12G000963	Single Copy	3.5008	\	\
OSC	DpolyH1c06G000804	Single Copy	12.5801	\	\
	DpolyH2c06G000826	Single Copy	7.9191	\	\
	DpolyH3c06G000723	Single Copy	12.5913	\	\
	DpolyH4c06G000788	Single Copy	12.7712	\	\
SMT1	DpolyH1c19G000918	Single Copy	2.9245	\	\
	DpolyH2c19G000924	Single Copy	4.4630	\	\
	DpolyH3c19G000906	Single Copy	6.1193	\	\
	DpolyH4c19G000901	Single Copy	0.0000	\	\
SMT1	DpolyH1c13G000351	Single Copy	0.0000	\	\
	DpolyH2c13G000334	Single Copy	0.0000	\	\

	DpolyH3c13G000304	3	0.0000	DpolyH3c13G000302	0.0000
				DpolyH3c13G000305	1.4030
	DpolyH4c13G000270	3	5.3598	DpolyH4c13G000264	0.0000
				DpolyH4c13G000268	0.0000
SMT2	DpolyH1c14G001040	Single Copy	34.9532	\	\
	DpolyH2c14G001023	Single Copy	30.6926	\	\
	DpolyH3c14G001071	Single Copy	30.6473	\	\
	DpolyH4c14G000970	Single Copy	30.6159	\	\
SMO1	DpolyH1c01G000803	Single Copy	0.0044	\	\
	DpolyH2c01G000659	Single Copy	0.0000	\	\
	DpolyH3c01G000641	Single Copy	0.0044	\	\
	DpolyH4c01G000553	Single Copy	0.0000	\	\
SMO1	DpolyH1c14G001311	2	0.0000	DpolyH1c14G001310	5.8277
	DpolyH2c14G001307	2	13.3962	DpolyH2c14G001306	0.0000
	DpolyH3c14G001367	2	12.0899	DpolyH3c14G001366	4.1719
	DpolyH4c14G001302	3	0.0000	DpolyH4c14G001300	15.1252
				DpolyH4c14G001301	4.3121
SMO2	DpolyH1c07G001419	Single Copy	0.4947	\	\
	DpolyH2c07G001407	Single Copy	0.4947	\	\
	DpolyH3c07G001402	Single Copy	0.1611	\	\
	DpolyH4c07G001317	Single Copy	0.1611	\	\
SMO2	DpolyH1c09G000091	Single Copy	14.2238	\	\
	DpolyH2c09G000100	Single Copy	2.8127	\	\
	DpolyH3c09G000092	Single Copy	2.7443	\	\
	DpolyH4c09G000090	Single Copy	2.1561	\	\
CPI1	DpolyH1c10G000330	Single Copy	10.1135	\	\
	DpolyH2c10G000326	Single Copy	0.0000	\	\
	DpolyH3c10G000324	Single Copy	5.0917	\	\
	DpolyH4c10G000338	Single Copy	0.0000	\	\
CYP51A	DpolyH1c10G001000	Single Copy	8.9712	\	\
	DpolyH2c10G000939	Single Copy	9.9623	\	\
	DpolyH3c10G000868	Single Copy	8.6304	\	\
	DpolyH4c10G000890	Single Copy	8.6906	\	\
8,7-SI	DpolyH1c05G001939	Single Copy	6.7680	\	\
	DpolyH2c05G001704	Single Copy	0.0000	\	\
	DpolyH3c05G001634	Single Copy	8.3478	\	\
	DpolyH4c05G001637	Single Copy	0.0000	\	\
8,7-SI	DpolyH1c10G001031	Single Copy	13.5793	\	\
	DpolyH2c10G000971	Single Copy	0.0000	\	\
	DpolyH3c10G000899	Single Copy	1.8968	\	\
	DpolyH4c10G000921	Single Copy	0.0000	\	\
C5-SD	DpolyH1c05G001490	Single Copy	18.6645	\	\
	DpolyH2c05G001246	Single Copy	7.9872	\	\

	DpolyH3c05G001164	Single Copy	9.6117	\	\
	DpolyH4c05G001186	Single Copy	6.1470	\	\
C5-SD	DpolyH1c17G000292	Single Copy	4.8341	\	\
	DpolyH2c17G000301	Single Copy	3.2002	\	\
	DpolyH3c17G000291	Single Copy	7.4975	\	\
7-DR	DpolyH1c03G000046	Single Copy	7.7287	\	\
	DpolyH2c03G000045	Single Copy	5.7707	\	\
	DpolyH3c03G000049	Single Copy	5.7568	\	\
	DpolyH4c03G000003	Single Copy	4.7931	\	\
DWF1	DpolyH1c01G000548	Single Copy	14.5256	\	\
	DpolyH2c01G000428	Single Copy	26.5251	\	\
	DpolyH3c01G000467	Single Copy	1.3866	\	\
	DpolyH4c01G000370	Single Copy	15.5318	\	\
DWF1	DpolyH1c14G000255	Single Copy	0.0000	\	\
	DpolyH2c14G000257	Single Copy	0.0000	\	\
	DpolyH3c14G000277	Single Copy	0.0241	\	\
	DpolyH4c14G000277	Single Copy	0.0241	\	\
DWF1	DpolyH1c11G000730	Single Copy	0.0035	\	\
	DpolyH2c11G000733	Single Copy	0.0035	\	\
	DpolyH3c11G000558	Single Copy	0.0142	\	\
	DpolyH4c11G000720	Single Copy	0.0887	\	\
CYP90B	DpolyH1c13G000658	2	3.6401	DpolyH1c13G000663	29.0853
	DpolyH2c13G000652	3	0.0000	DpolyH2c13G000652	0.0000
				DpolyH2c13G000661	2.9296
	DpolyH3c13G000669	2	6.7018	DpolyH3c13G000673	0.0075
	DpolyH4c13G000633	2	4.7554	DpolyH4c13G000636	5.2048
CYP90B	DpolyH1c02G000111	2	0.0000	DpolyH1c02G000113	0.1420
	DpolyH2c02G000118	2	0.0000	DpolyH2c02G000120	0.1420
	DpolyH3c02G000111	2	0.0000	DpolyH3c02G000113	0.3492
	DpolyH4c02G000120	2	0.0000	DpolyH4c02G000122	0.0000
CYP72A	DpolyH1c20G000762	7	0.3324	DpolyH1c20G000756	0.4698
				DpolyH1c20G000757	14.8917
				DpolyH1c20G000758	5.6252
				DpolyH1c20G000760	0.4512
				DpolyH1c20G000761	0.2002
				DpolyH1c20G000764	6.5627
	DpolyH2c20G000808	6	0.0709	DpolyH2c20G000804	3.4265
				DpolyH2c20G000805	47.2290
				DpolyH2c20G000806	0.0000
				DpolyH2c20G000807	0.0411
				DpolyH2c20G000809	5.5809
	DpolyH3c20G000769	7	0.0000	DpolyH3c20G000765	0.0000
				DpolyH3c20G000766	8.7940

				DpolyH3c20G000767	0.4084
				DpolyH3c20G000768	0.2721
				DpolyH3c20G000770	0.1431
				DpolyH3c20G000771	4.6258
	DpolyH4c20G000682	7	0.0000	DpolyH4c20G000678	3.3188
				DpolyH4c20G000679	8.7666
				DpolyH4c20G000680	0.0000
				DpolyH4c20G000681	0.2721
				DpolyH4c20G000683	0.1431
				DpolyH4c20G000684	0.0000
CYP94	DpolyH1c06G000585	3	4.3222	DpolyH1c06G000584	0.0489
				DpolyH1c06G000587	0.0492
	DpolyH2c06G000596	5	0.1237	DpolyH2c06G000594	5.4549
				DpolyH2c06G000595	0.0489
				DpolyH2c06G000598	0.0492
				DpolyH2c06G000603	0.1769
	DpolyH4c06G000648	3	4.7913	DpolyH4c06G000654	0.0396
				DpolyH4c06G000657	0.0695
CYP94	DpolyH1c13G000664	3	6.2400	DpolyH1c13G000659	5.1755
				DpolyH1c13G000661	0.0000
	DpolyH2c13G000656	2	0.0000	DpolyH2c13G000662	6.7597
	DpolyH3c13G000674	Single Copy	0.0800	\	\
	DpolyH4c13G000639	Single Copy	0.0896	\	\
CYP94	DpolyH1c12G000519	Single Copy	3.7478	\	\
	DpolyH2c12G000479	2	1.1983	DpolyH2c12G000478	1.6276
	DpolyH3c12G000453	2	0.0000	DpolyH3c12G000451	0.0000
	DpolyH4c12G000535	Single Copy	0.0000	\	\
CYP94	DpolyH1c07G000715	Single Copy	5.1712	\	\
	DpolyH2c07G000688	Single Copy	3.2979	\	\
	DpolyH3c07G000651	Single Copy	6.7684	\	\
	DpolyH4c07G000583	Single Copy	1.0725	\	\
CYP94	DpolyH1c07G000801	Single Copy	19.0640	\	\
	DpolyH2c07G000793	Single Copy	19.0361	\	\
	DpolyH3c07G000767	Single Copy	12.7105	\	\
	DpolyH4c07G000704	Single Copy	12.7021	\	\
CYP94	DpolyH1c10G000047	Single Copy	14.3327	\	\
	DpolyH2c10G000045	Single Copy	14.3101	\	\
	DpolyH3c10G000043	Single Copy	16.7669	\	\
	DpolyH4c10G000045	Single Copy	16.7348	\	\
CYP94	DpolyH1c02G000112	Single Copy	0.0112	\	\
	DpolyH2c02G000119	Single Copy	0.0000	\	\
	DpolyH3c02G000112	Single Copy	0.0023	\	\
	DpolyH4c02G000121	Single Copy	0.0023	\	\

CYP94	DpolyH1c08G000725	Single Copy	6.3711	\	\
	DpolyH2c08G000761	Single Copy	1.9926	\	\
	DpolyH3c08G000780	Single Copy	4.5969	\	\
	DpolyH4c08G000606	Single Copy	0.0000	\	\
S3GT	DpolyH1c01G000154	Single Copy	0.7607	\	\
	DpolyH2c01G000152	Single Copy	1.2216	\	\
	DpolyH3c01G000155	Single Copy	0.5814	\	\
	DpolyH4c01G000145	Single Copy	2.2876	\	\
S3GT	DpolyH3c02G000607	Single Copy	13.2215	\	\
	DpolyH4c02G000636	Single Copy	13.2041	\	\
S3GT	DpolyH1c17G001003	Single Copy	1.3060	\	\
	DpolyH2c17G001037	Single Copy	0.3495	\	\
	DpolyH3c17G000912	Single Copy	3.4569	\	\
	DpolyH4c17G000831	Single Copy	0.0000	\	\
S3GT	DpolyH1c14G000769	Single Copy	3.9077	\	\
	DpolyH2c14G000797	Single Copy	2.2187	\	\
	DpolyH3c14G000842	Single Copy	2.8442	\	\
	DpolyH4c14G000811	Single Copy	0.0000	\	\

Note: Using synteny genes between *D. alata* and *D. polystachya* genomes, we estimated a background gene retention rate of 91.31%, calculated as the ratio of *D. polystachya* syntenic genes to (D. alata syntenic genes × 4). The dioscin biosynthesis pathway were generally over-retained after *D. polystachya* genome tetraploidization compared with *D. alata* (98.43% vs. 91.31%). Nearly all genes in dioscin biosynthesis pathway have four alleles with limited gene fractionation. Furthermore, many of them exhibited allelic expression imbalance in tuber.

Supplemental Table 12. **Summary statistics of filtered reads and comparison to the reference genome.**

Sample	Total raw reads (M)	Total clean reads (M)	Total clean bases (Gb)	Clean reads Q20 (%)	Clean reads Q30 (%)	Clean reads ratio (%)	Total mapping (%)	Uniquely mapping (%)
T1_1	56.91	56.54	8.48	98.34	94.83	99.35	95.83	89.02
T1_2	55.61	55.27	8.29	98.43	95.05	99.39	96.05	89.83
T1_3	54.69	54.31	8.15	98.28	94.68	99.31	95.48	92.59
T2_1	63.71	63.31	9.50	98.41	95.00	99.37	96.45	88.23
T2_2	61.25	60.78	9.12	98.13	94.31	99.23	96.03	88.35
T2_3	57.42	57.04	8.56	98.30	94.73	99.34	96.12	89.88
T3_1	55.44	55.07	8.26	98.42	95.01	99.33	96.24	89.06
T3_2	60.76	60.36	9.05	98.32	94.77	99.34	96.34	87.47
T3_3	60.26	59.87	8.98	98.34	94.82	99.35	94.48	88.98
T4_1	53.39	52.93	7.94	98.38	94.90	99.14	96.70	80.27
T4_2	61.32	60.91	9.14	98.39	94.94	99.33	96.30	84.34
T4_3	55.96	55.49	8.32	98.38	94.90	99.16	96.59	89.49
Peel_1	42.21	41.33	6.20	97.80	93.82	97.92	95.47	93.23
Peel_2	43.31	42.48	6.37	97.61	92.46	98.08	91.23	89.02
Peel_3	42.53	41.87	6.28	97.40	93.02	98.45	77.25	75.07
Flesh_1	43.35	42.48	6.37	97.49	92.09	97.99	87.05	84.56
Flesh_2	43.24	42.35	6.35	97.50	92.05	97.94	96.12	93.58
Flesh_3	43.36	42.49	6.37	97.52	92.22	97.99	87.99	85.06

Supplemental Table 13. **RNA-seq analysis of genes involved in dioscin biosynthesis in yam tubers.**

Gene name	Gene ID	T1	T2	T3	T4	Peel	Flesh
*DpGPPS1*_Hap1	DpolyH1c02G000261	1.10	0.07	0.11	0.10	4.06	5.74
*DpGPPS1*_Hap2	DpolyH2c02G000288	1.10	0.07	0.11	0.10	4.06	5.74
*DpGPPS1*_Hap3	DpolyH3c02G000259	0.13	0.02	0.00	0.00	1.96	12.21
*DpGPPS1*_Hap4	DpolyH4c02G000279	0.13	0.02	0.00	0.00	1.96	12.21
*DpGPPS2*_Hap1	DpolyH1c13G000434	5.12	2.78	3.29	1.18	14.07	4.80
*DpGPPS2*_Hap2	DpolyH2c13G000419	5.12	2.78	3.29	1.18	14.07	4.80
*DpGPPS2*_Hap3	DpolyH3c13G000412	3.47	5.82	4.88	3.14	16.07	12.50
*DpGPPS2*_Hap4	DpolyH4c13G000384	8.77	5.05	6.59	0.81	47.79	27.17
*DpGPPS3*_Hap1	DpolyH1c16G000373	12.19	19.93	8.98	2.56	1.61	8.78
*DpGPPS3*_Hap2	DpolyH2c16G000392	12.19	19.93	8.98	2.56	1.61	8.78
*DpGPPS3*_Hap3	DpolyH3c16G000446	25.36	37.57	11.88	5.26	0.75	6.52
*DpGPPS3*_Hap4	DpolyH4c16G000448	25.36	37.57	11.88	5.26	0.75	6.52
*DpGPPS4*_Hap1	DpolyH1c19G000867	26.04	15.26	15.67	11.33	9.39	5.14
*DpGPPS4*_Hap2	DpolyH2c19G000872	14.22	9.93	10.36	7.47	5.92	2.27
*DpGPPS4*_Hap3	DpolyH3c19G000859	4.25	3.26	2.77	3.24	3.02	9.98
*DpGPPS4*_Hap4	DpolyH4c19G000854	4.25	3.26	2.77	3.24	3.02	9.98
*DpFPPS*_Hap1	DpolyH1c05G001621	21.80	20.10	21.13	12.74	5.03	8.61
*DpFPPS*_Hap2	DpolyH2c05G001382	21.80	20.10	21.13	12.74	5.03	8.61
*DpFPPS*_Hap3	DpolyH3c05G001308	65.40	45.33	57.04	32.14	35.24	40.12
*DpFPPS*_Hap4	DpolyH4c05G001323	65.40	45.33	57.04	32.14	35.24	40.12
*DpSQS*_Hap1	DpolyH1c08G001354	1.72	1.17	1.60	1.20	2.94	0.99
*DpSQS*_Hap2	DpolyH2c08G001374	20.12	18.08	16.72	10.97	15.05	4.35
*DpSQS*_Hap3	DpolyH3c08G001384	10.84	7.49	10.24	4.76	12.37	11.50
*DpSQS*_Hap4	DpolyH4c08G001224	1.72	1.17	1.60	1.20	2.94	0.99
*DpSQE*_Hap1	DpolyH1c15G001096	4.27	5.04	6.06	5.73	7.69	15.81
*DpSQE*_Hap2	DpolyH2c15G000968	52.98	39.97	42.38	52.81	21.13	11.74
*DpSQE*_Hap3	DpolyH3c15G000833	4.27	5.04	6.06	5.73	7.69	15.81
*DpSQE*_Hap4	DpolyH4c15G000963	77.94	76.43	62.91	69.44	33.28	29.95
*DpSMO3-1*_Hap1	DpolyH1c07G001419	2.60	0.09	0.14	0.10	0.31	0.08
*DpSMO3-1*_Hap2	DpolyH2c07G001407	2.60	0.09	0.14	0.10	0.31	0.08
*DpSMO3-1*_Hap3	DpolyH3c07G001402	1.80	0.32	0.75	0.13	0.47	0.00
*DpSMO3-1*_Hap4	DpolyH4c07G001317	1.80	0.32	0.75	0.13	0.47	0.00
*DpSMO3-2*_Hap1	DpolyH1c09G000091	2.84	2.04	2.89	1.52	9.96	12.37
*DpSMO3-2*_Hap2	DpolyH2c09G000100	2.40	4.04	1.89	1.69	0.00	0.00
*DpSMO3-2*_Hap3	DpolyH3c09G000092	2.84	2.04	2.89	1.52	9.96	12.37
*DpSMO3-2*_Hap4	DpolyH4c09G000090	2.84	2.04	2.89	1.52	9.96	12.37
*DpCPI*_Hap1	DpolyH1c10G000330	7.10	4.78	5.75	3.14	9.02	8.21
*DpCPI*_Hap2	DpolyH2c10G000326	7.10	4.78	5.75	3.14	9.02	8.21
*DpCPI*_Hap3	DpolyH3c10G000324	4.67	3.74	4.27	2.69	6.44	4.75
*DpCPI*_Hap4	DpolyH4c10G000338	4.67	3.74	4.27	2.69	6.44	4.75
*Dp8,7 SI1*_Hap1	DpolyH1c05G001939	3.35	1.91	3.02	1.36	5.49	2.80

*Dp8,7 SI1*_Hap2	DpolyH2c05G001704	3.35	1.91	3.02	1.36	5.49	2.80
*Dp8,7 SI1*_Hap3	DpolyH3c05G001634	6.15	3.56	4.44	2.57	11.74	4.73
*Dp8,7 SI1*_Hap4	DpolyH4c05G001637	6.15	3.56	4.44	2.57	11.74	4.73
*Dp8,7 SI2*_Hap1	DpolyH1c10G001031	0.00	0.41	0.32	1.16	0.92	0.59
*Dp8,7 SI2*_Hap2	DpolyH2c10G000971	0.00	0.41	0.32	1.16	0.92	0.59
*Dp8,7 SI2*_Hap3	DpolyH3c10G000899	3.97	0.16	0.15	0.20	0.42	0.26
*Dp8,7 SI2*_Hap4	DpolyH4c10G000921	3.97	0.16	0.15	0.20	0.42	0.26
*DpC5-SD1*_Hap1	DpolyH1c05G001490	7.21	3.86	5.03	4.82	9.17	7.33
*DpC5-SD1*_Hap2	DpolyH2c05G001246	7.21	3.86	5.03	4.82	9.17	7.33
*DpC5-SD1*_Hap3	DpolyH3c05G001164	7.21	3.86	5.03	4.82	9.17	7.33
*DpC5-SD1*_Hap4	DpolyH4c05G001186	7.21	3.86	5.03	4.82	9.17	7.33
*DpC5-SD2*_Hap1	DpolyH1c17G000292	38.16	33.40	50.67	24.65	66.22	44.49
*DpC5-SD2*_Hap2	DpolyH2c17G000301	32.61	28.16	45.45	20.91	48.66	63.35
*DpC5-SD2*_Hap3	DpolyH3c17G000291	61.54	53.14	85.52	39.03	46.54	13.58
*Dp7-DR*_Hap1	DpolyH1c03G000046	22.77	29.92	28.46	40.18	38.44	23.80
*Dp7-DR*_Hap2	DpolyH2c03G000045	22.77	29.92	28.46	40.18	38.44	23.80
*Dp7-DR*_Hap3	DpolyH3c03G000049	22.77	29.92	28.46	40.18	38.44	23.80
*Dp7-DR*_Hap4	DpolyH4c03G000003	22.77	29.92	28.46	40.18	38.44	23.80
*DpDWF1-1*_Hap1	DpolyH1c01G000548	0.00	0.00	0.28	0.00	0.00	0.00
*DpDWF1-1*_Hap2	DpolyH2c01G000428	2.62	0.46	3.31	0.00	8.09	13.97
*DpDWF1-1*_Hap3	DpolyH3c01G000467	3.61	3.07	5.58	1.85	28.99	41.34
*DpDWF1-1*_Hap4	DpolyH4c01G000370	14.99	10.15	15.45	9.87	11.04	5.34
*DpDWF1-2*_Hap1	DpolyH1c11G000730	0.52	1.02	0.45	0.89	0.13	0.67
*DpDWF1-2*_Hap2	DpolyH2c11G000733	0.52	1.02	0.45	0.89	0.13	0.67
*DpDWF1-2*_Hap3	DpolyH3c11G000558	0.00	0.00	0.00	0.00	0.00	0.00
*DpDWF1-2*_Hap4	DpolyH4c11G000720	0.00	0.00	0.00	0.00	0.00	0.00
*DpCYP51A*_Hap1	DpolyH1c10G001000	11.73	12.82	9.84	5.89	17.39	30.41
*DpCYP51A*_Hap2	DpolyH2c10G000939	11.73	12.82	9.84	5.89	17.39	30.41
*DpCYP51A*_Hap3	DpolyH3c10G000868	21.81	19.97	15.87	12.62	14.59	16.26
*DpCYP51A*_Hap4	DpolyH4c10G000890	21.81	19.97	15.87	12.62	14.59	16.26
*DpCYP94A1*_Hap1	DpolyH1c06G000585	1.07	0.94	0.81	0.33	1.51	2.00
*DpCYP94A1*_Hap2	DpolyH2c06G000596	1.95	0.90	1.26	0.73	9.23	1.45
*DpCYP94A1*_Hap4	DpolyH4c06G000648	0.41	0.11	0.05	0.00	12.91	9.06
*DpCYP94A1*_Hap4	DpolyH4c06G000654	0.09	0.00	0.00	0.02	2.52	0.00
*DpCYP94A2*_Hap1	DpolyH1c07G000715	1.68	1.85	1.00	1.38	0.79	0.40
*DpCYP94A2*_Hap2	DpolyH2c07G000688	1.68	1.85	1.00	1.38	0.79	0.40
*DpCYP94A2*_Hap3	DpolyH3c07G000651	1.85	2.17	0.98	1.22	1.75	0.97
*DpCYP94A2*_Hap4	DpolyH4c07G000583	1.85	2.17	0.98	1.22	1.75	0.97
*DpCYP94A3*_Hap1	DpolyH1c07G000801	1.71	1.87	1.60	1.47	2.78	2.88
*DpCYP94A3*_Hap2	DpolyH2c07G000793	1.71	1.87	1.60	1.47	2.78	2.88
*DpCYP94A3*_Hap3	DpolyH3c07G000767	0.78	1.32	1.36	1.00	1.16	1.87
*DpCYP94A3*_Hap4	DpolyH4c07G000704	0.78	1.32	1.36	1.00	1.16	1.87
*DpCYP94A4*_Hap1	DpolyH1c08G000725	9.72	7.13	5.79	3.50	4.57	1.55
*DpCYP94A4*_Hap2	DpolyH2c08G000761	9.72	7.13	5.79	3.50	4.57	1.55

*DpCYP94A4*_Hap3	DpolyH3c08G000780	14.53	13.62	9.42	4.02	30.93	12.37
*DpCYP94A4*_Hap4	DpolyH4c08G000606	14.53	13.62	9.42	4.02	30.93	12.37
*DpCYP94A5*_Hap1	DpolyH1c12G000519	0.54	0.40	0.34	0.50	4.73	0.66
*DpCYP94A5*_Hap2	DpolyH2c12G000479	0.09	0.07	0.06	0.07	0.13	0.17
*DpCYP94A5*_Hap3	DpolyH3c12G000453	0.09	0.07	0.06	0.07	0.13	0.17
*DpCYP94A5*_Hap4	DpolyH4c12G000535	0.54	0.40	0.34	0.50	4.73	0.66
*DpCYP94C1*_Hap1	DpolyH1c10G000047	62.77	42.77	17.51	12.89	27.13	12.68
*DpCYP94C1*_Hap2	DpolyH2c10G000045	62.77	42.77	17.51	12.89	27.13	12.68
*DpCYP94C1*_Hap3	DpolyH3c10G000043	59.22	46.40	15.99	13.97	39.95	18.58
*DpCYP94C1*_Hap4	DpolyH4c10G000045	59.22	46.40	15.99	13.97	39.95	18.58
*DpS3GT1*_Hap1	DpolyH1c01G000154	0.37	0.42	0.86	0.57	0.24	0.16
*DpS3GT1*_Hap2	DpolyH2c01G000152	0.81	0.57	0.52	0.60	1.10	1.21
*DpS3GT1*_Hap3	DpolyH3c01G000155	0.37	0.42	0.86	0.57	0.24	0.16
*DpS3GT1*_Hap4	DpolyH4c01G000145	0.51	0.37	0.32	0.57	0.26	0.66
*DpS3GT2*_Hap3	DpolyH3c02G000607	15.42	17.03	16.18	17.97	26.19	23.03
*DpS3GT2*_Hap4	DpolyH4c02G000636	15.42	17.03	16.18	17.97	26.19	23.03
*DpS3GT3*_Hap1	DpolyH1c14G000769	1.07	1.08	0.84	0.51	2.51	1.72
*DpS3GT3*_Hap2	DpolyH2c14G000797	1.07	1.08	0.84	0.51	2.51	1.72
*DpS3GT3*_Hap3	DpolyH3c14G000842	0.49	0.50	0.35	0.12	0.67	0.13
*DpS3GT3*_Hap4	DpolyH4c14G000811	0.49	0.50	0.35	0.12	0.67	0.13
*DpS3GT4*_Hap1	DpolyH1c17G001003	0.34	0.53	0.51	0.36	0.23	0.39
*DpS3GT4*_Hap2	DpolyH2c17G001037	0.34	0.53	0.51	0.36	0.23	0.39
*DpS3GT4*_Hap3	DpolyH3c17G000912	1.99	2.14	3.10	3.10	1.41	0.98
*DpS3GT4*_Hap4	DpolyH4c17G000831	1.99	2.14	3.10	3.10	1.41	0.98

Supplemental Table 14. Protein sequences used for reconstructing the 7-DR phylogenetic tree.

>*Sorghum bicolor* 7-DR1

MAKPKPSSAGAKSAAAAAAPAPPATVHSALVTYTSMLALLSLCPPFVILLWYTMVHADGSVVRTYEHLREHGVLEGLKAIWPMPTLVAWKIIFGFGLFEAVLQLLLPGKRFEGPISPAGNVPVYKANGLQA
YAVTLITYLGLWWFGIFNPAIVYDHLGEIYSALVFGSFVFCILLYIKGHVAPSSSDSGSSGNVIIDFYWGMELYPRIGKYFDIKVFTNCRFGMMSWAVLAVTYCIKQYEMNGRVADSMLVNTALMLIYITKFF
WWESGYWCTMDIAHDRAGFYICWGCLVWVPSIYTSPGMYLVNHPVNLGPQLALSILLAGMLCIYINYDCDRQRQEFRRTNGKCSVWGKAPSKIVASYQTTKGETKTSLLLTSGWWGLSRHFHYVPEILSA
FFWTVPALFNHFLPYFYVIFLTILLFDRAKRDDDRCSSKYGKYWKTYCNKVPYRVIPGIY

>*Zea mays* 7-DR1

MAKPKPSSAGAKPTAAAPPVTVHSALVTYTSMLALLSLCPPFVILLWYTMVHADGSVVRTYEHLRDHGVLEGLKAIWPMPTLVAWKIIFGFGLFEAVLQLLLPGKRFEGPISPAGNVPVYKANGLQAYAVT
LITYLGLWWFGIFNPAIVYDHLGEIYSALVFGSFVFCIFLYIKGHVFPSSSDSGSSGNVIIDFYWGMELYPRIGKYFDIKVFTNCRFGMMSWAVLAVTYCIKQYEMNGRVADSMLVNTALMLIYITKFFWWES
GYWCTMDIAHDRAGFYICWGCLVWVPSIYTSPGMYLVNHPLNLGPQLALSILLAGMLCIYINYDCDRQRQEFRRTNGKCSVWGKAPSKIVASYQTTKGETKTSLLLTSGWWGLSRHFHYVPEILSAFFWTV
PALFNHFLPYFYVIFLTILLFDRAKRDDDRCSSKYGKYWKIYCNKVPYRVIPGIY

>*Vitis vinifera* 7-DR1

MISLLTLCPPFVILLWYTMVHADGSVLQTWDYLKQHGVQGFIDIWPRPTAIAWKLIACYAAFEAALQLFLPGKTVEGPISPCGNRPVYKANGMQAYAVTLITYLSLWWFGIFNPAIVYDHLGEIYSALIFGSL
IFCIFLYIKGHVAPSSTDSGSSGNIIIDFYWGMELYPRIGKNFDIKVFTNCRFGMMSWAVLAVTYCIKQYEENGKVADSMLVNTILMLVYVTKFFWWEAGYWNTMDIAHDRAGFYICWGCLVWVPSIYTSP
GMYLVNHPVNLGTQLAIYILAAGITCIYINYDCDRQRQEFRRTNGKCLVWGKAPSKIVASYTTTSGETKSSLLLTSGWWGLSRHFHYVPEILAAFFWTVPALFGHFLPYFYVLFLTILLFDRAKRDDDRCRSK
YGKYWKKYCEKVRYRIIPGIY

>*Nicotiana tabacum* 7-DR2-2

MAESQLVHPPLVTYLSMIALLTLAPPFVILMWYTNVHADGSVLQTFNYLRENGLQGLIDIWPRPSAVAGKIIICYALFEAALQLLLPGKMVQGPISPTGHRPVYKANGMAAYVVTLITFGIFNPTLVYDHLGE
ILSALNFGSLIFCLFLYIKGHVAPSSTDHGSSGNIIIDFYWGMELYPRIGKHFDIKVFTNCRFGMISWGVLPITYCIKQYEEYGSLSDSMLVNTILTLVWLLHMLGMLSMASMYIYFSWNLAIYILVAGVLCVYI
NYDCDRQRQEWGLARHFHYVPEILASFFWSVPALFNHFIPYFYVIYLTILLFDRAKRDDDRCKSKYGKYWKLYCEKVPYRVIPGIY

>*Nicotiana tabacum* 7-DR2

MAESQLVHPPLVTYLSMMALLTLAPPFVILMWYTNVLADGSVLQTFNYLRENGLQGLVDIWPRPSAVAGKIIICYALFEAALQLLLPGKTVQGPISPTGHRPVYKGHVAPSSTDHGSSGNIIIDFYWGMELYP
RVGKHFDIKVFTNCRFGMISWGVLPITYCIKQYEEYGSLSDSMLVNTILTLVYVTKFFWWEAGYWNTMDIAHDRAGFYICWGCLVWLPCIYTSPGMYLVKQPVNLGLQLAIYILVAGVLCVYINYDCDRQ
REEWGLARHFHYVPEILASFFWSVPALFNHFIPYFYVIYLTILLFDRAKRDDDRCKSKYGKYWKLYCEKVPYRVIPGIY

>*Capsicum annuum* 7-DR2

MGDSQLVHPPLFTYISMLTLLTIVPPFVILMWYTNVHADGSVLQTFNFLKENGLQGLIDIWPRPTAVAGKIIICYALFEAILQLLLPGKRVEGPISPTGHRPVYKANGVAAYIVTLITYLSLWWFGIFNPTIVYD
HLGEILSTLNFGSLIFCLFLYIKGHVAPSSTDHGSSGNIIIDYYWGMELYPRIGKHFDIKVFTNCRFGMISWAVLPITYCIKQYEEYGNLSDSMLVHTIITLVYVTKFFWWEAGYWNTMDIAHDRAGFYICWGC
LVFLPCIYTSPGMYLVKHPVNLGPQLAISILVAGILCVYINYDCDRQRQEFRKTNGKALVWGRPPSKIVASYTTTTGETKTSLLLTSGWWGLSRHFHYVPEILASFFWCVPALFNHFIPYFYVIYLIVLLLDRA
KRDDERCKSKYGKYWKKYCDKVPYRVIPGIY

>*Capsicum annuum* 7-DR1

MAETKLVHSPLLTYASMLTLLSFTPPFVILMWYTNVHADGSILKTFNYLRENGLQGLINIWPKPTAIAGKLIICYALFEAALQLLLPGKRVEGPISPTGHRPVYKANGMQAYAVTLITYISLWWFGIFNPAIVY
DHLGEIFSTLIFGSLIFCFFLYIKGHVAPSSTDSGSSGNIIVDFYWGMELYPRIGKHFDIKVFTNCRFGMMSWAVLAVTYCIKQHEEYGRVSDSMLVNTILMLVYVTKFFWWEAGYWNTMDIAHDRAGFYIC
WGCLVWVPSIYTSPGMYLVKQPVHLGLQLALYILVAGLLCIYINYDCDRQRQEFRRTNGKCTVWGKTPSKIVATYTTTSGEKKTSLLLTSGWWGLARHFHYVPEILAAFFWSVPALFNHFIPYFYVVFLMIL
LFDRAKRDDDRCKAKYGKYWKLYCEKVPYRIIPGIY

>*Solanum tuberosum* 7-DR2

MAESQLVHPPLFTYISMLALLTLVPPFVILMWYTNVHADGSVLQTFNYLKENGLQGLIDIWPRPTAIAGKIIICYALFEATLQLLLPGKRVQGPISPTGHRPVYKANGMAAYTVTLITYLSLWWFGIFNPTVVY
DHLGEILSTLNFGSLIFCLFLYIKGHVAPSSTDHGSSGNIIVDYYWGMELYPRIGKHFDIKVFTNCRFGMISWGLLPITYCIKQYEEYGSLSDSMLIHTIITLVYVTKFFWWEAGYWNTMDIAHDRAGFYICWG
CLVFLPCMYTSPGMYLVKHPVNLGPQLAISILVAGILCVYINYDCDRQRQEFRRTNGKALVWGKAPSKIVASYTTTTGETKSSLLLTSGWWGLSRHFHYVPEILASFFWSVPALFNHIMPYFYVIYLTGLLLD
RAKRDDERCKSKYGKYWKKYCEKVPYRVVPGIY

>*Solanum tuberosum* 7-DR1

MVENKLVHSPLITYGSMLSLLSFTPPFVILMWYTNEHADGSILKTFNHLRENGLQGLIDIWPKPTAIAGKLIICYALFEAALQLLLPGKTVEGPISPTGHRPVYKANGMAAYAVTLITYISLWWFGIFNPAIVYD
HLGEILSTLIFGSLVFCVLLYIKGHVAPSSTDSGSSGNIIVDFYWGMELYPRIGKHFDIKVFTNCRFGMMSWAVLAVTYCIKQHEEYGRVSDSMLVNTILMLVYVTKFFWWEAGYWNTMDIAHDRAGFYIC
WGCLVWVPSIYTSPGMYLVKQPVNLGLQLALYILVAGLLCIYINYDCDRQRQEFRRTNGKCTVWGKTPSKIVAAYTTTSGEKKTSLLLTSGWWGLARHFHYVPEILAAFFWSVPALFNHFIPYFYVIFLIILL
LDRAKRDDDRCKAKYGKYWKLYCEKVPYRVIPGIY

>*Arabidopsis thaliana* DWF5

MAETVHSPIVTYASMLSLLAFCPPFVILLWYTMVHQDGSVTQTFGFFWENGVQGLINIWPRPTLIAWKIIFCYGAFEAILQLLLPGKRVEGPISPAGNRPVYKANGLAAYFVTLATYLGLWWFGIFNPAIVYD
HLGEIFSALIFGSFIFCVLLYIKGHVAPSSSDSGSCGNLIIDFYWGMELYPRIGKSFDIKVFTNCRFGMMSWAVLAVTYCIKQYEINGKVSDSMLVNTILMLVYVTKFFWWEAGYWNTMDIAHDRAGFYICW
GCLVWVPSVYTSPGMYLVNHPVELGTQLAIYILVAGILCIYINYDCDRQRQEFRRTNGKCLVWGRAPSKIVASYTTTSGETKTSLLLTSGWWGLARHFHYVPEILSAFFWTVPALFDNFLAYFYVIFLTLLLF
DRAKRDDDRCRSKYGKYWKLYCEKVKYRIIPGIY

>*Anemarrhena asphodeloides* 7DR1

SKTVHSAIVTYTSMISLLTLCPPFVILLWYSMVHADGSILQTFEYLKQHGLQGLKDIWPTPSLTAWKIIAVFGAFEAFLQLALPGKRVEGPISPTGHIPVYKANGLQAYAVTLITYVGLWWFGIFDPAIVYDHL
GEIFSALVVGSLIFCVFLYLKGHIAPSSTDSGSSGNVIIDFYWGMELYPRIGKSFDIKVFTNCRFGMMSWAVLAVTYCIKQYEMNGRVADSMLVNTVLMLVYITKFFWWEAGYWCTMDIAHDRAGFYICW
GCLVWVPSVYTSPGMYLVNHPVHLGTKLAVSILVVGLLCIYINYDCDRQRQEFRRTNGKCLIWGKAPSKIVASYTTSKGETKTSLLLTSGWWGLARHFHYAPEILAAFCWSVPALFNHFLPYFYVVFLTILL
VDRAKRDDDRCSSKYKKYWKTYCDKVPYRILPGIY

>*Solanum lycopersicum* 7-DR2

MAESQLVHPPLFTYISMITLLTLVPPFVILMWYTNVHADGSVLQTYNYLKENGLQGLIDIWPRPTAIAGKIIICYALFEATLQLLLPGKRVEGPISPTGHRPVYKANGMAAYTVTLITYLSLWWFGIFNPTIVYD
HLGEILSTLNFGSLIFCLFLYIKGHVAPSSTDHGSSGNIIVDYYWGMELYPRIGKHFDIKVFTNCRFGMISWGLLPITYCIKQYEEYGSLSDSMLIHTIITLVYVTKFFWWEAGYWNTMDIAHDRAGFYICWGC
LVFLPCMYTSPGMYLVKHPVNLGPQLAISILVAGILCVYINYDCDRQRQEFRRTNGKALVWGKAPSKIVASYTTTTGETKTSILLTSGWWGLSRHFHYVPEILASFFWSVPALLNHFMPYIYVIYLTGLLLDR
AKRDDERCKSKYGKYWKKYCEKVPYRVVPGIY

>*Solanum lycopersicum* 7-DR1

MVENKLVHSPLITYGSMLSLLSFTPPFVILMWYTNVHADGSILKTFNHLRENGLQGLIDIWPKPTAIAGKLIICYALFEAALQLLLPGKTVEGPISPTGHRPVYKANGMAAYAVTLITYISLWWFGIFNPAIVY
DHLGEIFSTLIFGSLVFCVLLYIKGHVAPSSTDSGSSGNIIVDFYWGMELYPRIGKHFDIKVFTNCRFGMMSWAVLAVTYCIKQHEEYGRVSDSMLVNTILMLVYVTKFFWWEAGYWNTMDIAHDRAGFYI
CWGCLVWVPSIYTSPGMYLVKQPVNLGLQLSLYILVAGLLCIYINYDCDRQRQEFRRTNGKCTVWGKTPSKIVAAYTTTSGEKKTSLLLTSGWWGLARHFHYVPEILAAFFWSVPALFNHFIPYFYVIFLIIL
LLDRAKRDDDRCKAKYGKYWKLYCEKVPYRVIPGIY

>*Physcomitrella patens* 7-DR1-1

MGAQGDQKLTRSQYAKEPISVHSKMVTYVSMTALITLCPAFVMFLWHTMVNLDGSTTKYLELYKVEGIDGVLKTWPMPTMNALKIILSFAAFEAFLQLYIPGEVHVGPVSPAGNRPVYKNNGFSCYIITLV
SYYGLYKSGYFNPAVVYDHLGEIYFTLVVGSFFVCILLYIKGHVAPSSSDCGSSGNVLVDFYWGMELYPRIGQNFDIKVFTNCRFGMMSWAVLVVTYAIKQIELYGKLSDSMTVSATLMLVYITKFFWWEA
GYWNSMDIAHDRAGFYIVWGCLVWVPSVYTSPALYLVNHPVELGMPLAAAITAAGLLCIWINYDADLQRQTFRKTDGKAKVWGKVPNKIVATYVTEKGEKKQSLLLVSGWWAMARHFHYLPEISAAFF
WTVPALFSHPLPYFYVVFLTILLVDRAERDDKRCQTKYKKFWDEYKRTVPCKIIPHIF

>*Physcomitrella patens* 7-DR1-2

MGTRCTQSWPPQPPSATLLATLSWYTMVRADGSVEKACRHFFQFGIRGIKDVWPFPSQRACKLVGAFVLFEAILQALLPGERVMGPSTPVGNRPLYTRNGIPCFLITLGVYYLLWRERLFNPAMVYDIIGEIY
SVLICATYITTTLLYVKGHMAPCSSDWGSSGNVLGDFFWGMELCPRLSSVFDIKVFMNYRLGIMGWAILVISFAIKQYEVQGRVSDSLMVSSLLMLMYIAKFFWMEAAFGCSMDIAHDRAGWFMTYTCVT
WIPVVNSSVNLYLVTHPIQLGRRMASTIFLLGMLCIYVVVWDCDRQRQLFRNSHGKCQIWGRIPTKIQARHQTERGERKSLLLTAGWHIHYAPEIGAFFWTLPALFRHAMPYFYVTFLTALLIDRANRDDKYC
RVKYHKHWEAYSKKVRYKVVPRVF

>*Brachypodium distachyon* 7-DR1-1

MGKPKPSSAAPPPTRPSAASAPKTVHSALLTYTSMLSLLSLCPPFVILLWYTMVHADGSVVRTYEHLRDHGLLEGLKAIWPMPTMAAWKIIFGFALFEAVLQLLLPGKRFEGPISPSGNVPVYKANGLQAYA

VTLVTYLSMWWFGLFNPAIVYDHLGEIYSALVFGSFVFCIFLYIKGHVAPSSSDSGSSGNAIIDFYWGMELYPRIGKHFDIKVFTNCRFGMMSWAVLAVTYCIKQYEMNGRVADSMLVNTALMLIYVTKFF

WWESGYWCTMDIAHDRAGFYICWGCLVWVPSIYTSPGMYLVNHPVNLGPQLAVSILLAGILCIYINYDCDRQRQEFRRTNGKCSIWGKAPSKIVASYQTTAGETKTSLLLTSGWWGLSRHFHYVPEILSAF

FWTVPALFDHFLPYFYVIFLTILLLDRAKRDDDRCSSKYGKYWKMYCNKVPYRVVPGIY

>*Brachypodium distachyon* 7-DR1-2

MGKPKPSSAAPPPTRPSAASAPKTVHSALLTYTSMLSLLSLCPPFVILLWYTMVHADGSVVRTYEHLRDHGLLEGLKAIWPMPTMAAWKIIFGFALFEAVLQLLLPGKRFEGPISPSGNVPVYKANGLQAYA

VTLVTYLSMWWFGLFNPAIVYDHLGEIYSALVFGSFVFCIFLYIKGHVAPSSSDSGSSGNAIIDFYWGMELYPRIGKHFDIKVFTNCRFGMMSWAVLAVTYCIKQYEMNGRVADSMLVNTALMLIYVTKFF

WWESGYWCTMDIAHDRAGFYICWGCLVWVPSIYTSPGMYLVNHPVNLGPQLAVSILLAGILCIYINYDCDRQRQEFRRTNGKCSIWGKAPSKIVASYQTTAGETKTSLLLTSGWWGLSRHFHYVPEILSAF

FWTVPALFDHFLPYFYVIFLTILLLDRAKRDDDRCSSKYVRKVLEDVLQQSTIQGCSWYLLKILSG

>*Medicago truncatula* 7-DR1

MVESKKTETVHSPLVTYASMISLLTLAPPFVNLLWYTMTTAMDGSILKTFDYLNNNGLQGFLNLWPKPTLLSFQIIVVYAAFEALLQLLLPGETVYGPISPTGNRPFYKANGVAAYLVTLVTYVALWRFGIF

NPTIVYDHLGEIYSTLSFGSFIFCIFLYIKGHLAPSSTDSGSSGNIIIDFYWGMELYPRIGKYFDIKVFTNCRFGMMSWAVLALTYCIKQYEENGKVADSMLVNTTLMLVYVTKFFWWEAGYWNTMDIAHDR

AGFYICWGCLVWVPSVYTSPGMYLVNHPVNLGTQIEASYTTSSGETKRSLLLTSGWWRLARHFHYVPEILAAFFWTVPALFNHFLPYLYVIFLIILLFDRAKRDDDRCRSKYGKYWKLYCDRVAYRIIPGIY

>*Oryza sativa* 7-DR1

MAKPRASAAAAKAPASTPPKTVHSALVTYASMLSLLSLCPPFVILLWYTMVHADGSVVRAYEHLREHGVLEGLKAIWPMPTMAAWKIIFGFGLFEAALQLLLPGKRFEGPVSPSGNVPVYKANGLQAYAV

TLITYLSLWWFGIFNPAIVYDHLGEIYSALVFGSFVFCIFLYIKGHLAPSSSDSGSSGNVIIDFYWGMELYPRIGKHFDIKVFTNCRFGMMSWAVLAVTYCIKQYEMNGRVADSMLVNTALMLIYVTKFFWW

ESGYWCTMDIAHDRAGFYICWGCLVWVPSIYTSPGMYLVNHPVNLGPQLALSILLAGILCIYINYDCDRQRQEFRRTNGKCSIWGKAPSKIVASYQTTNGETKSSLLLTSGWWGLSRHFHYVPEILSAFFWT

VPALFDHFLPYFYVIFLTILLFDRAKRDDDRCSSKYGKYWKMYCNKVPCRVIPGIY

> *Dioscorea alata* 7-DR2

MVESKTVHSALITYTSMISLLSLCPPFVILLWYTMVHADGSVMQTFEYLKQNGLEGLKTIWPSPSLIAWKIIAVFGVFEAFLQLALPGKRFEGPVSPTGHVPVYKANGLQAYAVTLITYLGLWWFGIFNPAIV

YDHLGEIYSALVTGSLVFCVFLYIKGHLAPSSSDSGSSGNVIIDFYWGMELYPRIGKHFDIKVFTNCRFGMMSWAVLALTYCIKQYEQNGRVADSMLVNTALMLVYITKFFWWESGYWCTMDIAHDRAGF

YICWGCLVWVPSIYTSPGMYLVNHPVNLGTQLALSILAAGLLCIYINYDCDRQRQEFRRTNGKCKIWGKAPSKIVASYKTTKGETKTSLLLTSGWWGLARHFHYVPEISAAFFWTVPALFSHFLPYFYVIFLT

ILLFDRAKRDDDRCSSKYGKYWKMYCDKVPYRIVPGIY

>*Dioscorea alata* 7-DR1

MVESKTVHSALITYTSMISLLSLCPPFVILLWYTMVHADGSVMQTFEYLKQNGLEGLKTIWPSPSLIAWKIIAVFGVFEAFLQLALPGKRFEGPVSPTGHVPVYKANGLQAYAVTLITYLGLWWFGIFNPAIV
YDHLGEIYSALVTGSLVFCVFLYIKGHLAPSSSDSGSSGNVIIDFYWGMELYPRIGKHFDIKVFTNCRFGMMSWAVLALTYCIKQNGRVADSMLVNTALMLVYITKFFWWESGYWCTMDIAHDRAGFYIC
WGCLVWVPSIYTSPGMYLVNHPVNLGTQLALSILAAGLLCIYINYDCDRQRQEFRRTNGKCKIWGKAPSKIVASYKTTKGETKTSLLLTSGWWGLARHFHYVPEISAAFFWTVPALFSHFLPYFYVIFLTILL
FDRAKRDDDRCSSKYGKYWKMYCDKVPYRIVPGIY

>*Dioscorea cayenensis* 7-DR

MVESKTVHSALITYTSMISLLSLCPPFVILLWYTMVHADGSVMQTFEYLKQNGLEGLKTIWPTPSLIAWKIIAVFGAFEAFLQLALPGKRFEGPVSPTGHVPVYKANGLQAYAVTLITYLGLWWFGIFNPAIV
YDHLGEIYSALVTGSLVFCVFLYIKGHLAPSSSDSGSSGNVIIDFYWGMELYPRIGKHFDIKVFTNCRFGMMSWAVLALTYCIKQYEQNGQVADSMLVNTALMLVYITKFFWWESGYWCTMDIAHDRAGF
YICWGCLVWVPSIYTSPGMYLVNHPVNLGSQLALSILAAGLLCIYINYDCDRQRQEFRRTNGKCKIWGKAPSKGSIVASYKTTKGETKTSLLLTSGWWGLARHFHYMPEISAAFFWTVPALFSHFLPYFYVI
FLTILLFDRAKRDDDRCSSKYGKYWKMYCDKVQYRVIPGIY

>*Dioscorea polystachya* 7-DR

MVESKTVHSALITYTSMISLLSLCPPFVILLWYTMVHADGSVMQTFEYLKQNGLEGLKTIWPAPSLIAWKIIAVFGAFEAFLQLALPGKRFEGPVSPTGHVPVYKANGLQAYAVTLITYLGLWWFGIFNPAIV
YDHLGEIYSALVTGSLVFCVFLYIKGHLAPSSSDSGSSGNVIIDFYWGMELYPRIGKHFDIKVFTNCRFGMMSWAVLALTYCIKQYEQNGRVADSMLVNTALMLVYITKFFWWESGYWCTMDIAHDRAGF
YICWGCLVWVPSIYTSPGMYLVNHPVNLGTQLALSILAAGLLCIYINYDCDRQRQEFRRTNGKCKIWGKAPSKIVASYKTTKGETKTSLLLTSGWWGLARHFHYVPEISAAFFWTVPALFSHFLPYFYVIFLT
ILLFDRAKRDDDRCSSKYGKYWKMYCDRVPYRIIPGIY

Supplemental Table 15. **Predicted *cis*-acting elements in the *Dp7-DR* promoter.**

***Cis*-acting element**	**Sequence/Copies**	**Number**	**Function**
ABRE	ACGTG	11	cis-acting element involved in the abscisic acid responsiveness
ACE	GACACGTATG	1	cis-acting element involved in light responsiveness
AE-box	AGAAA	2	part of a module for light response
ARE	AAACCA	1	cis-acting regulatory element essential for the anaerobic induction
AT1-motif	AATTATTTTTTATT	1	cis-acting element involved in the abscisic acid responsiveness
Box 4	ATTAAT	9	part of a conserved DNA module involved in light responsiveness
Box III	atCATTTTCACt	1	protein binding site
CAAT-box	CAAAT/CCAAT	15	common cis-acting element in promoter and enhancer regions
CCAAT-box	CAACGG	1	MYBHv1 binding site
CGTCA-motif	CGTCA	2	cis-acting regulatory element involved in the MeJA-responsiveness
G-Box	CACGTG	3	cis-acting regulatory element involved in light responsiveness
G-box	CACGTC	10	cis-acting regulatory element involved in light responsiveness
GA-motif	ATAGATAA	2	part of a light responsive element
GATA-motif	AAGGATAAGG	1	part of a light responsive element
GC-motif	CCCCCG	1	enhancer-like element involved in anoxic specific inducibility
HD-Zip 1	CAAT(A/T)ATTG	1	element involved in differentiation of the palisade mesophyll cells
I-box	AGATAAGG	1	part of a light responsive element
LS7	CAGATTTATTTTTA	1	part of a light responsive element
LTR	CCGAAA	1	cis-acting element involved in low-temperature responsiveness
MBS	CAACTG	1	MYB binding site involved in drought-inducibility
TCA-element	CCATCTTTTT	2	cis-acting element involved in salicylic acid responsiveness
TCT-motif	TCTTAC	1	part of a light responsive element
TGACG-motif	TGACG	2	cis-acting regulatory element involved in the MeJA-responsiveness

Supplemental Table 16. **Primers used in this study.**

Name	Forward	Reverse
**RT-qPCR**		
DpACTIN	GAGCAAGGAAATCACAGCAC	TCAGGGAAGCCAAGATAGAG
Dp7-DR	TACCTTGGTCTCTGGTGGTTTGG	ATAATCACATTCCCAGAGGACCCA
DpbZIP12	AACATCTACGGAGAGGGTGCTTCGG	TCAATCCCCTCCCCTCCTTCCT
DpCYP94A4	ATGGAGTACTTACACGCGGC	TTCCTCGGCTGAAACACTCC
DpCYP94C1	CGTGCAGTCAGACTCCAAGT	TGCTCGAACAATGGACACGA
DpS3GT2	CGGCCTTAAAGCTGCATGTC	TTTCACTGCTCCGGTCACTC
**CDS or promoter amplification**		
*Dp7-DR*_CDS	ATGGTGGAATCCAAGACGGTG	ATCCCCGGGATGATCCTGTAC
*DpbZIP12*_CDS	ATGGCATCGTCGAGGGTGATGG	CTACCATTGCATAGATTGAGTCCTC
*DpCYP94A4*_CDS	ATGAACCCCATCTTCCTCCTT	TCATTCAGATGAACTTAAATCTCTATTCT
*DpCYP94C1*_CDS	ATGGATTCAATGGAAAGCATGGT	CTAGCATGAGCATGTGCCAT
*DpS3GT2*_CDS	ATGGCGACGAACGGCG	TCAAGCAAACCCGAAGCAT
*Dp7-DR*_Promoter	GAATTTCTAGAACCACAGCAAGTC	TCGGAAATCCCCGGAATCAA
**VIGS vector construct**		
TRV2-Dp7-DR_TRV	aaggttaccgaattctctagaTTCCGTAGAACAAATGGAAAGTG (*Xba*I site is underlined)	cgtgagctcggtaccggatccCTTCCAATATTTCCCATACTTGG (*Bam* HI site is underlined)
TRV2-DpbZIP12_TRV	aaggttaccgaattctctagaATGGCATCGTCGAGGGTGATGGCTT (*Xba*I site is underlined)	cgtgagctcggtaccggatccCACCTCCTCCTCCATCACAGCCCCA (*Bam*HI site is underlined)
TRV2-*DpCYP94A4*_TRV	gtgagtaaggttaccgaattcAGCCCCACCCACACCATCACCATG	cgtgagctcggtaccggatccGCCGGAGACGGCGGCGGAGGAAA
TRV2-*DpCYP94C1*_TRV	gtgagtaaggttaccgaattcATCAGGTTTGACCAGCTTCTTCTT	cgtgagctcggtaccggatccTCCATCCGGCCCATCGCGTAGGGG
TRV2-*DpS3GT2*_TRV	gtgagtaaggttaccgaattcAAAAGATTAAGATGCTAAATCGAA	cgtgagctcggtaccggatccTCAAAACAAATTCTTTGAAATTTG
**Overexpression vector construct**		
pSuper1300-*Dp7-DR*	caaatcgactctagaaagcttATGGTGGAATCCAAGACGGTG	catggtaccggatccactagtATCCCCGGGATGATCCTGTAC

pSuper1300-DpbZIP12	caaatcgactctagaaagcttATGGCATCGTCGAGGGT	catggtaccggatccactagtCTACCATTGCATAGATTGAGTCCTCC
pSuper1300-*DpCYP94A4*	caaatcgactctagaaagcttATGAACCCCATCTTCCTCCTT	catggtaccggatccactagtTCATTCAGATGAACTTAAATCTCTATTCT
pSuper1300-*DpCYP94C1*	caaatcgactctagaaagcttATGGATTCAATGGAAAGCATGGT	catggtaccggatccactagtCTAGCATGAGCATGTGCCAT
pSuper1300-*DpS3GT2*	caaatcgactctagaaagcttATGGCGACGAACGGCG	catggtaccggatccactagtTCAAGCAAACCCGAAGCAT
**Subcellular localization vector construct**		
35S:*Dp7-DR*_GFP	cagtcacctgcAAAACAACATGGTGGAATCCAAGACGGTGCAC (*Aar*I site is underlined)	cagtcacctgcAAAATACAGTAGATCCCCGGGATGATCCTG (*Aar*I site is underlined)
**Y1H vector construct**		
pHis2-*ProDp7-DR*	gaattcGAATTTCTAGAACCACAGCAAGTC (*EcoR*I site is underlined)	gattcgcgaacgcgtgagctcTCGGAAATCCCCGGAATCAA (*Sac*I site is underlined)
AD-DpbZIP12	gccatggaggccagtgaattcATGGCATCGTCGAGGGTGATGG (*EcoR*I site is underlined)	cagctcgagctcgatggatccCTACCATTGCATAGATTGAGTCCTC (*Bam*HI site is underlined)
**Dual-luciferase vector construct**		
pGreenII-0800-LUC-*proDp7-DR*	ggtatcgataagcttGAATTTCTAGAACCACAGCAAGTC (*Hind*III site is underlined）	agaactagtggatccTCGGAAATCCCCGGAATCAA (*Bam*HI site is underlined)
pGreenII-62-SK-DpbZIP12	agaactagtggatccATGGCATCGTCGAGGGTGATGGCTT (*Bam*HI site is underlined)	ggtatcgataagcttCTACCATTGCATAGATTGAGTCCTC (*Hind* III site is underlined）
